# Diterpene chemical space of *Aeollanthus buchnerianus* Briq. aerial part

**DOI:** 10.1007/s13659-024-00491-7

**Published:** 2025-01-02

**Authors:** Gabin T. M. Bitchagno, Nathan Reynolds, Monique S. J. Simmonds

**Affiliations:** Royal Botanic Gardens Kew, Richmond, London, TW9 3AE UK

**Keywords:** Metabolomic, *Aeollanthus buchnerianus*, Isopimarane, Abietane, Structure elucidation, Lamiaceae

## Abstract

The Plectranthinae clade, which includes genera such as *Plectranthus*, *Ocimum*, and *Aeollanthus*, is well known for its diverse array of diterpenoids. While numerous studies have deepened the understanding of diterpene diversity across the clade, *Aeollanthus* species remain underexplored, with only two studies focusing on their diterpene profiles. The NMR-based chemical profiling of the EtOAc leaf extract of the rocky and succulent species *Aeollanthus buchnerianus* Briq. reveals a range of diterpenes with isopimarane and abietane skeletons including several previously unreported analogues. Interestingly, the isolated compounds provided insights into the breakdown patterns of both diterpene classes by examining the product ions in their MS2 spectra. These data offer valuable information for evaluating the taxonomic position of this species in relation to other species within the clade.

**Graphical Abstract:**

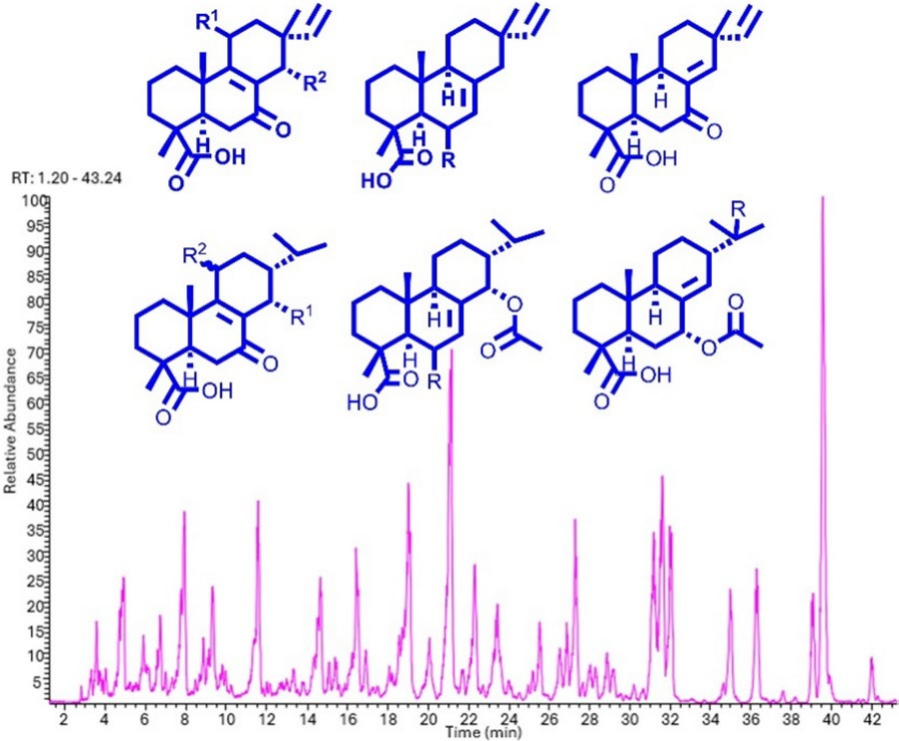

**Supplementary Information:**

The online version contains supplementary material available at 10.1007/s13659-024-00491-7.

## Introduction

Species of plants in the genus *Plectranthus* and in related genera in the family Lamiaceae including *Coleus* and *Ocimum* contain a range of well documented biologically active compounds [1, 2]. In contrast, other genera in the Plectranthinae clade like *Aeollanthus* [3] are not so well studied. For instance, a search using the keyword *Aeollanthus* in databases such as Scifinder^n^ and Reaxys® found only 50 and 52 publications, respectively. The genus contains about a hundred species distributed across sub-Sahara Africa excluding Madagascar and reputed for their resilience to harsh habitats like *A. parvifolius* [4] or for their strong fragrance and medicinal properties like *A. suaveolens* [5]*.* To date, most studies have focused on the composition of essential oils associated with insecticidal [6] and antimicrobial [7] properties.

Related genera often contain similar types of compounds often considered as signature molecules for the group. The Plectranthinae clade for instance are represented by diterpenoids as illustrated by the diversity of diterpenoids in *Plectranthus* [1]. With regard to *Aeollanthus*, only two studies have reported diterpenoids. In the late 90 s, Dellar et al. reported two abietane-type diterpenoids from *A. buchnerianus* [8]. More recently, Rijo et al. reported acyloxyisopimarane and other isopimarane diterpenoids from the aerial part of *A. rydingianus* [9]. One would deduce both isopimaranes and abietanes should represent markers of this genus too, but the hypothesis is yet to be tested.

Advances in mass spectrometry when combined with 1D NMR enable profiling of plant extracts for specific groups of compounds. *A. buchnerianus* in particular has not been investigated since the work of Dellar et al. [8]. It was therefore an opportunity to delve deeper into the profile of diterpenes in this species, resulting in the characterization of twenty diterpenes of which structures were confirmed following further NMR and MS analysis. Interestingly, the structures of eleven of the isolates were new to science including two isopimarane and nine abietane derivatives.

## Results and discussion

**Fig. 1:**
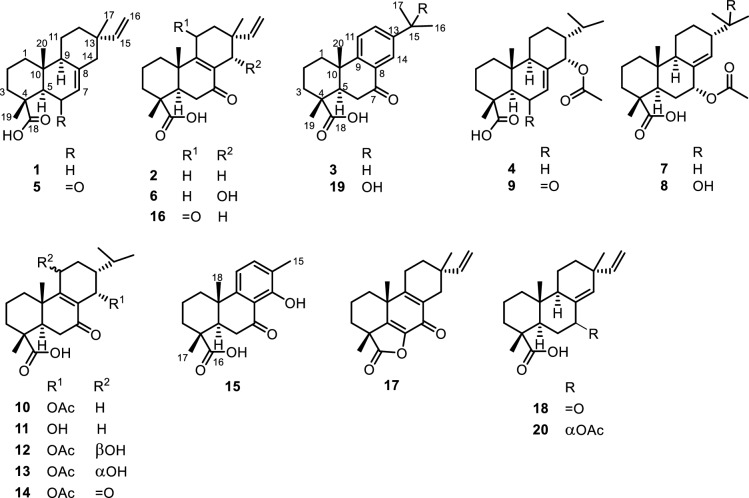
Structures of isolated compounds **1**–**20** from *Aeollanthus buchnerianus*

The EtOAc leaf extract of *A. buchnerianus* was fractionated, for NMR-based chemical profiling, into 8 fractions (E1-E8) including a column wash. Each fraction was then submitted to NMR analysis, mainly ^1^H,^1^H COSY, HSQC and HMBC, where each experiment was acquired beyond routine number of scans to allow minor components of the mixture to reveal their interactions. A relative consensus emerged after inspection of the major peaks informing on the class of compounds elicited by the plant and disclosing the main chemical characteristics of these compounds. Isopimaric acid (**1**) [10], 7-oxoisopimara-8,15-dien-18-oic acid (**2**), 7-oxodehydroabietic acid (**3**) [11] and 14*α*-acetoxyabiet-7-en-18-oic acid (**4**) [8] were among the main structures one could extract from the difficult complexity of NMR data of the plant fractions. Thus, isopimaranes and abietanes were hypothesized as main diterpenoid classes that should be expected from the species, aligning with the report from Dellar et al. [8] where compound **4** was then isolated alongside a second abietane analogue not isolated herein. Interestingly, in both classes of diterpenoids, the identified major components of the fractions were shown to conserve the same oxidation to carboxylic acid of one of the geminal methyls at C-4. Accordingly, the fractions were thoroughly inspected looking for angular methyls in the HSQC and ^1^H,^1^H COSY spectra that would correlate with a carbon at 175–185 ppm in the HMBC spectrum and would share with another methyl singlet, a HMBC interaction to a carbon at around 40–62 ppm for the position C-5 in both isopimaranes and abietanes. As a result, fractions E2, E3, E5 and E6 were selected for further analysis. The followed-up untargeted metabolomic analysis of those fractions by HPLC followed by NMR and LC–MS analysis led to the isolation and characterization of twenty diterpenes of which eleven were new chemistries (**5**–**15**) to science (Fig. 1).

**Fig. 2:**
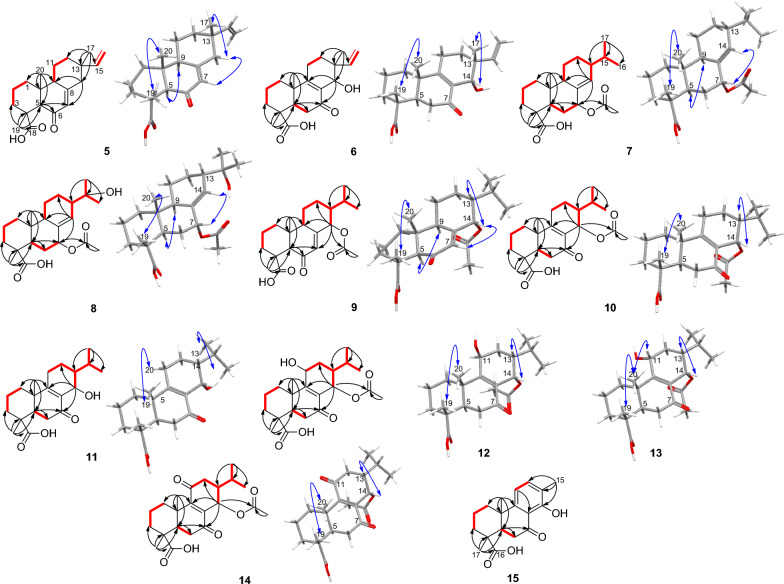
Selected COSY (red bold lines), HMBC (black arrows) and NOESY (blue double arrows) correlations in the new chemistries

Compound **5** was colorless upon drying. Its negative-mode HRESIMS exhibited a deprotonated molecular ion peak [M-H]^–^ at *m/z* 315.1961, corresponding to the molecular formula C_20_H_27_O_3_^–^ (calcd for C_20_H_27_O_3_^–^ *m/z* 315.1966). Its NMR spectra provided evidence of a vinyl group through the ABX system at *δ*_H_ 6.02 (dd, *J* = 10.6, 17.7 Hz, H-15)/* δ*_C_ 149.1 (C-15), 5.16 (br s, H-16b) and 5.11 (br s, H-16a)/* δ*_C_ 109.9 (C-16) and three angular methyls at *δ*_H_ 1.50 (s, H-19)/* δ*_C_ 17.1 (C-19), 1.11 (s, H-20)/* δ*_C_ 14.8 (C-20) and the allylic methyl at *δ*_H_ 1.05 (s, H-17)/* δ*_C_ 21.7 (C-17), characteristics of (iso)pimaric acid derivatives [12] (Tables 1 and 2). As expected, both methyls H-19 and H-20 showed HMBC interactions (Fig. 2) to C-5 (*δ*_C_ 59.3) while the HMBC spectrum evidenced additional interactions from H-19 to C-3 (*δ*_C_ 37.5), C-4 (*δ*_C_ 42.4) and the carboxylic group C-18 (*δ*_C_ 179.3) and from H-20 to C-1 (*δ*_C_ 37.6), C-10 (*δ*_C_ 38.9) and C-9 (*δ*_C_ 51.6). Likewise, the HMBC spectrum exhibited cross peaks from the allylic methyl H-17 to C-15, C-13 (*δ*_C_ 36.3), C-14 (*δ*_C_ 45.3) and C-12 (*δ*_C_ 35.1). The relative downfield shift of C-5, as compared to C-5 in isopimaric acid [12], was indicative of a nearby ketone group positioned at C-6 (*δ*_C_ 198.1) and supported by HMBC cross peaks from an olefin at *δ*_H_ 5.86 (s, H-7) and H-5 to C-6. The relative stereochemistry of **5** was established based on NOESY interactions from H-19 to H-20 and from H-9 to H-5 (Fig. 2). Most importantly, the stereochemistry at C-13 was defined using the method developed in the literature and summed up by Seca et al. [12] in a review work where a clear distinction of pimaranes (*α*CH_3_−17) and isopimaranes (*β*CH_3_−17) were discussed based on ^13^C NMR resonances of the methyl C-17. Indeed, C-17 is expected to resonate at ~ 29 ppm in ∆^8(14),15^ pimaradienes while it appears at ~ 26 ppm in ∆^8(14),15^ and at ~ 22 ppm in ∆^7,15^ isopimaradienes [12]. Therefore, compound **5** was an isopimarane as the methyl C-17 resonated at 21.7 ppm. Compound **5** was a new derivative with similar stereochemistry as compound **1** and characterized as *rel*−6-oxoisopimara-7,15-dien-18-oic acid.

**Table 1:** ^1^H (400 MHz) NMR spectroscopic data for compounds of **5**–**15** (*δ* in ppm, *J* in Hz)

No	**5**	**6**	**7**	**8**	**9**	**10**	**11**	**12**	**13**	**14**	**15**
1	1.34, m 1.95, m	1.29, m 1.86, m	1.72, m 1.80, m	1.74, m	1.37, m 1.84, m	1.54, m 1.88, m	1.54, m 1.88, m	1.62, m 2.26, m	1.97, m	1.28, m 2.77, m	1.87, m
2	1.88, m	1.67, m	1.59, m	1.59, m	1.71, m	1.64, m	1.74, m	1.77, m	1.77, m	1.72, m	1.83, m
3	1.92, m	1.67, m 1.77, m	1.72, m 1.80, m	1.73, m 1.83, m	1.63, m 1.76, m	1.76, m 1.85, m	1.75, m 1.83, m	1.78, m 1.83, m	1.77, m	1.75, m 1.83, m	1.76, m 1.85, m
4											
5	3.13, s	2.47, m	2.36, dd (2.5, 12.9)	2.37, m	3.04, s	2.60, m	2.66, m	2.57, m	2.66, dd (3.8, 13.8)	2.54, m	2.71, dd (3.3, 14.2)
6		2.24, m 2.47, m	1.20, m 1.72, m	1.22, m 1.71, m		2.33, dd (3.2, 17.4) 2.48, t (17.2)	2.30, m 2.50, m	2.36, m 2.57, m	2.38, dd (3.8, 17.6) 2.49, t (17.6)	2.54, m 2.40, m	2.46, dd (3.3, 18.1) 2.97, dd (14.2, 18.1)
7	5.86, s		5.29, br s	5.30, br s	6.08, d (2.8)						
8											
9	2.47, m		2.18, m	2.18, m	2.50, m						
10											
11	1.69, m 1.92, m	2.24, m	1.28, m 1.80, m	1.84, m	1.34, m 1.97, m	1.54, m 1.84, m	1.53, m 1.83, m	4.78, br s	4.54, br d (7.3)		6.92, d (8.0)
12	1.70, m	1.47, m	1.68, m 1.80, m	1.16, m 1.84, m	1.87, m	2.22, m 2.63, m	2.53, m 2.61, m	1.82, m	2.06, m 1.80, m	2.53, m 2.69, t (14.1)	7.48, d (8.0)
13			1.84, m	2.06, m	1.16, m	1.01, m	0.85, m	1.49, m	1.14, m	1.73, m	
14	2.36, m	4.21, br s	5.76, br s	5.96, br s	5.70, br s	6.25, br s	4.76, br d (3.1)	6.20, d (2.2)	6.41, br d (2.2)	6.32, br s	
15	6.02, dd (10.6, 17.7)	5.64, dd (11.1, 17.7)	1.57, m		1.53, m	1.50, m	1.76, m	1.54, m	1.56, m	1.62, m	2.29, s
16	5.11, br s 5.16, br s	4.96, m	0.85, d (4.7)^a^	1.16, s^a^	0.93, d (6.7)^a^	0.92, d (6.6)^a^	0.98, d (6.7)^a^	0.92, d (6.3)^a^	1.03, d (6.5)^a^	0.90, d (6.6)^a^	
17	1.05, s	1.08, s	0.88, d (5.0)^a^	1.22, s^a^	0.96, d (6.9)^a^	1.05, d (6.4)^a^	1.06, d (6.6)^a^	1.07, d (6.3)^a^	0.94, d (6.5)^a^	1.05, d (6.6)^a^	1.42, s
18											1.36, s
19	1.50, s	1.25, s	1.21, s	1.23, s	1.47, s	1.31, s	1.31, s	1.32, s	1.31, s	1.31, s	
20	1.11, s	1.16, s	0.80, s	0.81, s	0.94, s	1.16, s	1.17, s	1.34, s	1.09, s	1.34, s	
CO											
CH_3_			2.02, s	2.03, s	2.06, s	2.02, s		1.98, s	2.07, s	2.07, s	
OH											13.16, s

Data measured in CDCl_3_

^a^Interchangeable signals within the column

**Table 2:** ^13^C (100 MHz) NMR Spectroscopic Data for Compounds of **5**–**15** (*δ* in ppm)

No	**5**	**6**	**7**	**8**	**9**	**10**	**11**	**12**	**13**	**14**	**15**
1	37.6, CH_2_	34.5, CH_2_	36.9, CH_2_	37.6, CH_2_	37.8, CH_2_	35.3, CH_2_	35.3, CH_2_	34.4, CH_2_	33.7, CH_2_	34.0, CH_2_	36.4, CH_2_
2	18.5, CH_2_	17.9, CH_2_	18.0, CH_2_	17.9, CH_2_	17.6, CH_2_	17.9, CH_2_	17.9, CH_2_	17.7, CH_2_	17.9, CH_2_	17.7, CH_2_	18.5, CH_2_
3	37.5, CH_2_	36.3, CH_2_	36.9, CH_2_	36.7, CH_2_	37.5, CH_2_	36.1, CH_2_	36.0, CH_2_	36.3, CH_2_	36.3, CH_2_	36.1, CH_2_	35.9, CH_2_
4	42.4, C	46.1, C	46.7, C	46.5, C	42.7, C	46.3, C	46.3, C	46.4, C	46.1, C	46.3, C	45.8, C
5	59.3, CH	44.4, CH	42.4, CH	42.5, CH	59.0, CH	43.2, CH	43.8, CH	43.3, CH	44.3, CH	43.3, CH	43.6, CH
6	198.1, C	37.3, CH_2_	38.0, CH_2_	38.2, CH_2_	198.8, C	36.7, CH_2_	37.2, CH_2_	37.0, CH_2_	36.8, CH_2_	36.9, CH_2_	37.5, CH_2_
7	126.0, C	199.8, C	75.6, CH	75.7, CH	129.1, CH	196.5, C	195.0–198.0, C	198.2, C	197.0–199.0, C	198.2, C	203.0–190.0, C
8	161.6, C	131.6, C	135.3, C	134.0–136.0, C	155.6, C	130.7, C	129.0–131.0, C	132.2, C	131.0–134.0, C	141.0, C	110.0–130.0, C
9	51.6, CH	168.3, C	48.2, CH	47.6, CH	49.1, CH	170.8, C	169.7, C	165.4, C	168.7, C	156.4, C	154.6, C
10	38.9, C	39.1, C	37.8, C	37.6, C	39.4, C	39.0, C	38.9, C	38.6, C	38.8, C	38.6, C	37.4, C
11	19.6, CH_2_	23.0, CH_2_	22.0, CH_2_	21.9, CH_2_	23.9, CH_2_	19.6, CH_2_	19.8, CH_2_	65.0, CH	63.5, CH	201.8, C	113.5, CH
12	35.1, CH_2_	27.9, CH_2_	24.7, CH_2_	23.8, CH_2_	22.5, CH_2_	25.1, CH_2_	25.5, CH_2_	30.7, CH_2_	33.6, CH_2_	40.1, CH_2_	137.4, CH
13	36.3, C	38.5, C	41.8, CH	46.7, CH	47.4, C	44.7, CH	45.1, CH	38.9, CH	45.7, CH	46.3, CH	123.7, C
14	45.3, CH_2_	67.3, CH	134.3, CH	131.5, CH	73.3, CH	63.6, CH	61.4, CH	63.6, CH	64.2, CH	63.6, CH	160.5, C
15	149.1, C	143.2, C	31.9, CH	72.5, C	28.6, CH	27.9, CH	28.1, CH	27.1, CH	30.6, CH	28.5, CH	14.4, CH_3_
16	109.9, CH_2_	112.8, CH_2_	19.1, CH_3_^a^	26.0, CH_3_^a^	20.8, CH_3_^a^	20.2, CH_3_	20.7, CH_3_	20.0, CH_3_	20.6, CH_3_	19.8, CH_3_	178.5, C
17	21.7, CH_3_	24.0, CH_3_	19.4, CH_3_^a^	27.7, CH_3_^a^	20.7, CH_3_^a^	21.7, CH_3_	20.9, CH_3_	21.7, CH_3_	20.8, CH_3_	21.1, CH_3_	16.2, CH_3_
18	179.3, C	179.4, C	184.5, C	181.7, C	182.9, C	181.0, C	180.4, C	181.0, C	180.1, C	181.3, C	23.2, CH_3_
19	17.1, CH_3_	16.4, CH_3_	16.6, CH_3_	16.6, CH_3_	17.4, CH_3_	16.3, CH_3_	16.3, CH_3_	16.6, CH_3_	16.3, CH_3_	16.2, CH_3_	
20	14.8, CH_3_	18.3, CH_3_	14.3, CH_3_	14.1, CH_3_	15.3, CH_3_	19.6, CH_3_	19.8, CH_3_	20.8, CH_3_	18.5, CH_3_	18.4, CH_3_	
CO			170.5, C	170.7, C	169.6, C	169.6, C		169.6, C	168.6, C	169.3, C	
CH_3_			21.5, CH_3_	21.5, CH_3_	21.3, CH_3_	21.3, CH_3_		21.2, CH_3_	21.5, CH_3_	21.2, CH_3_	

Data measured in CDCl_3_

^a^Interchangeable signals within the column

Likewise, the negative-mode HRESIMS of compound **6** exhibited a deprotonated molecular ion peak [M − H]^–^ at *m/z* 331.1909, supporting the molecular formula C_20_H_27_O_4_^–^ (calcd for C_20_H_27_O_4_^–^ *m/z* 331.1915). Its NMR spectra also evidenced resonances of a vinyl group through the ABX system at *δ*_H_ 5.64 (dd, *J* = 11.1, 17.7 Hz, H-15)/* δ*_C_ 143.2 (C-15), 4.96 (m, H-16)/* δ*_C_ 112.8 (C-16) and three angular methyls at *δ*_H_ 1.25 (s, H-19)/* δ*_C_ 16.4 (C-19), 1.16 (s, H-20)/* δ*_C_ 18.3 (C-20) and the allylic methyl at *δ*_H_ 1.08 (s, H-17)/ *δ*_C_ 24.0 (C-17), characteristics of (iso)pimaric acid derivatives [12] (Tables 1 and 2). Similar HMBC cross peaks, as in compound **5**, supported a carboxylic group in compound **6** at C-18 (*δ*_C_ 179.4). The structure also exhibited a hydroxyl at C-14 through HMBC interactions (Fig. 2) from the allylic methyl H-17 to C-15, C-13 (*δ*_C_ 38.5), C-14 (*δ*_C_ 67.3) and C-12 (*δ*_C_ 27.9) and from H-14 to C-9, C-13 and C-7 (*δ*_C_ 199.8). Compound **6** was also classified as an isopimarane as the methyl C-17 resonated at 24.0 ppm. Further interactions were spotted in its NOESY spectrum from H-19 to H-20 and from H-14 to H-17 supporting the *β*-orientation of H-14 as depicted in the 3D representation of compound **6** (Fig. 2). Compound **6** was a new derivative with similar stereochemistry as compound **2** and characterized as *rel*−14*α*-hydroxy-7-oxoisopimara-8,15-dien-18-oic acid.

Compound **7** was colorless upon drying. Its negative-mode HRESIMS exhibited a deprotonated molecular ion peak [M − H]^–^ at *m/z* 361.2373, corresponding to the molecular formula C_22_H_33_O_4_^–^ (calcd for C_22_H_33_O_4_^–^ *m/z* 361.2384). Its ^1^H NMR and ^1^H,^1^H COSY spectra exhibited a large spin system involving the methylenes H-11 (*δ*_H_ 1.80/1.28) and H-12 (*δ*_H_ 1.80/1.68), the olefin H-14 (*δ*_H_ 5.76), the methines H-15 (*δ*_H_ 1.57) and H-9 (*δ*_H_ 2.18), and the secondary methyls H-16 (*δ*_H_ 0.85) and H-17 (*δ*_H_ 0.88) (Table 1). Further resonances supporting the abietane core structure for compound **7** also included two additional angular methyls H-19 (*δ*_H_ 1.21) and H-20 (*δ*_H_ 0.80). As expected, both methyl singlets showed HMBC interactions to C-5 (*δ*_C_ 42.4) while the HMBC spectrum evidenced additional interactions from H-19 to C-3 (*δ*_C_ 36.9), C-4 (*δ*_C_ 46.7) and the carboxylic group C-18 (*δ*_C_ 184.5) and from H-20 to C-1 (*δ*_C_ 36.9), C-10 (*δ*_C_ 37.8) and C-9 (*δ*_C_ 48.2). The HMBC spectrum also exhibited cross peaks (Fig. 2) from the methine H-15 to C-16, C-17, C-14 (*δ*_C_ 134.3) and C-8 (*δ*_C_ 135.3). Interestingly, a second spin system, adjacent to the olefin as judged by HMBC interactions from H-7 to C-9 and C-14 and from H-5 and H-6 to C-8, was evidenced in the ^1^H,^1^H COSY spectrum involving H-5, H-6 (*δ*_H_ 1.72/1.20) and H-7 (*δ*_H_ 5.29). Further cross peaks justified an acetyloxy group attached at C-7 from H-7 and an additional methyl singlet at *δ*_H_ 2.02 to the carbonyl at *δ*_C_ 170.5. The NOESY spectrum of **7** evidenced cross peaks from H-19 to H-20 and from H-5 to H-9. Most importantly, the spectrum exhibited interactions from H-7 to H-14 and from H-14 to H-13 informing on a planar orientation of H-7 and H-13 (Fig. 2). The axial positioning of the acetate at C-7 was further confirmed by the *γ*-shielding effect observed at C-5 as compared to compounds **1 **and **4**. Compound **7** was a new derivative with similar stereochemistry as compound **4** and characterized as *rel*−7*α*-acetyloxyabiet-8(14)-en-18-oic acid.

The negative-mode HRESIMS of compound **8** exhibited a deprotonated molecular ion peak [M − H]^–^ at *m/z* 377.2325, corresponding to the molecular formula C_22_H_33_O_5_^–^ (calcd for C_22_H_33_O_5_^–^ *m/z* 377.2333), 16 Da away from compound **7**. Indeed, like in **7**, NMR resonances supported the abietane core structure for compound **8**. However, compared to 7, the isopropyl at C-13 was replaced by a 2-hydroxypropyl as confirmed by HMBC cross peaks from the tertiary methyls H-16 (*δ*_H_ 1.16) and H-17 (*δ*_H_ 1.22) to an hydroxylated quaternary carbon at *δ*_C_ 72.5 (C-15) and the methine C-13 (*δ*_C_ 46.7). The relative configuration of compound **8** was also similar to that of compound **7** including the orientation of the acetate at C-7 also confirmed by its γ-gauche effect to C-5. Compound **8** was a new derivative with similar stereochemistry as compounds **4** and** 7** and characterized as *rel*−7*α*-acetyloxy-15-hydroxyabiet-8(14)-en-18-oic acid.

Compound **9** was colorless upon drying. Its negative-mode HRESIMS exhibited a deprotonated molecular ion peak [M − H]^–^ at *m/z* 375.2169, corresponding to the molecular formula C_22_H_31_O_5_^–^ (calcd for C_22_H_31_O_5_^–^ *m/z* 375.2177). Likewise, NMR resonances supported the abietane core structure for compound **9** including a carboxylic acid at C-18 (*δ*_C_ 179.2). The relative downfield shift of C-5, as compared to C-5 in 7-oxodehydroabietic acid (**3**), was indicative of a nearby ketone group positioned at C-6 (*δ*_C_ 198.8) and supported by HMBC cross peaks from an olefin at *δ*_C_ 6.08 (d, *J* = 2.8 Hz) and H-5 to C-6. Further cross peaks justified an acetyloxy group at C-14 supported by HMBC interactions from the methine H-15 to C-16, C-17 and C-14 (*δ*_C_ 73.3) and from H-14 and an additional methyl singlet at *δ*_H_ 2.06 to the carbonyl at *δ*_C_ 169.6. The relative stereochemistry of **9** was established as similar to that of compound **7** using the NOESY spectrum through same interactions from H-19 to H-20; from H-5 to H-9; from H-7 to H-14 and from H-14 to H-13 (Fig. 2). Compound **9** was a new derivative with similar stereochemistry as compound **4** and characterized as *rel*−14*α*-acetyloxy-6-oxoabiet-7-en-18-oic acid.

The negative-mode HRESIMS of compound **10** exhibited a deprotonated molecular ion peak [M − H]^–^ at *m/z* 375.2168, corresponding to the molecular formula C_22_H_31_O_5_^–^ (calcd for C_22_H_31_O_5_^–^ *m/z* 375.2177). As in compounds **7**–**9**, compound **10** was an abietane with an acetyloxy group at C-14 supported by HMBC interactions from the methine H-14 to C-9, C-8 (*δ*_C_ 130.7), C-7 (*δ*_C_ 196.5), C-15 (*δ*_C_ 27.9) and C-12 (*δ*_C_ 25.1); from the methine H-15 to C-16 (*δ*_C_ 20.2), C-17 (*δ*_C_ 21.7), C-14 (*δ*_C_ 63.6) and C-8 and from H-14 and an additional methyl singlet at *δ*_H_ 2.02 to the carbonyl at *δ*_C_ 169.6. The relative stereochemistry of **10** was established using the NOESY spectrum through interactions from H-19 to H-20 and from H-14 to H-13 supporting an *α*-orientation of the acetyloxy group (Fig. 2). Compound **10** was a new derivative characterized as *rel*−14*α*-acetyloxy-7-oxoabiet-8-en-18-oic acid.

The negative-mode HRESIMS of compound **11** exhibited a deprotonated molecular ion peak [M − H]^–^ at *m/z* 333.2069, supporting the molecular formula C_20_H_29_O_4_^–^ (calcd for C_20_H_29_O_4_^–^ *m/z* 333.2071), 42 Da less than **10**. Indeed, compared to **10**, the HMBC spectrum of **11** lacks the cross peak from the oximethine H-14 to the carbonyl of ester characteristic of acetyl group. The relative stereochemistry of **11** was established as similar to that of compound **10** using the NOESY spectrum through interactions from H-19 to H-20 and from H-14 to H-13 (Fig. 2). Compound **11** was a new derivative with similar stereochemistry as compound **10** and characterized as *rel*−14*α*-hydroxy-7-oxoabiet-8-en-18-oic acid.

The negative-mode HRESIMS of compound **12** exhibited a deprotonated molecular ion peak [M − H]^–^ at *m/z* 391.2116, corresponding to the molecular formula C_22_H_31_O_6_^–^ (calcd for C_22_H_31_O_6_^–^ *m/z* 391.2126). Compared to **10**, the ^1^H NMR and ^1^H,^1^H COSY spectra of compound **12** exhibited a large spin system (Fig. 2) involving two oximethines H-14 (*δ*_H_ 6.20) and H-11 (*δ*_H_ 4.78). Additionally, the HMBC spectrum exhibited cross peaks from both oximethines to C-9 and C-8 (*δ*_C_ 132.2); from H-14 to C-7 (*δ*_C_ 198.2), C-15 (*δ*_C_ 27.1) and C-12 (*δ*_C_ 30.7) and from the methine H-15 to C-16 (*δ*_C_ 20.0), C-17 (*δ*_C_ 21.7), C-14 (*δ*_C_ 63.6) and C-8. The absence of cross peak from H-11 to any carbonyl of esters justified positioning the acetyloxy group at C-14 of which characteristic signals were evidenced in the ^1^H and ^13^C NMR spectra at *δ*_H_ 1.98 (s, CH_3_CO)/ *δ*_C_ 21.2 and *δ*_C_ 169.6. The NOESY spectrum of **12** showed interactions (Fig. 2) from H-19 to H-20 and from H-14 to H-13 and H-17 supporting an *α*-orientation of the acetyloxy as in compound **10**. However, the spectrum also exhibited important cross peaks from H-11 to H_1_−1 (*δ*_H_ 1.62)/H_2_−1 (*δ*_H_ 2.26) and H-12 not to H-20 nor to H-14, not enough to conclude on the orientation of HO-11.

**Fig. 3:**
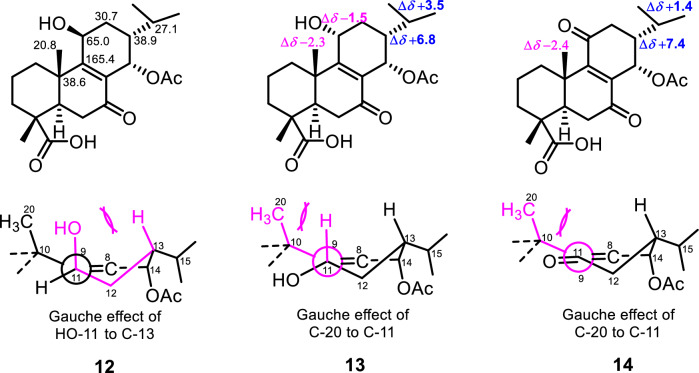
Chemical shift displacements and *γ*-gauche effects in compounds **12**–**14**

Fortunately, eluted 8 min after **12** was compound **13** with the negative-mode HRESIMS exhibiting the same mass characteristics as **12**. At first sight, its ^1^H NMR, ^1^H,^1^H COSY and HSQC spectra align well with that of compound **12**. Its HMBC and NOESY spectra in contrary disclosed some discrepancies much in the order-of-magnitude of some resonances. To start with, both secondary methyls H-16 (*δ*_H_ 1.03) and H-17 (*δ*_H_ 0.94) showed HMBC cross peaks (Fig. 2) to two methines at *δ*_C_ 30.6 for C-15 and 45.7 for C-13, downfield shifted by + 3.5 ppm and + 6.8 ppm, respectively, as compared to **12**. Likewise, the angular methyl C-20 (*δ*_C_ 18.5) rather exhibited an upfield displacement of –1.7 ppm as compared to **12**. The rationale behind all these shifts was suspected to relate to the orientation of the hydroxy group at C-11, mainly through *δ*- and *γ*-effects. Indeed, axial position of HO-11 eliminates the 1,3-diaxial interaction of H-11 with H-13 leading to a gauche effect of the hydroxy group to C-13 (Fig. 3) as well as it also jams into the methyl C-20 causing its downfield shift. Both effects are less pronounced when HO-11 is equatorial as the chemical shifts of C-13, C-15 and C-20 become comparable to that of **10**/**11** that lacks the hydroxy group at C-11 (Fig. 3). Moreover, lateral hydroxy group at C-11 also favored its upfield shift of –1.5 ppm from the gauche interaction of the methyl C-20 (Fig. 3). Accordingly, the hydroxy group at C-11 was established as periplanar in compound **12** and oriented differently in compound **13**. The orientation change of the hydroxy group in **13** was further supported by the clear interaction between H-11 (*δ*_H_ 4.54) and H-20 (*δ*_H_ 1.09), observed in the ROESY spectrum of **13**. Compounds **12** and **13** were epimers at C-11 and new derivatives characterized as *rel*−14*α*-acetyloxy-11*β*-hydroxy-7-oxoabiet-8-en-18-oic acid and *rel*−14*α*-acetyloxy-11*α*-hydroxy-7-oxoabiet-8-en-18-oic acid, respectively.

The negative-mode HRESIMS of compound **14** exhibited a deprotonated molecular ion peak [M − H]^–^ at *m/z* 389.1960, corresponding to the molecular formula C_22_H_29_O_6_^–^ (calcd for C_22_H_29_O_6_^–^, *m/z* 389.1960). Compound **14** was the 11-oxo derivative of compounds **12**–**13** as its ^13^C NMR spectrum evidenced a new resonance of a ketone at *δ*_C_ 201.8 (C-11) and only one oximethine resonance at *δ*_C_ 63.6 (C-14). Additionally, the HMBC spectrum exhibited cross peaks from H-14 to C-9 (*δ*_C_ 156.4) and C-8 (*δ*_C_ 141.0), C-7 (*δ*_C_ 198.3), C-15 (*δ*_C_ 28.5) and C-12 (*δ*_C_ 40.1) and from the methine H-15 to C-16 (*δ*_C_ 19.8), C-17 (*δ*_C_ 21.1), C-14 (*δ*_C_ 63.6) and C-8. Further HMBC cross peaks justified an acetyloxy group attached at C-14 from H-14 and an additional methyl singlet at *δ*_H_ 2.07 to the carbonyl at *δ*_C_ 169.3. The NOESY spectrum of **14** showed interactions (Fig. 2) from H-19 to H-20 and from H-14 to H-13 and H-17 supporting an *α*-orientation of the acetyloxy as in previous compounds. Indeed, despite the carbonyl at C-11, compound **14** exhibits the same steric hindrances encountered in **13** in relation to the gauche effect of the methyl C-20 to C-11 and vice versa and justified by comparable chemical shifts of C-13, C-15 and C-20 in both compounds (Fig. 3). Compound **14** was a new derivative with similar stereochemistry as compounds **10** and **11** and characterized as *rel*−14*α*-acetyloxy-7,11-dioxoabiet-8-en-18-oic acid.

Compound **15** was colorless upon drying. Its negative-mode HRESIMS exhibited a deprotonated molecular ion peak [M − H]^–^ at *m/z* 301.1442, corresponding to the molecular formula C_18_H_21_O_4_^–^ (calcd for C_18_H_21_O_4_^–^ *m/z* 301.1445). Its ^1^H NMR spectrum exhibited two doublets of aromatic protons H-11 (*δ*_H_ 6.92) and H-12 (*δ*_H_ 7.48), and three methyl singlets H-15 (*δ*_H_ 2.29), H-17 (*δ*_H_ 1.42) and H-18 (*δ*_H_ 1.36) (Table 1). As expected, compound **15** also exhibits a carboxylic acid at C-16 as judged by HMBC cross peaks similar to those observed for compounds **5**–**14**. Additionally, the HMBC spectrum evidenced cross peaks from the methyl H-15 to C-12, C-13 (*δ*_C_ 123.7) and C-14 (*δ*_C_ 160.5). A second spin system evidenced in the ^1^H,^1^H COSY spectrum involving H-5 and H-6 (*δ*_H_ 2.97/2.46) justified a carbonyl at C-7 and supported by a chelated hydroxy proton at *δ*_H_ 13.16 (s, OH-14). The relative stereochemistry of **15** was established using the NOESY spectrum through interactions from H-19 to H-20. Compound **15** was a new derivative with similar stereochemistry as compound **3** and characterized as *rel*−14-hydroxy-7-oxo-16,17-norabietic acid.

**Fig. 4:**
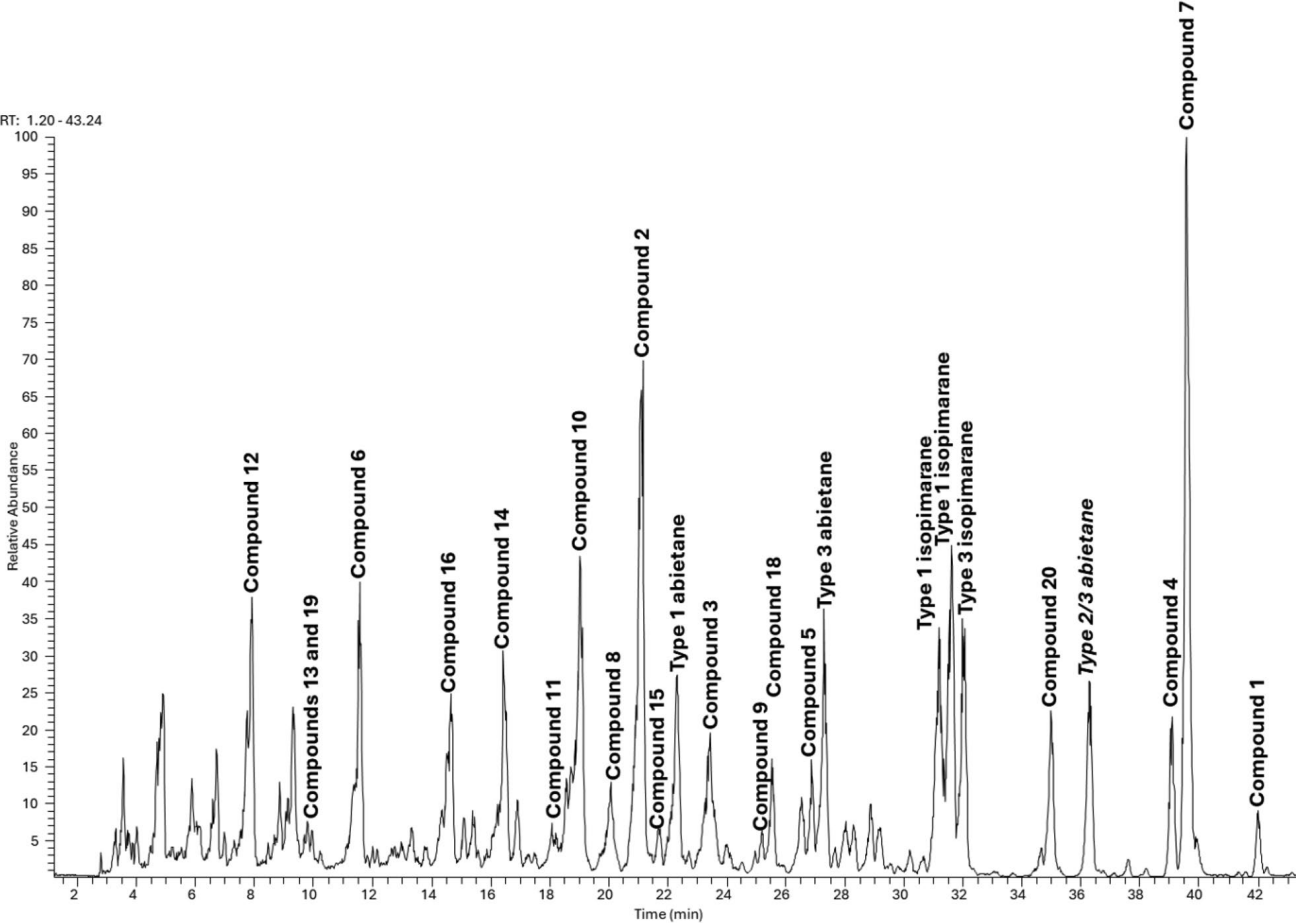
LC–MS base peak chromatogram of the EtOAc extract of *A. buchnerianus*

Further isopimaranes and abietanes were also isolated as known compounds including 7-oxoisopimara-8(14),15-dien-18-oic acid (**16**) [10], dabeshanensin B (**17**) [13], 7-oxoisopimara-8,15-dien-18-oic acid (**18**) [10], 15-hydroxydehydroabietic acid (**19**) [14] and 7*α*-acetoxysandaracopimaric acid (**20**) [15]. Wu et al. reported on the enantiomer of **2** [16]. The isolated compounds were used to annotate the LC-MSn base peak chromatogram of the studied EtOAc extract. Five of the major peaks in the chromatogram couldn’t match any of the isolated characteristics (Fig. 4). These compounds could have been missed out during HPLC isolation as they could be not UV-active. However, the literature is not very eloquent on the fragmentation patterns of isopimarane or abietane derivatives. Thus, the isolated compounds **1**–**20** were used as standards in an attempt to define the breakdown logic of both classes of diterpenes that would allow the full annotation of the extract diterpenes.

**Fig. 5:**
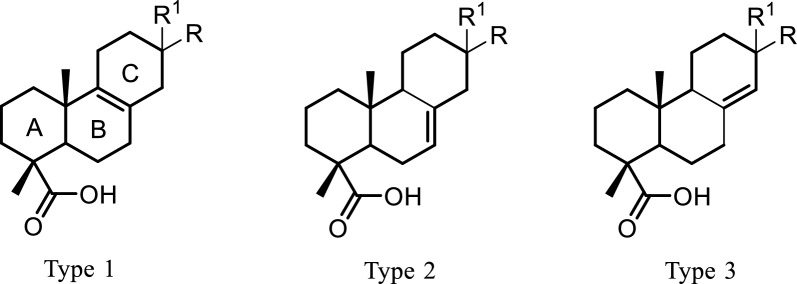
Diterpene types in isolated compounds

Isolated compounds were first clustered in three main types (Fig. 5) based on the olefin permutation within rings B and C, mainly ∆^8(9)^, ∆^7(8)^ or ∆^8(14)^, irrespective of their diterpene classes. Some of the compounds could also be paired, compounds **6** and **11**, as well as **7** and **20**, as they share the same oxidation of the ring system but differ by the diterpene class they belong to. Other compounds from the same class were paired as they represent different skeleton types, such as compounds **9** and **10**, **4** and **7**, and **2**, **5**, and **18**. Each of the pairs underwent LC-MSn analysis at a normalized collision energy of 40, and their product ions in MS2 spectra were compared to explore possible fragmentation patterns proper to diterpene types as well as classes by LC-MSn.

**Scheme 1:**
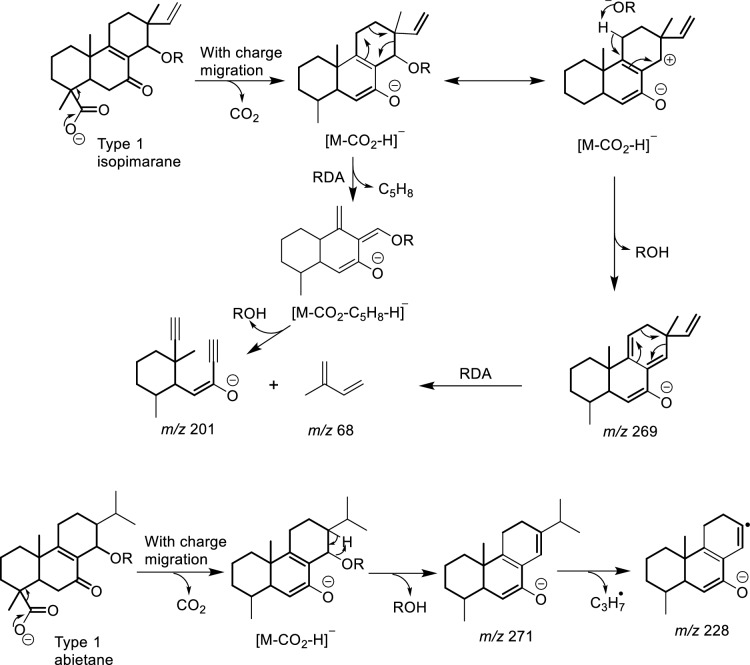
Retro Diels–Alder fragmentation mechanisms in *Aeollanthus* diterpenes

In this regard, the CID of the parent ion of compound **11** at *m/z* 333.2069 decays into a stable fragment ion at *m/z* 271.2067 (C_19_H_27_O^–^) following successive losses of H_2_O (*m/z* 315.1966) and CO_2_ (Scheme 1). Its isopimarane analogue, compound **6** (*m/z* 331.1909), also decays into a stable fragment ion at *m/z* 269.1910 (C_19_H_25_O^–^) after elimination of H_2_O and CO_2_ (Scheme 1). However, compound **6** alone unveiled a product ion at *m/z* 219.1391 (C_14_H_19_O_2_^–^) characteristic of a retro-Diels–Alder (RDA) fragmentation, leading to a neutral loss of *m/z* 68.0626 (C_5_H_8_) (Scheme 1), from the product ion at *m/z* 287.2016 (C_19_H_27_O_2_^–^) following CO_2_ loss from the parent [M − H]^–^ ion. The collision of both compounds was then induced at an increasing energy level from 5 to 90. None of the product ions of compound **11** evidenced the RDA decay featuring the loss of *m/z* 68.0626. Instead, the MS2 spectra of compound **11** only evidenced an isopropyl loss as the energy increases from a CID of 50 (Scheme 1). Similarly, the decomposition of compound **2** begins with a decarboxylation, producing an ion at *m/z* 271.2070 (C_19_H_27_O^–^), which then decays following two pathways. The first one led to a RDA on ring C resulting in a neutral loss of C_5_H_8_, like with compound **6**, while the other featured an *α*-elimination of methane, evidenced by the ion at *m/z* 255.1752 (C_18_H_23_O^–^). The decarboxylation of compounds **5** and **18** also produced a fragment ion at *m/z* 271.2070, which subsequently fragments further by releasing a neutral species of *m/z* 70.0786 (C_5_H_10_) instead.

The retro-Diels–Alder fragmentation on ring C appears to separate isopimarane from abietane derivatives. This occurs in both classes of type 1 compounds when the C-14 position is oxidized to either an alcohol or an alcoholate group like in compounds **4**, **6**, **9** and **10**–**11**. In such cases, the elimination of water or acetic acid extends existing conjugation in abietane analogues that prevents the RDA fragmentation from occurring (Scheme 1). The impossibility of the same elimination reaction in isopimarane derivatives results in a charge migration fragmentation, after the *α*-cleavage of hydroxy or acetate leading to the elimination of methylbutadiene by RDA (Scheme 1). In case position C-14 is not oxidized like in compounds **2** and **17**, isopimarane derivatives of type 1 also undergo a neutral loss of methylbutadiene.

On the other hand, the CID of the parent ions of compounds **4** and **7** at *m/z* 361.2374 (C_22_H_33_O_4_^–^) showed similar product ions at *m/z* 301.2171 (C_20_H_29_O_2_^–^) after the *α*-elimination of acetic acid. However, in this case, the decarboxylation fragmentation reaction resulted in a neutral loss of formic acid, leading to the formation of an ion at *m/z* 255.2119 (C_19_H_27_^–^). The same decomposition was also observed in the case of compounds **1** and **20** of types 2 and 3. This represents a key feature in distinguishing between types 2 and 3 diterpenes and those of type 1. At an increase in energy level from 5 to 90, both compounds **4** and **7** could not be differentiated. Only their ability to undergo in-source release of acetic acid during ionization seems to differ. Compound **4** showed both the deprotonated ion at *m/z* 361.2374 and the in-source loss of acetic acid ion at *m/z* 301.2171 with comparable abundances while the latter was almost inexistant in the MS1 spectrum of compound **7** (Fig. S82). However, compound **9** with similar substitution as in compound **4** failed to show similar ability to in-source fragmentation.

**Scheme 2:**
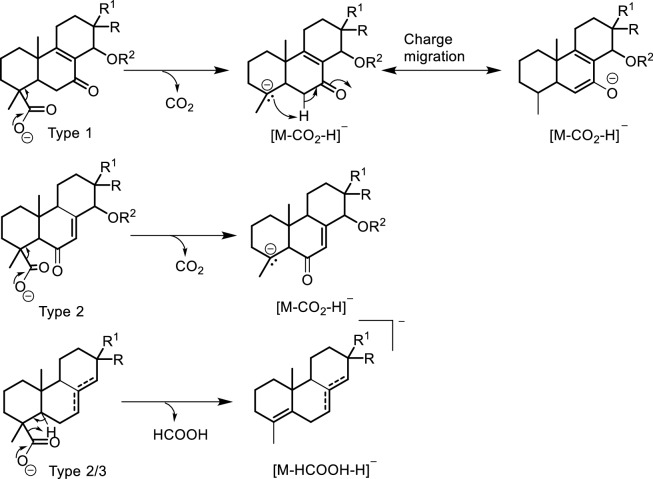
Decarboxylation reaction mechanisms in *Aeollanthus* diterpenes

The decarboxylation process and the releasing entity seem to differ with the diterpene types. In the case of types 1 and 3 with a carbonyl at C-7, CO_2_ is released through an *α*-cleavage with charge migration towards the carbonyl (Scheme 2) as portraited in the breakdown of compounds **2,** **3**, **6**, **10**–**14**, **15,** **16** and **18**. The decarboxylated entity and mechanism remain the same, except for the charge migration, when the carbonyl is located at C-6 like in type 2 compounds **5** and **9** (Scheme 2). However, in the absence of a carbonyl group at C-6 or C-7 like in compounds **1**, **4**, **7,** **8** and **20**, the decarboxylated body in diterpene types 2 and 3 is formic acid articulated here through a remote hydrogen fragmentation mechanism (Scheme 2).

Based on the two main features by which one should assign the olefin permutation type and the diterpene classes in *A. buchnerianus*, the five major peaks of the chromatogram missed during isolation were tentatively assigned diterpenes class and type (Fig. 4). Overall, irrespective of the diterpene types, the anticipated dissociation of CO_2_ from any of the CID of the parent ions of isolated compounds **1**–**20** was a second event, occurring after the breakdown of acetic acid, in acetylated derivatives and was prioritized otherwise. Moreover, none of the diterpenes were hydroxylated further or even glycosylated as none of the above patterns were identified in the 60% MeOH extract of the plant. The alignment of these characteristics with the chemistry as it develops in the plant could not be assessed comparatively given that only one chemical study of this plant exists to date.

Our findings also demonstrate that the diterpenes of *A. buchnerianus* possess distinct characteristics compared to those of *Plectranthus* and share greater similarity with analogues in *Tetradenia*, the two most extensively studied genera in the clade. Mainly, C-18/19 oxidized abietanes are rare in *Plectranthus*, and none of the abietane subclasses identified in *Plectranthus* were observed in *A. buchnerianus* [1, 2]. This contrasts with other genera within the clade such as *Tetradenia* which synthesizes royleanones, a major class of diterpenes commonly found in *Plectranthus*. The diterpenes of *A. buchnerianus* are more aligned with those described in *Tetradenia*. Both genera share similar diterpene classes, including abietanes and isopimaranes, as well as comparable double bond positions and oxidized carbons in the skeleton (C-6, C-7, C-14, and C-11) [17, 18]. The primary distinction lies in the extent of oxidation. In *A. buchnerianus*, the skeleton undergoes more advanced oxidation, particularly resulting in ketone groups at C-6, C-7, and C-11, while in *Tetradenia*, oxidation generally stops at the alcohol level [17, 18]. Worth mentioning as well, the diterpenes of this study are likely not enantiomeric knowing that enantiomeric diterpenes have not been reported from the Plectranthinae clade yet [1, 2, 17, 18]. The present results enrich the knowledge about the chemical diversity of diterpenoids in *A. buchnerianus* and open the opportunity to review whether other species in the genera contain similar compounds.

## Experimental methods

### General experimental procedure

LC–MS grade solvents (acetonitrile, methanol) and formic acid were obtained from Fisher Scientific (Loughborough, UK) and milliQ water was used for HPLC and LC–MS analysis. NMR spectra were acquired on a Bruker Avance-III (^1^H NMR: 400 MHz and ^13^C NMR: 100.1 MHz) spectrometer equipped with a 5 mm cryoprobe. Chemical shifts were referenced to residual solvent signals and reported in parts per million (ppm). Spectra were processed using Bruker NMR academic Topspin software. Mass spectra were collected on a Orbitrap Exploris mass spectrometer, equipped with a Vanquish diode array detector (VH-D10) coupled to an Orbitrap Exploris 120 with a heated ESI source (Thermo Scientific, Germany), acquired in both negative and positive modes with a resolution of 60,000 over *m/z* 125–1800 under various acquisition parameters like source voltages, sheath gas, auxiliary gas, sweep gas and capillary temperature set to 2.5 kV (negative mode) and 3.5 kV (positive mode), 50 (arbitrary units), 10 (arbitrary units), 1 (arbitrary units) and 350 °C, respectively. Automatic MS–MS fragmentation was performed on top four ions of the TIC using an isolation width of *m/z* 2. High-energy C-trap dissociation with a normalized collision energy of 40 and an activation time of 0.1 ms was served to fragment ions. Collected data were inspected using Xcalibur v. 4.2.47 (Thermo Fisher Scientific). Chemical profiling of extracts was conducted on a Biotage^®^ Isolera One system for splitting extracts into small fractions and a Waters Alliance 2695 HPLC system for isolation of compounds. A reversed-phase Disovery HS C-18 column (5 μm, 10 mm × 250 mm i.d., Supelco, UK) maintained at 35 °C served in compounds isolation and purification over gradient of acetonitrile + 0.1% formic acid (A) and water (B). The relative abundance of compounds in mixtures was evaluated via integration of isolated characteristic multiplets in the ^1^H NMR spectra.

### Plant material

The whole plant of *Aeollanthus buchnerianus* was collected from the living collection at RBG Kew; accession No 1976–2374. The plant tissues were freeze-dried, milled to fine powders, and kept in the dark for further uses.

### Extraction and isolation

Part of the milled plant materials (26.0 g) was serial extracted in solvents with increasing-polarities starting with *n*-hexane, then EtOAc and 60% MeOH affording dried extracts of 469.1 mg, 789.5 mg and 5.0 g, respectively. The EtOAc extract was split into seven small fractions (E1-E7) on the Biotage Isolera One system using a stepwise gradient of EtOAc in hexane starting from 100% hexane to 100% EtOAc, 10% increment and 3 column-volumes (CV) at each floor. The fractions were dissolved in CDCl_3_ and submitted to ^1^H NMR then to 2D NMR for chemical profiling. As rightly put above, only fractions E2, E3, E5 and E6 were further prepared for compound isolation. As E2 and E3 then E5 and E6 showed comparable compositions, they were combined to two main fractions E2 + 3 (110 mg) and E5 + 6 (106 mg). Fraction E2 + 3 was dissolved in 2 mL of ACN and injected into the Waters system, eluting with a constant flow rate of 2 mL/min of a linear gradient of acetonitrile (B) in water (A) (0–5 min, 35% B; 5–55 min, 35–55% B, 55–65 min, 100% B and 65–75 min, 35% B). Compounds were detected at 210, 254, 300 and 354 nm and collected by time into glass tubes. Cumulative fractions from eighteen injections of 100 *μ*L each were collected and dried using a GeneVac concentrator (Genevac, Suffolk, UK). Likewise, fraction E5 + 6 was eluted with a linear gradient of acetonitrile (B) in water (A) (0–5 min, 35% B; 5–55 min, 35 to 55% B, 55–65 min, 100% B and 65–75 min, 35% B). Compounds were detected, collected and dried under the same conditions. Fraction E2 + 3 afforded 13 compounds including 12 diterpenes: **1** (4.5 mg), **2** (3.2 mg), **3** (1.6 mg), **5** + **17** + **18** (25:25:50, 1.1 mg), **4** (6.0 mg), **7** (11.5 mg), **9** (2.0 mg), **15** + **16** (36:64, 0.8 mg) and **20** (0.8 mg) whereas fraction E5 + 6 elicited 8 more diterpenes: **6** (1.9 mg), **8** + **11** (53:47, 0.8 mg), **10** (1.2 mg), **12** (2.4 mg), **13** (0.6 mg), **14** (2.6 mg), **19** (1.1 mg) and four miscellaneous. Compounds isolated as mixtures were not further purified.

### *rel*-6-Oxoisopimara-7,15-dien-18-oic acid (5)

Colorless oil; UV λ_max_ 222, 259 nm; ^1^H and ^13^C NMR (CDCl_3_), see Tables 1 and 2; ( −)-HRESIMS *m/z* 315.1961 [M − H]^−^ (calcd for C_20_H_27_O_3_^−^, *m/z* 315.1966).

### *rel*-14*α*-Hydroxy-7-oxoisopimara-8,15-dien-18-oic acid (6)

Colorless oil; UV λ_max_ 218, 245 nm; ^1^H and ^13^C NMR (CDCl_3_), see Tables 1 and 2; ( −)-HRESIMS *m/z* 331.1909 [M − H]^−^ (calcd for C_20_H_27_O_4_^−^, *m/z* 331.1915).

### *rel*-7*α*-Acetyloxyabiet-8(14)-en-18-oic acid (7)

Colorless oil; UV λ_max_ 200 nm; ^1^H and ^13^C NMR (CDCl_3_), see Tables 1 and 2; ( −)-HRESIMS *m/z* 361.2373 [M − H]^−^ (calcd for C_22_H_33_O_4_^−^, *m/z* 361.2384).

### *rel*-7*α*-Acetyloxy-15-hydroxyabiet-8(14)-en-18-oic acid (8)

Colorless oil; UV λ_max_ 200 nm; ^1^H and ^13^C NMR (CDCl_3_), see Tables 1 and 2; ( −)-HRESIMS *m/z* 377.2325 [M − H]^−^ (calcd for C_22_H_33_O_5_^−^, *m/z* 377.2333).

### *rel*-14*α*-Acetyloxy-6-oxoabiet-7-en-18-oic acid (9)

Colorless oil; UV λ_max_ 222 nm; ^1^H and ^13^C NMR (CDCl_3_), see Tables 1 and 2; ( −)-HRESIMS *m/z* 375.2169 [M − H]^−^ (calcd for C_22_H_31_O_5_^−^, *m/z* 375.2177).

### *rel*-14*α*-Acetyloxy-7-oxoabiet-8-en-18-oic acid (10)

Colorless oil; UV λ_max_ 218, 247 nm; ^1^H and ^13^C NMR (CDCl_3_), see Tables 1 and 2; ( −)-HRESIMS *m/z* 375.2168 [M − H]^−^ (calcd for C_22_H_31_O_5_^−^, *m/z* 375.2177).

### *rel*-14*α*-Hydroxy-7-oxoabiet-8-en-18-oic acid (11)

Colorless oil; UV λ_max_ 214 nm; ^1^H and ^13^C NMR (CDCl_3_), see Tables 1 and 2; ( −)-HRESIMS *m/z* 333.2069 [M − H]^−^ (calcd for C_20_H_29_O_4_^−^, *m/z* 333.2071).

### *rel*-14*α*-Acetyloxy-11*β*-hydroxy-7-oxoabiet-8-en-18-oic acid (12)

Colorless oil; UV λ_max_ 218 nm; ^1^H and ^13^C NMR (CDCl_3_), see Tables 1 and 2; ( −)-HRESIMS *m/z* 391.2116 [M − H]^−^ (calcd for C_22_H_31_O_6_^−^, *m/z* 391.2126).

### *rel*-14*α*-Acetyloxy-11*α*-hydroxy-7-oxoabiet-8-en-18-oic acid (13)

Colorless oil; UV λ_max_ 214 nm; ^1^H and ^13^C NMR (CDCl_3_), see Tables 1 and 2; ( −)-HRESIMS *m/z* 391.2116 [M − H]^−^ (calcd for C_22_H_31_O_6_^−^, *m/z* 391.2126).

### *rel*-14*α*-Acetyloxy-7,11-dioxoabiet-8-en-18-oic acid (14)

Colorless oil; UV λ_max_ 214 nm; ^1^H and ^13^C NMR (CDCl_3_), see Tables 1 and 2; ( −)-HRESIMS *m/z* 389.1960 [M − H]^−^ (calcd for C_22_H_29_O_6_^−^, *m/z* 389.1970).

### *rel*-14-Hydroxy-7-oxo-16,17-norabietic acid (15)

Colorless oil; UV λ_max_ 218, 266, 350 nm; ^1^H and ^13^C NMR (CDCl_3_), see Tables 1 and 2; ( −)-HRESIMS *m/z* 301.1442 [M − H]^−^ (calcd for C_18_H_21_O_4_^−^, *m/z* 301.1445).

## Supplementary Information

Supplementary material 1

## Data Availability

The NMR and LC–MS raw data generated during and/or analysed during the current study are available from the corresponding author on request.
