# Supplementary material for: Diterpene chemical space of Aeollanthus buchnerianus Briq. aerial part
Source: Nat Prod Bioprospect. 2025 Jan 2;15(1):6. doi: 10.1007/s13659-024-00491-7 (PMC11695651; doi:10.1007/s13659-024-00491-7)
Supplement: Supplementary file 1 — Supplementary material 1 [file 13659_2024_491_MOESM1_ESM.docx]

**SUPPLEMENTARY IFORMATION**

**Diterpene Chemical Space of *Aeollanthus buchnerianus* Briq. Areal Part**

Gabin T.M. Bitchagno*, Nathan Reynolds, Monique S.J. Simmonds*

*Royal Botanic Gardens, Kew, Richmond, London, TW9 3AE*

Correspondences: [g.bitchagnombahbou@kew.org](mailto:g.bitchagnombahbou@kew.org) and [m.simmonds@kew.org](mailto:m.simmonds@kew.org)

TABLE OF CONTENTS

Figure S1. UV spectra of the mixture of **5**+**17**+**18**

Figure S2. Full (-)-HRESI mass and MS2 spectra of **5**

Figure S3. ^1^H NMR spectrum (400 MHz, CDCl_3_) of the mixture of **5**+**17**+**18**

Figure S4. ^1^H,^1^H COSY spectrum of the mixture of **5**+**17**+**18**

Figure S5. HMBC spectrum of the mixture of **5**+**17**+**18**

Figure S6. HSQC spectrum of the mixture of **5**+**17**+**18**

Figure S7. NOESY spectrum of the mixture of **5**+**17**+**18**

Figure S8. TOCSY spectrum of the mixture of **5**+**17**+**18**

Figure S9. UV spectrum of **6**

Figure S10. Full (-)-HRESI mass and MS2 spectra of **6**

Figure S11. ^1^H NMR spectrum (400 MHz, CDCl_3_) of **6**

Figure S12. ^13^C NMR spectrum (100 MHz, CDCl_3_) of **6**

Figure S13. ^1^H,^1^H COSY spectrum of **6**

Figure S14. HSQC spectrum of **6**

Figure S15. HMBC spectrum of **6**

Figure S16. UV spectrum of **7**

Figure S17. Full (-)-HRESI mass and MS2 spectra of **7**

Figure S18. ^1^H NMR spectrum (400 MHz, CDCl_3_) of **7**

Figure S19. ^13^C NMR spectrum (100 MHz, CDCl_3_) of **7**

Figure S20. ^1^H,^1^H COSY spectrum of **7**

Figure S21. HSQC spectrum of **7**

Figure S22. HMBC spectrum of **7**

Figure S23. NOESY spectrum of **7**

Figure S24. UV spectrum of the mixture of **8**+**11**

Figure S25. Full (-)-HRESI mass and MS2 spectra of **8**

Figure S26. ^1^H NMR spectrum (400 MHz, CDCl_3_) of the mixture of **8**+**11**

Figure S27. ^1^H,^1^H COSY spectrum of the mixture of **8**+**11**

Figure S28. HSQC spectrum of the mixture of **8**+**11**

Figure S29. HMBC spectrum of the mixture of **8**+**11**

Figure S30. UV spectrum of **9**

Figure S31. Full (-)-HRESI mass and MS2 spectra of **9**

Figure S32. ^1^H NMR spectrum (400 MHz, CDCl_3_) of **9**

Figure S33. ^13^C NMR spectrum (100 MHz, CDCl_3_) of **9**

Figure S34. ^1^H,^1^H COSY spectrum of **9**

Figure S35. HMBC spectrum of **9**

Figure S36. HSQC spectrum of **9**

Figure S37. NOESY spectrum of **9**

Figure S38. UV spectrum of **10**

Figure S39. Full (-)-HRESI mass and MS2 spectra of **10**

Figure S40. ^1^H NMR spectrum (400 MHz, CDCl_3_) of **10**

Figure S41. ^13^C NMR spectrum (100 MHz, CDCl_3_) of **10**

Figure S42. ^1^H,^1^H COSY spectrum of **10**

Figure S43. HMBC spectrum of **10**

Figure S44. HSQC spectrum of **10**

Figure S45. NOESY spectrum of **10**

Figure S46. UV spectrum of the mixture of **8**+**11**

Figure S47. Full (-)-HRESI mass and MS2 spectra of **11**

Figure S48. ^1^H NMR spectrum (400 MHz, CDCl_3_) of the mixture of **8**+**11**

Figure S49. ^1^H,^1^H COSY spectrum of the mixture of **8**+**11**

Figure S50. HSQC spectrum of the mixture of **8**+**11**

Figure S51. HMBC spectrum of the mixture of **8**+**11**

Figure S52. UV spectrum of **12**

Figure S53. Full (-)-HRESI mass and MS2 spectra of **12**

Figure S54. ^1^H NMR spectrum (400 MHz, CDCl_3_) of **12**

Figure S55. ^13^C NMR spectrum (100 MHz, CDCl_3_) of **12**

Figure S56. ^1^H,^1^H COSY spectrum of **12**

Figure S57. HMBC spectrum of **12**

Figure S58. HSQC spectrum of **12**

Figure S59. NOESY spectrum of **12**

Figure S60. UV spectrum of **13**

Figure S61. Full (-)-HRESI mass and MS2 spectra of **13**

Figure S62. ^1^H NMR spectrum (400 MHz, CDCl_3_) of **13**

Figure S63. ^1^H,^1^H COSY spectrum of **13**

Figure S64. HSQC spectrum of **13**

Figure S65. HMBC spectrum of **13**

Figure S66. NOESY spectrum of **13**

Figure S67. UV spectrum of **14**

Figure S68. Full (-)-HRESI mass and MS2 spectra of **14**

Figure S69. ^1^H NMR spectrum (400 MHz, CDCl_3_) of **14**

Figure S70. ^13^C NMR spectrum (100 MHz, CDCl_3_) of **14**

Figure S71. ^1^H,^1^H COSY spectrum of **14**

Figure S72. HMBC spectrum of **14**

Figure S73. HSQC spectrum of **14**

Figure S74. NOESY spectrum of **14**

Figure S75. UV spectrum of the mixture of **15**+**16**

Figure S76. Full (-)-HRESI mass and MS2 spectra of **15**

Figure S77. ^1^H NMR spectrum (400 MHz, CDCl_3_) of the mixture of **15**+**16**

Figure S78. ^1^H,^1^H COSY spectrum of the mixture of **15**+**16**

Figure S79. HMBC spectrum of the mixture of **15**+**16**

Figure S80. HSQC spectrum of the mixture of **15**+**16**

Figure S81. TOCSY spectrum of the mixture of **15**+**16**

Figure S82. MS1 comparison of compounds **4** and **7**

Figure S83. MS1 and MS2 of annotated diterpene types of the chromatogram


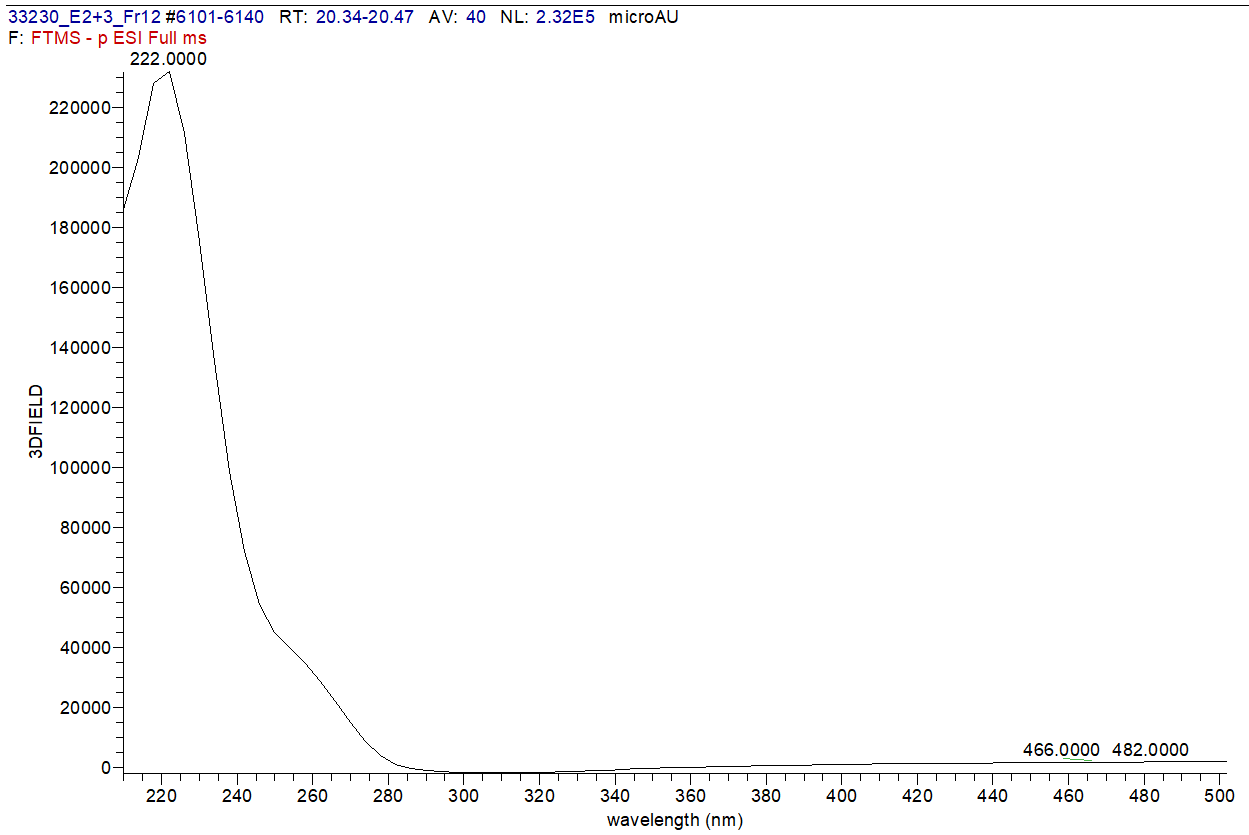


Figure S1. UV spectra of the mixture of **5**+**17**+**18**


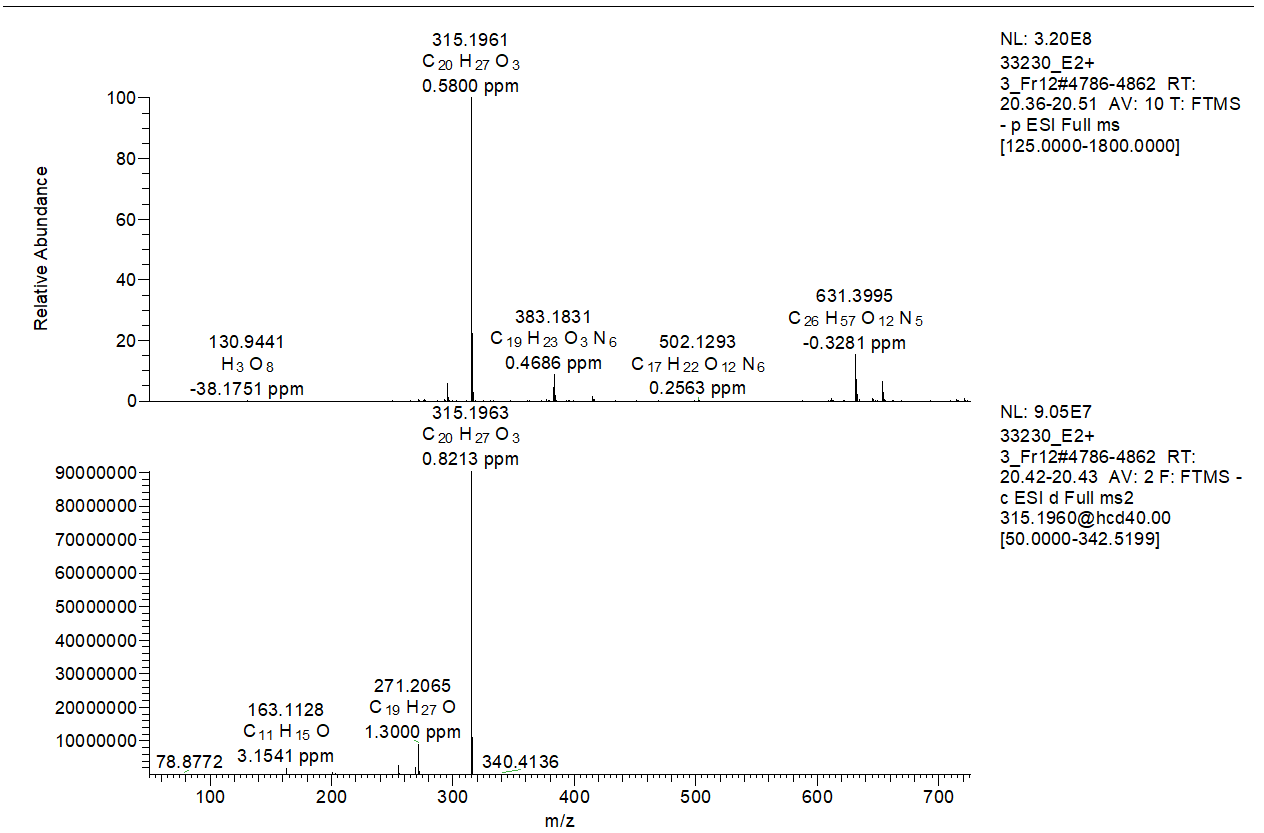


Figure S2. Full (-)-HRESI mass and MS2 spectra of **5**


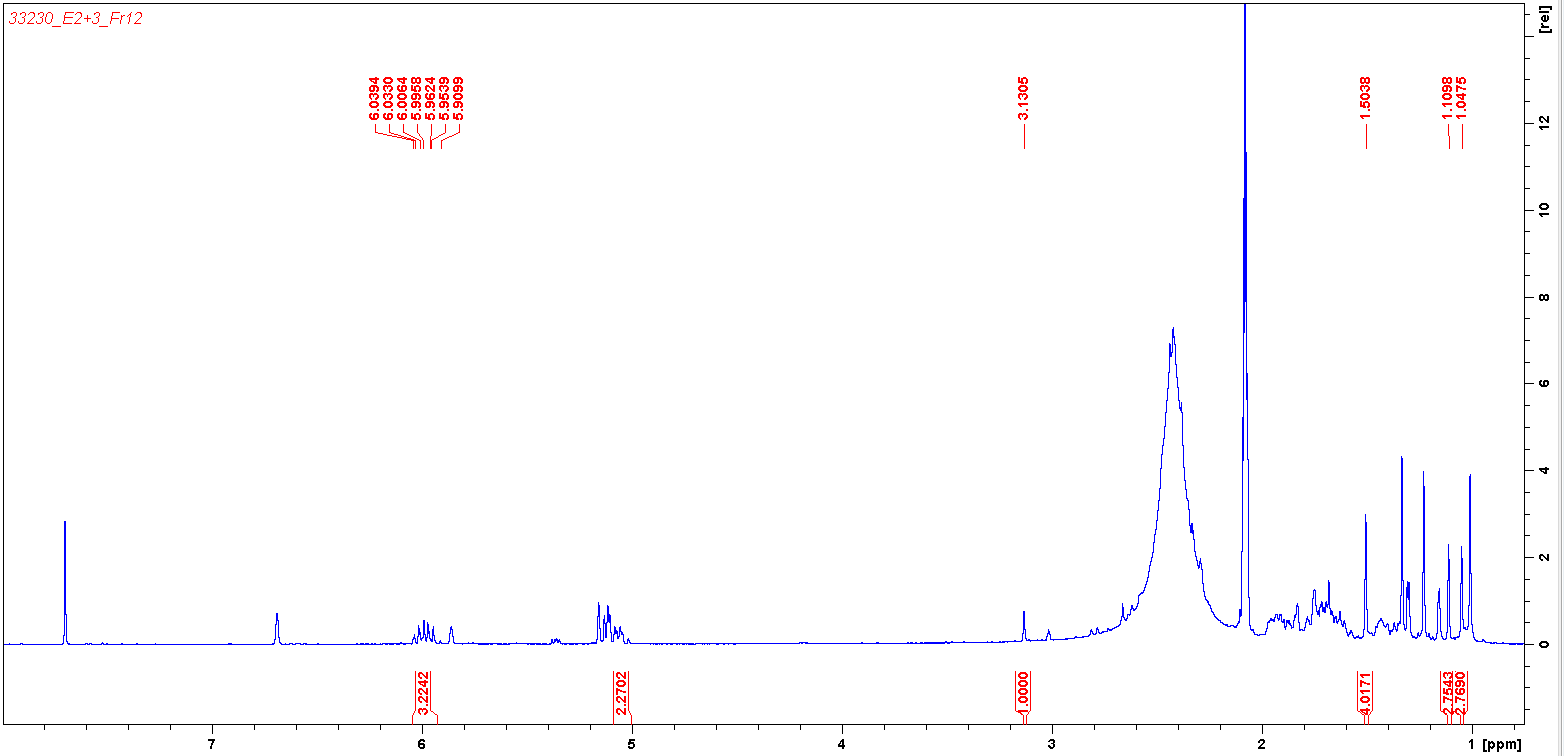


Figure S3. ^1^H NMR spectrum (400 MHz, CDCl_3_) of the mixture of **5**+**17**+**18**


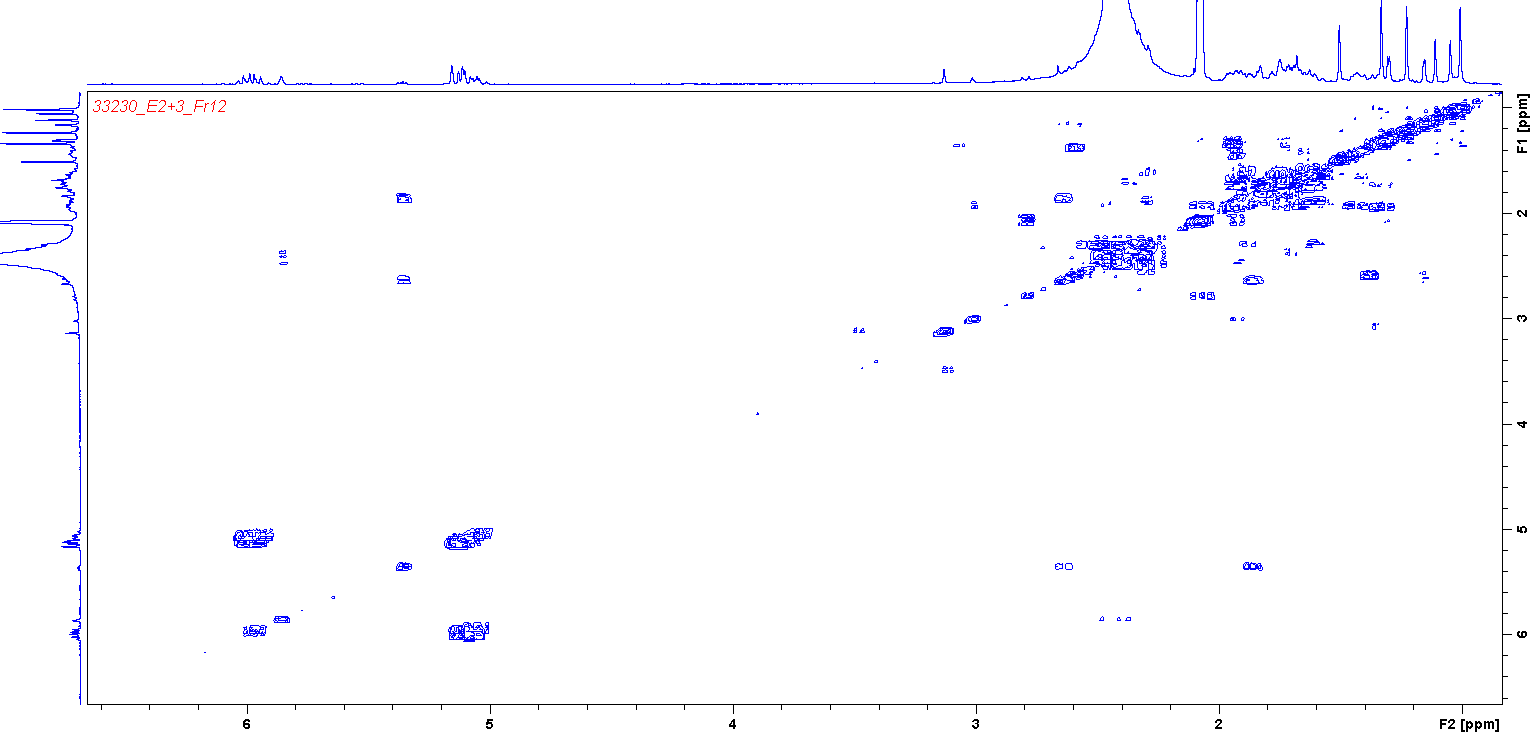


Figure S4. ^1^H,^1^H COSY spectrum of the mixture of **5**+**17**+**18**


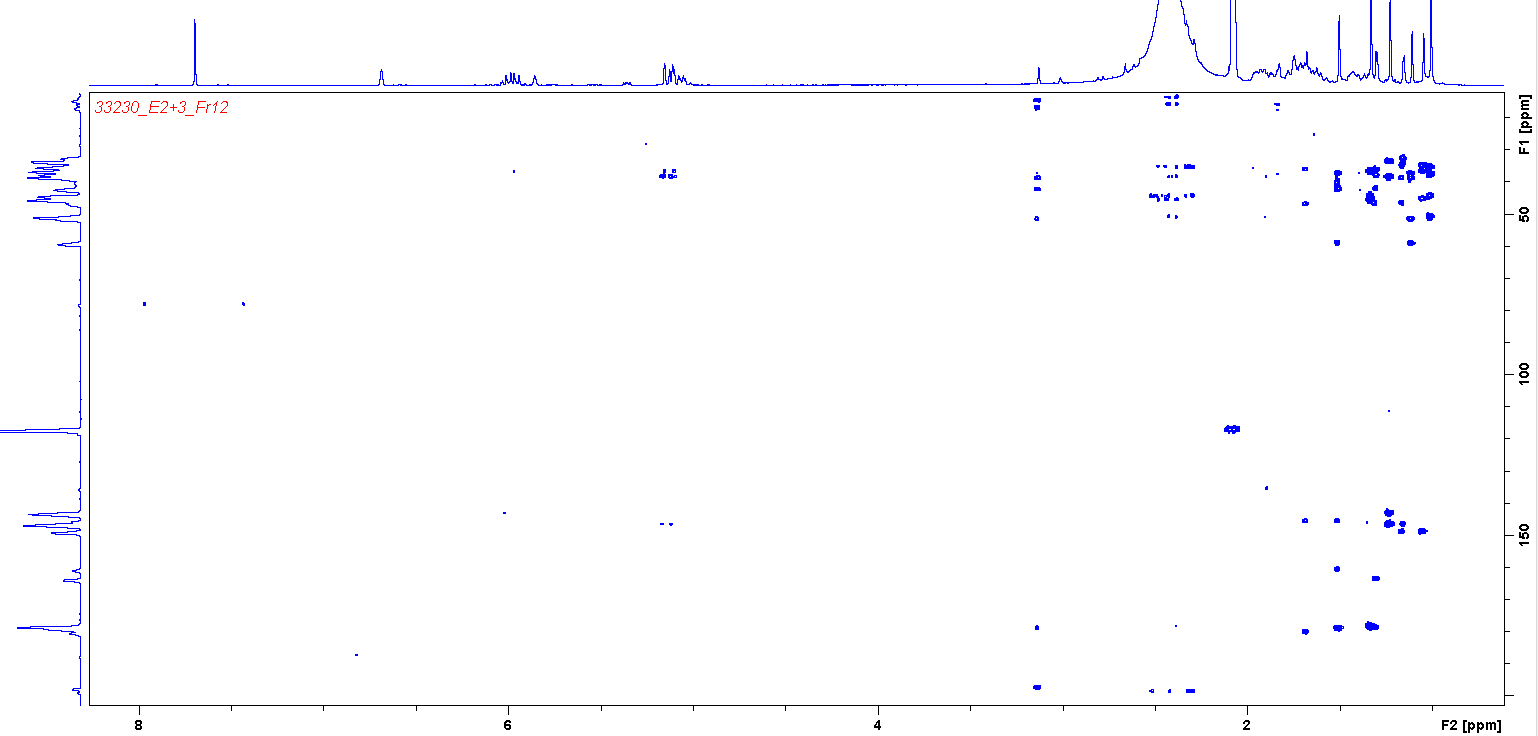


Figure S5. HMBC spectrum of the mixture of **5**+**17**+**18**


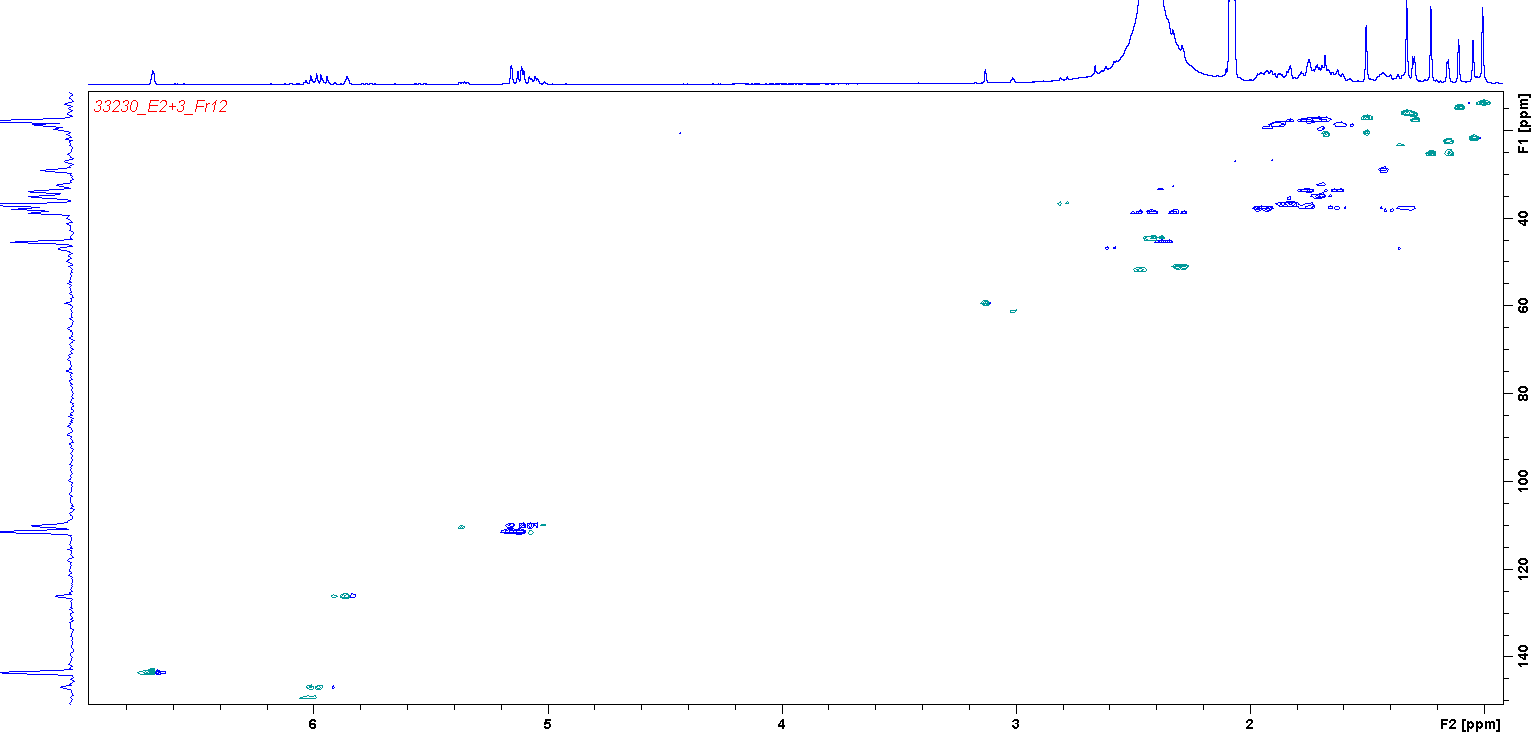


Figure S6. HSQC spectrum of the mixture of **5**+**17**+**18**


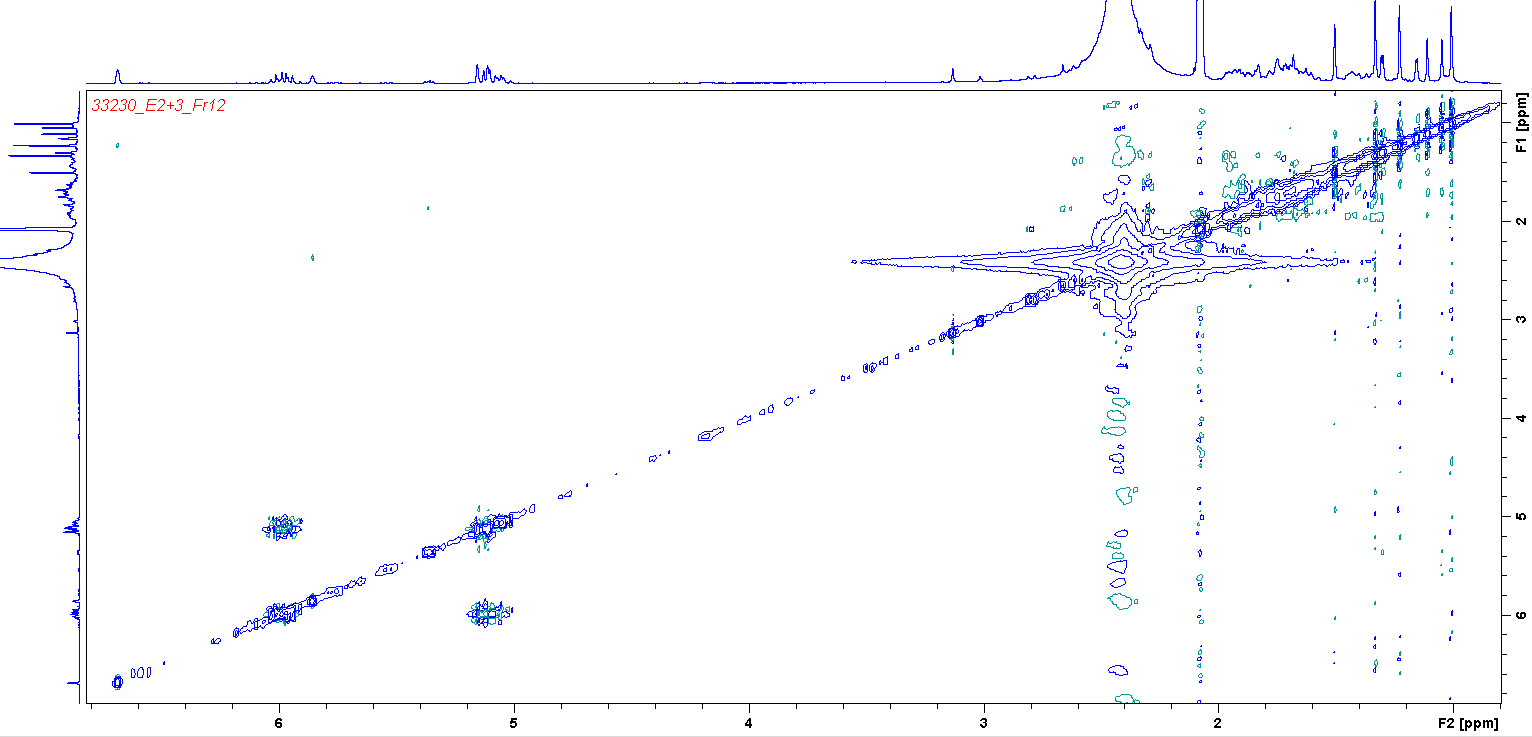


Figure S7. NOESY spectrum of the mixture of **5**+**17**+**18**


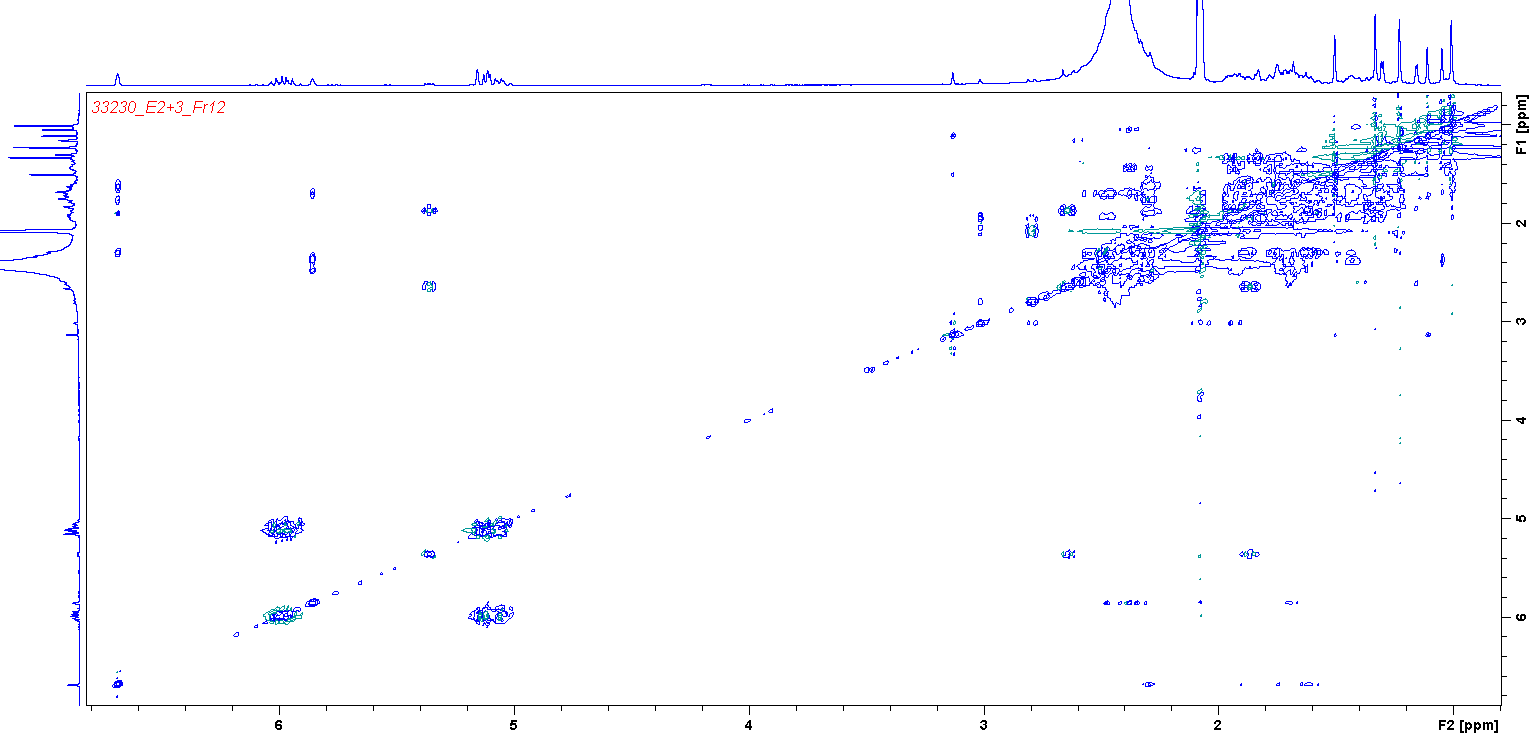


Figure S8. TOCSY spectrum of the mixture of **5**+**17**+**18**


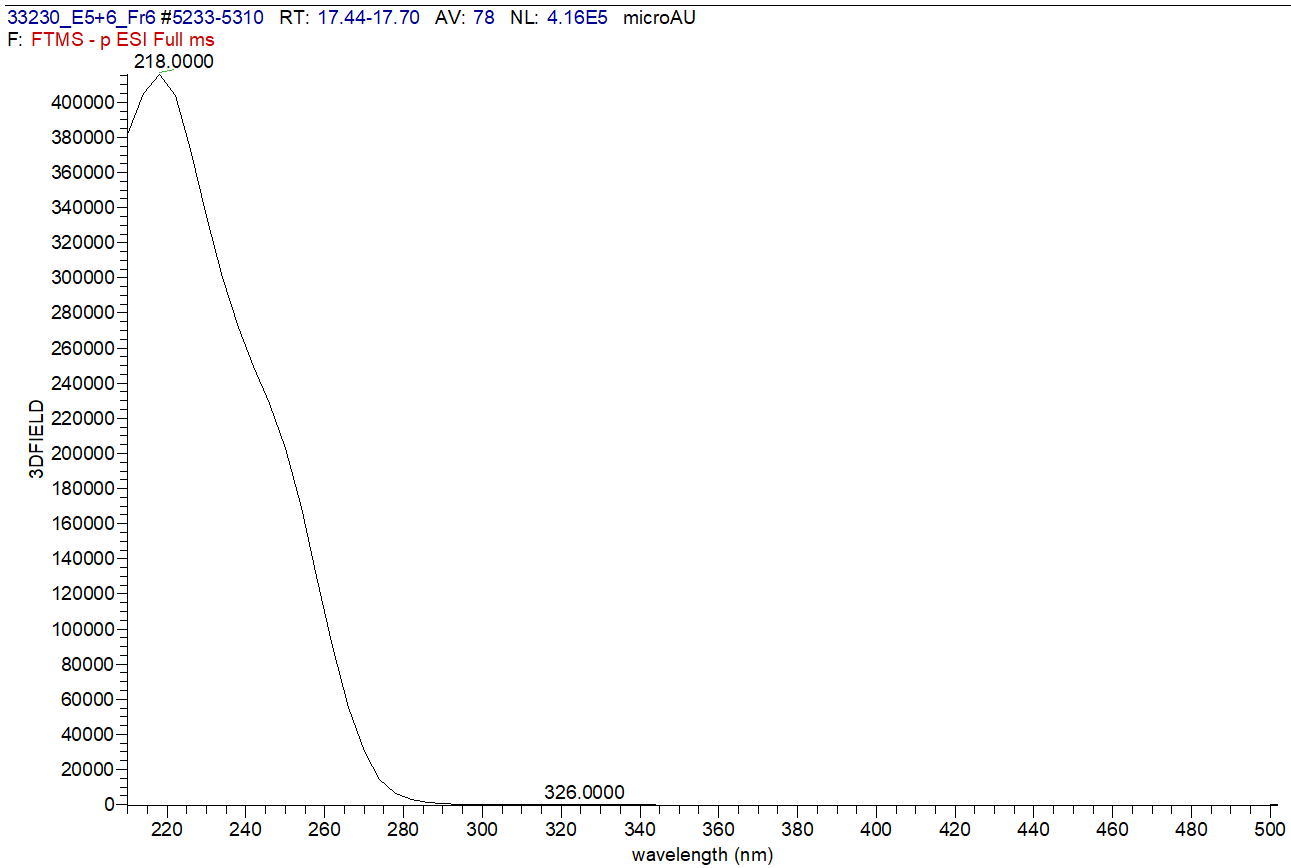


Figure S9. UV spectrum of **6**


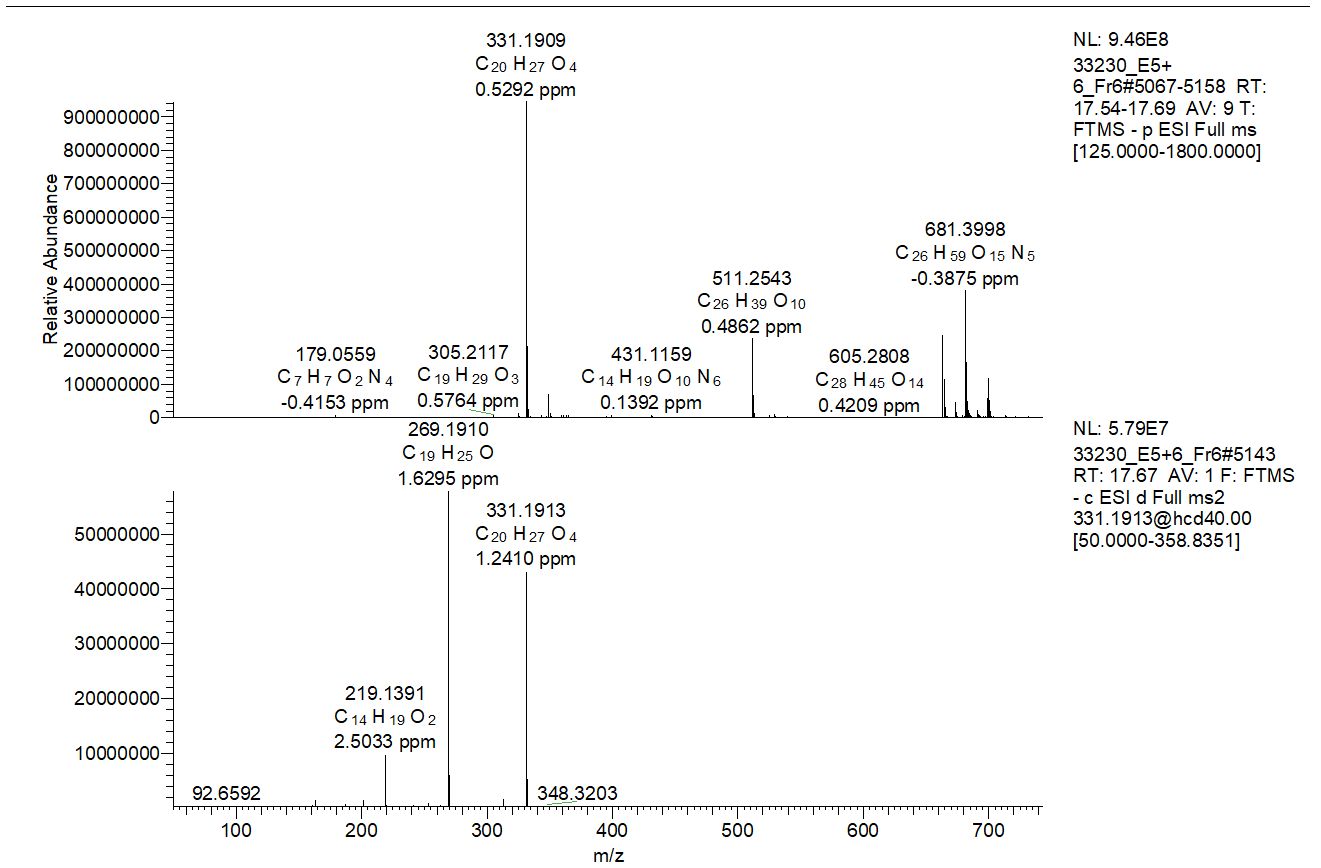


Figure S10. Full (-)-HRESI mass and MS2 spectra of **6**


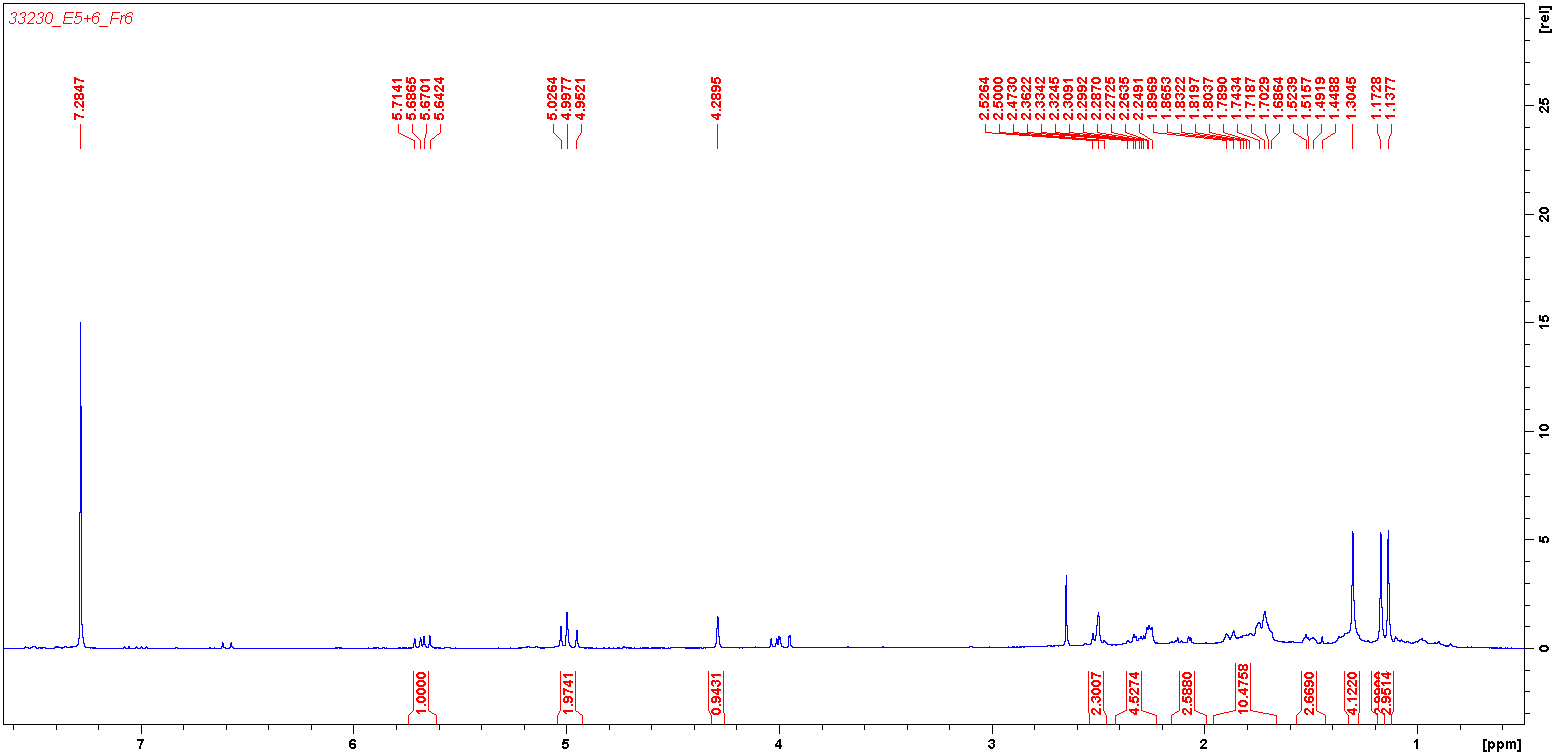


Figure S11. ^1^H NMR spectrum (400 MHz, CDCl_3_) of **6**


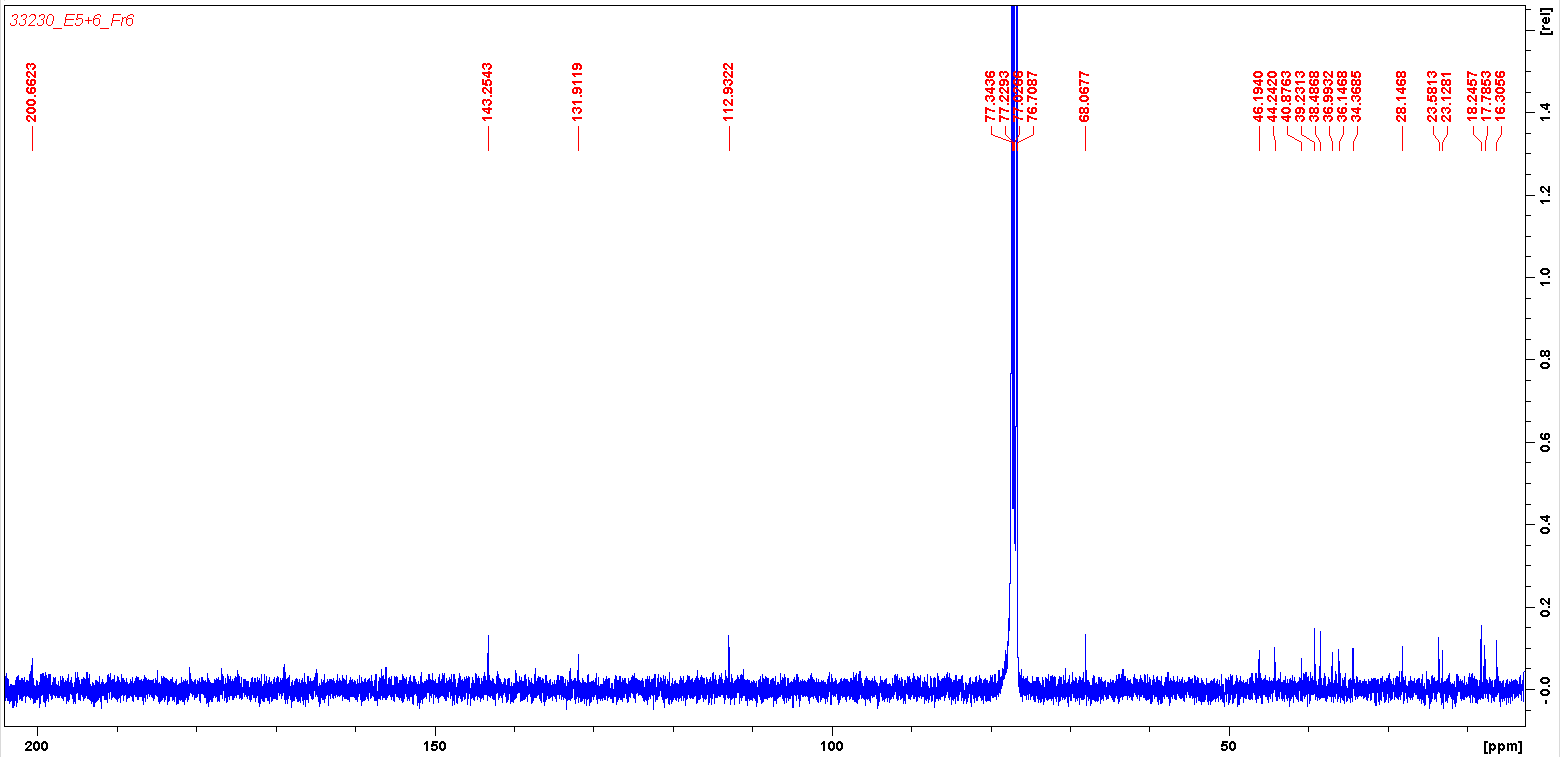


Figure S12. ^13^C NMR spectrum (100 MHz, CDCl_3_) of **6**


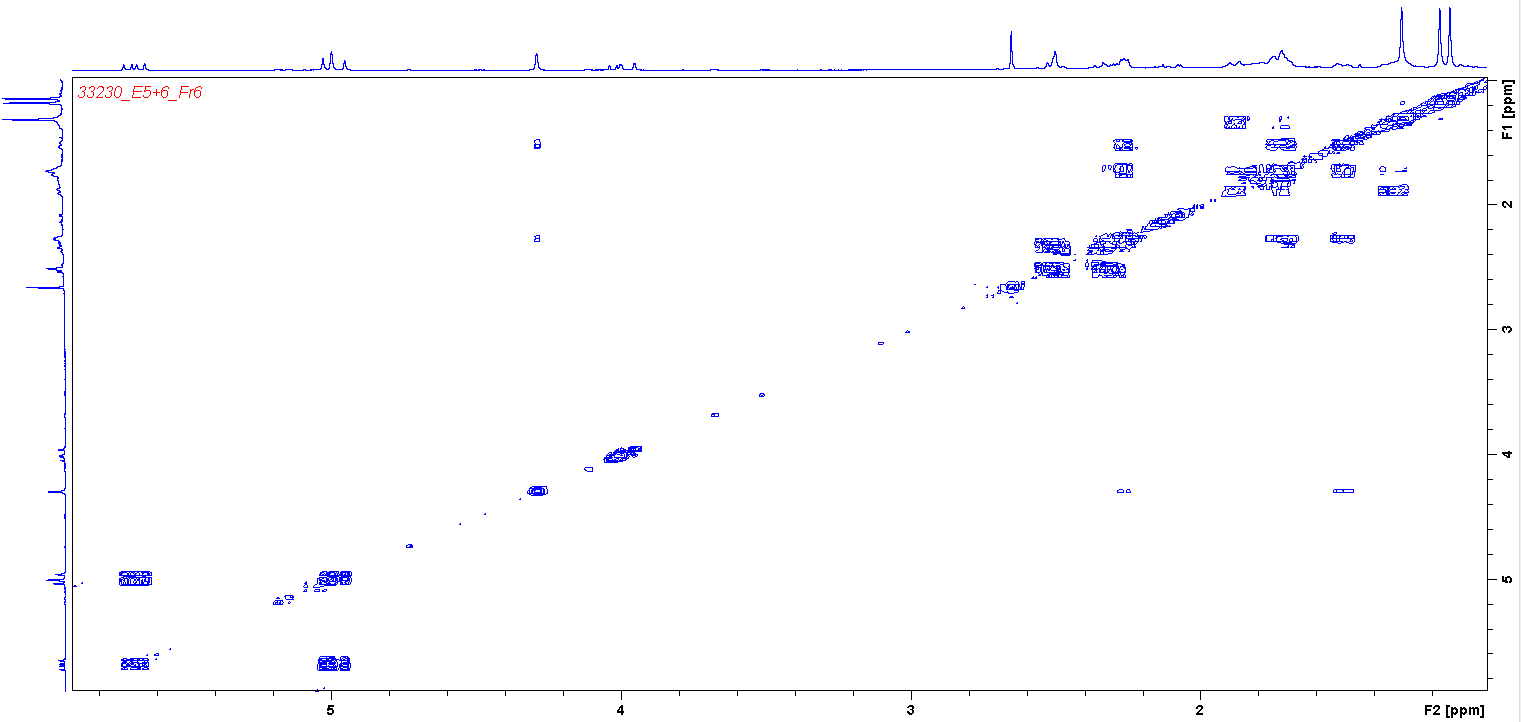


Figure S13. ^1^H,^1^H COSY spectrum of **6**


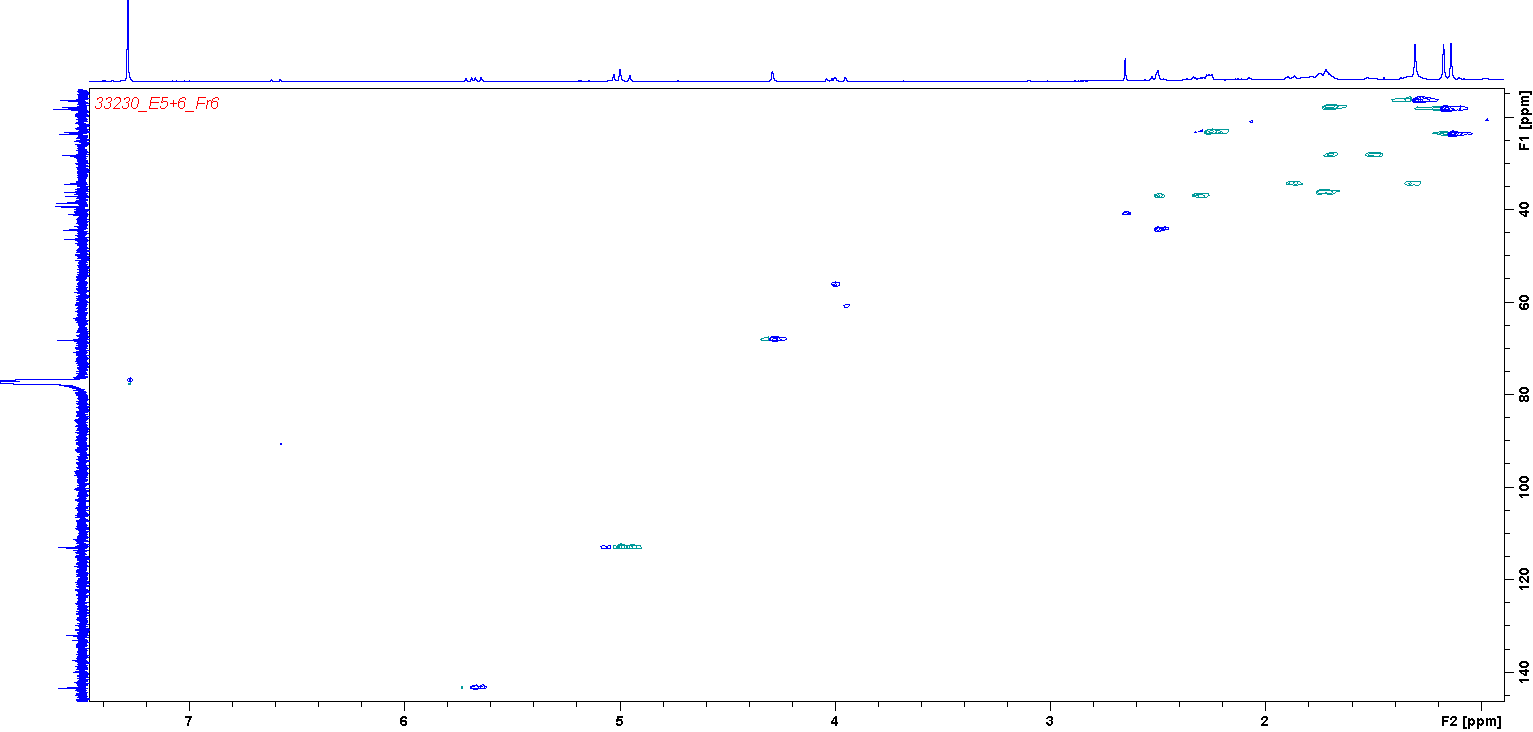


Figure S14. HSQC spectrum of **6**


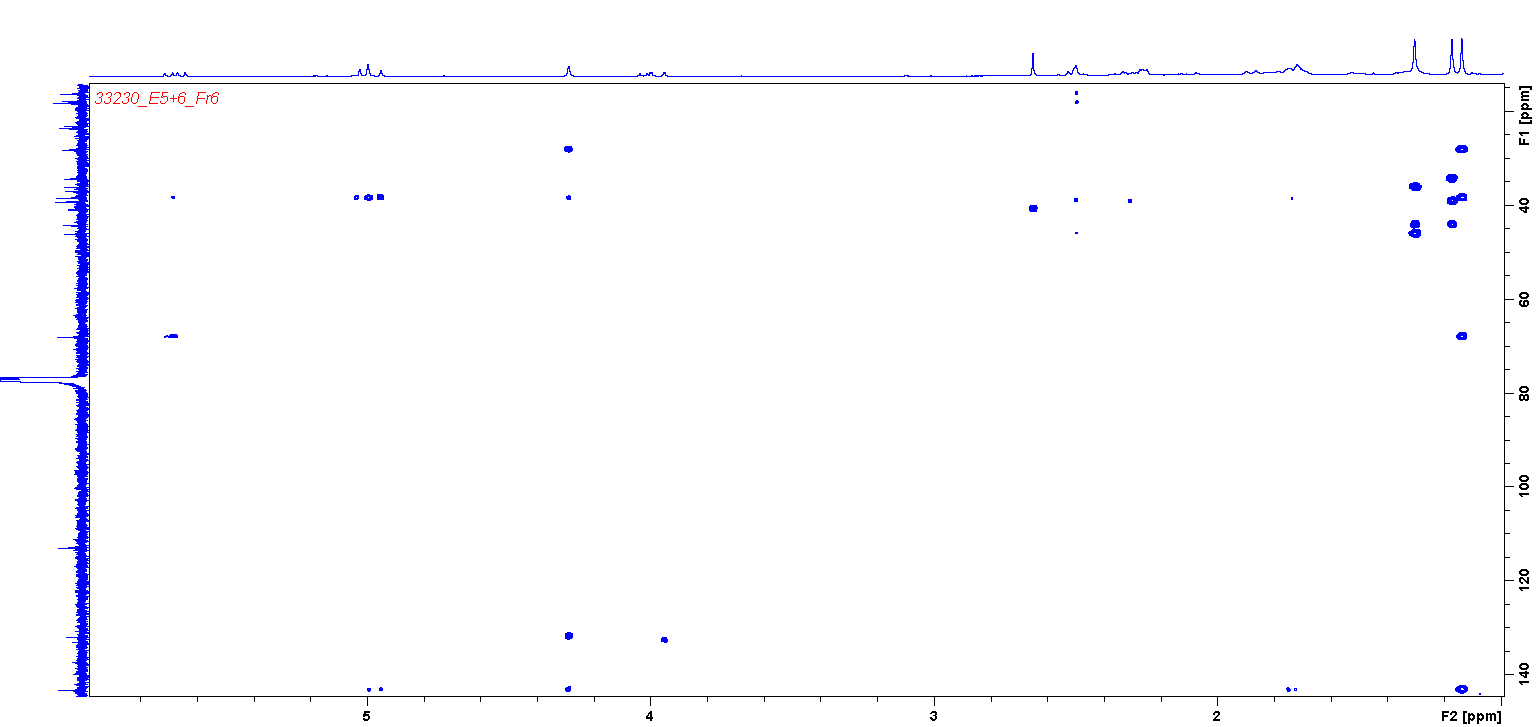


Figure S15. HMBC spectrum of **6**


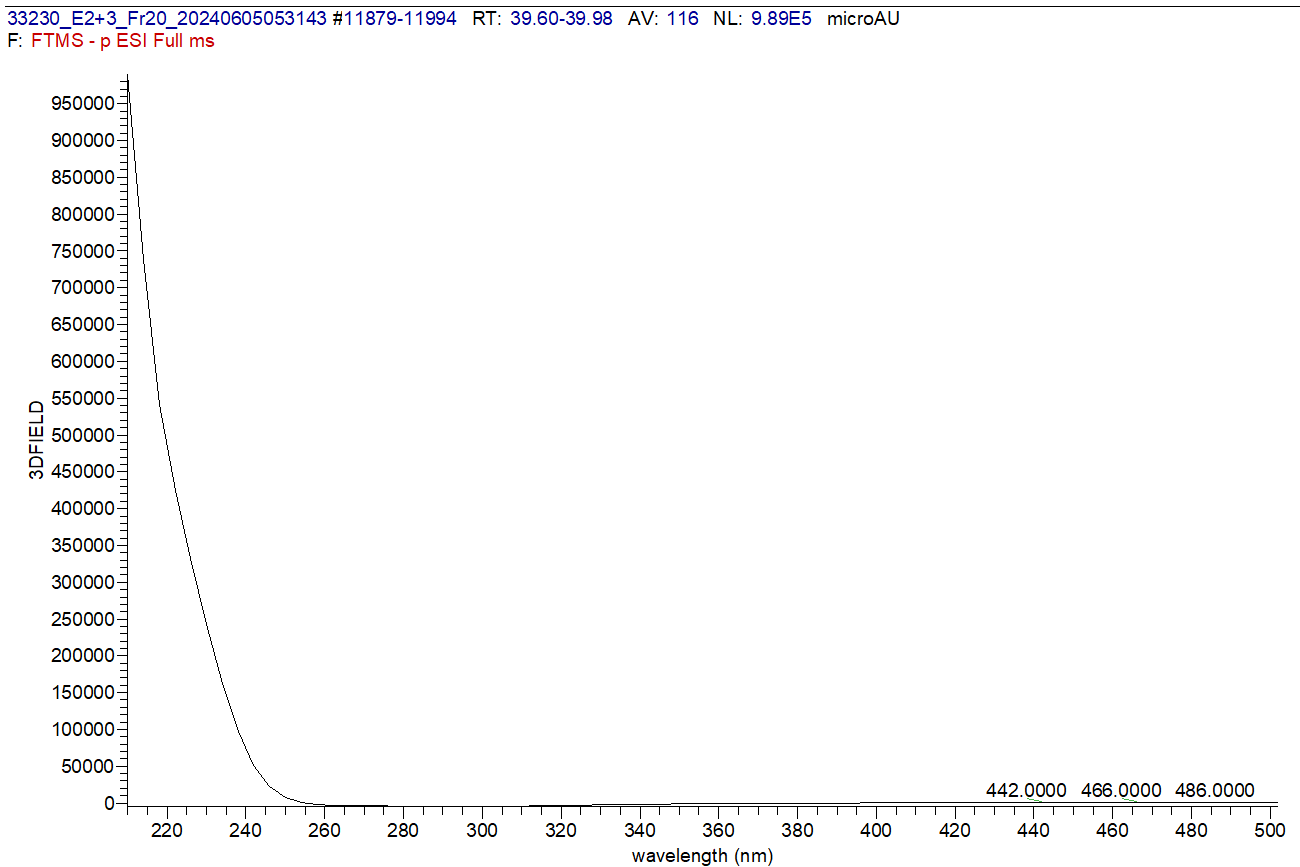


Figure S16. UV spectrum of **7**


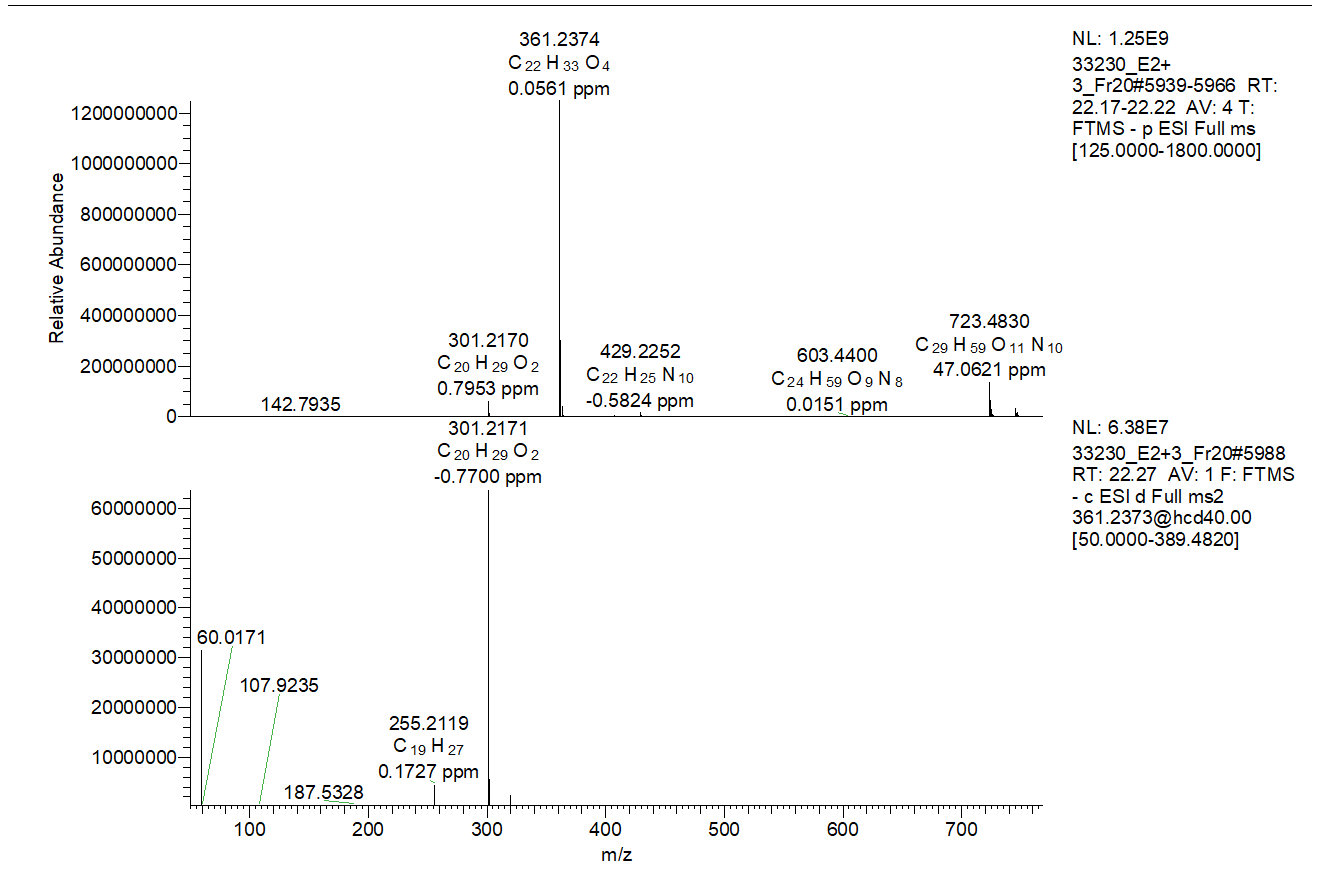


Figure S17. Full (-)-HRESI mass and MS2 spectra of **7**


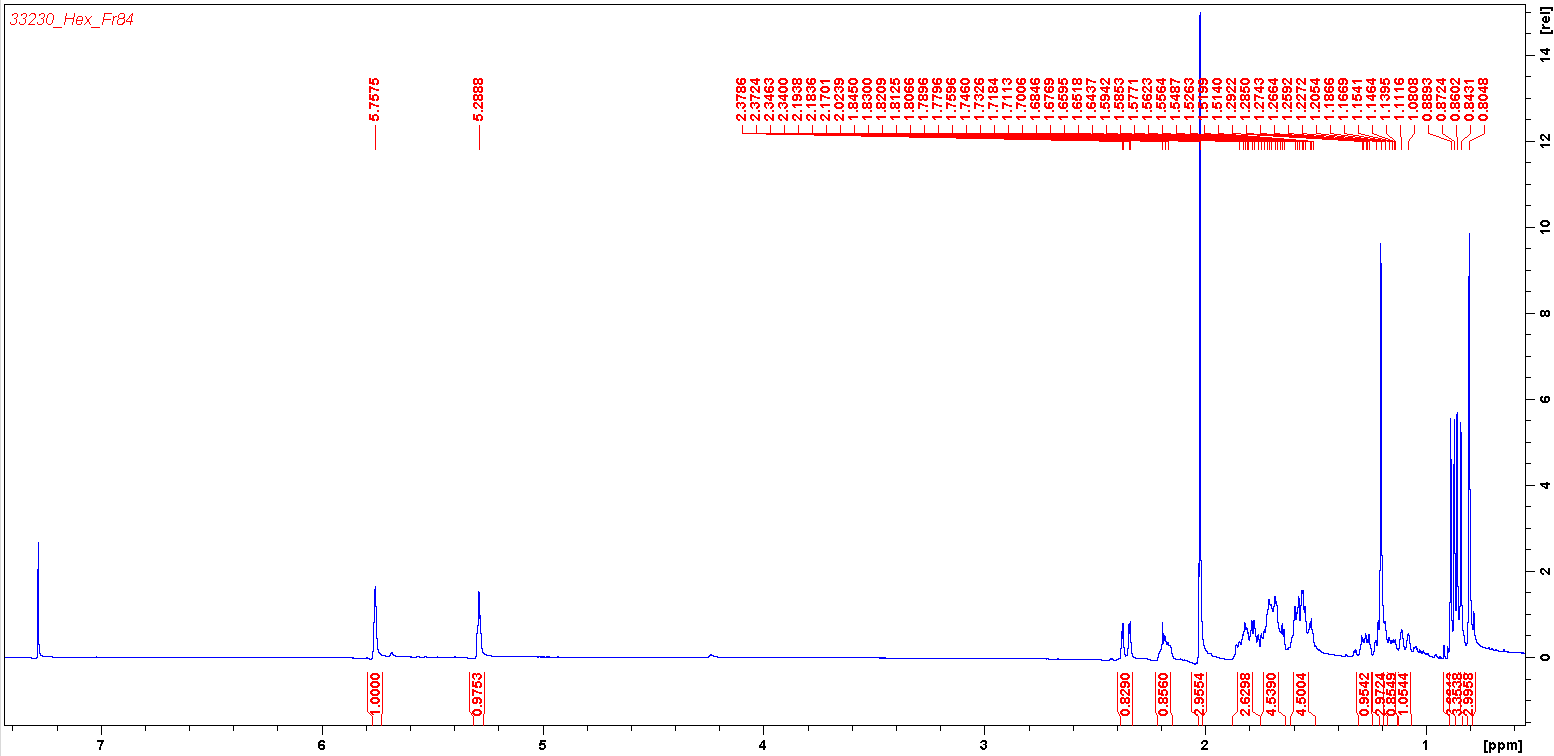


Figure S18. ^1^H NMR spectrum (400 MHz, CDCl_3_) of **7**


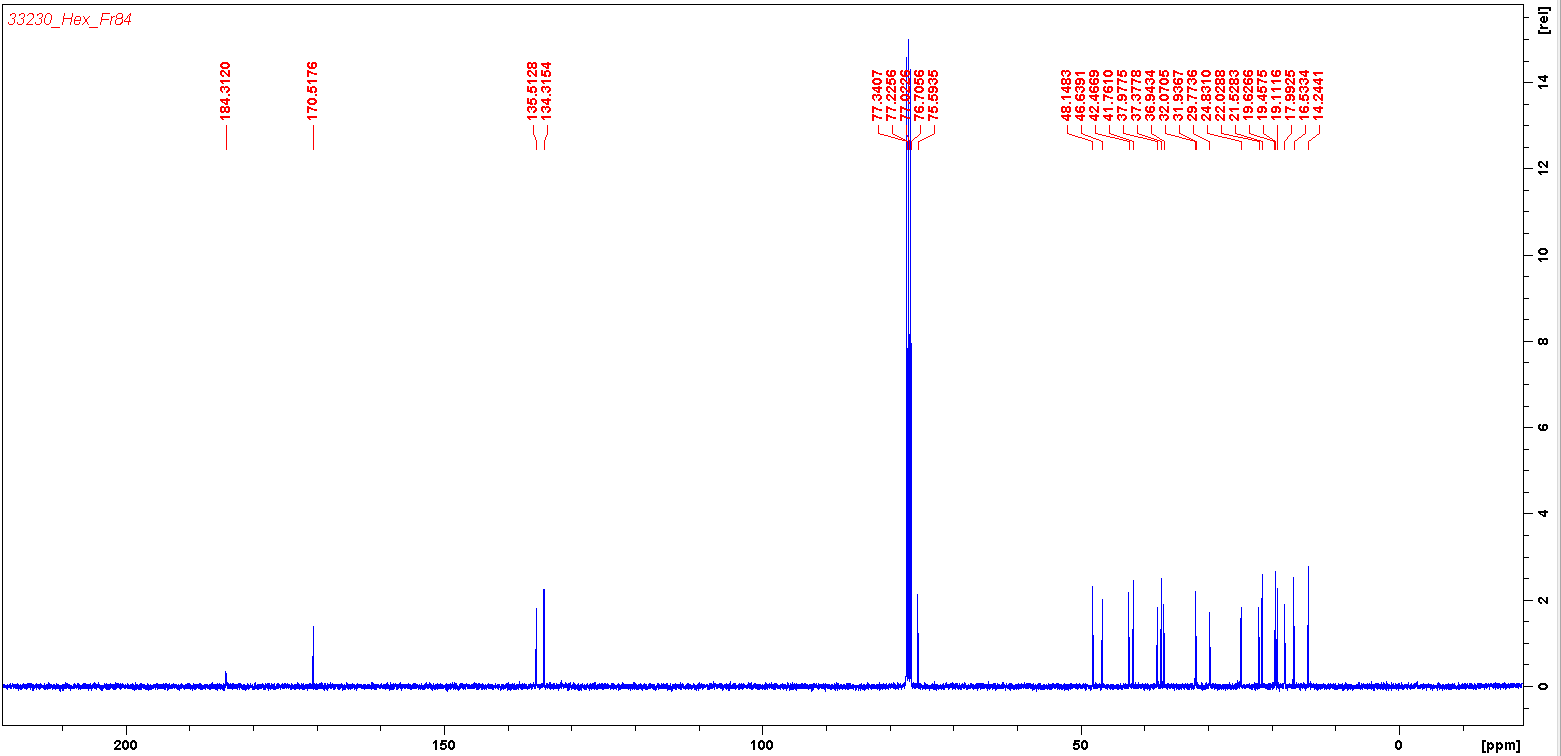


Figure S19. ^13^C NMR spectrum (100 MHz, CDCl_3_) of **7**


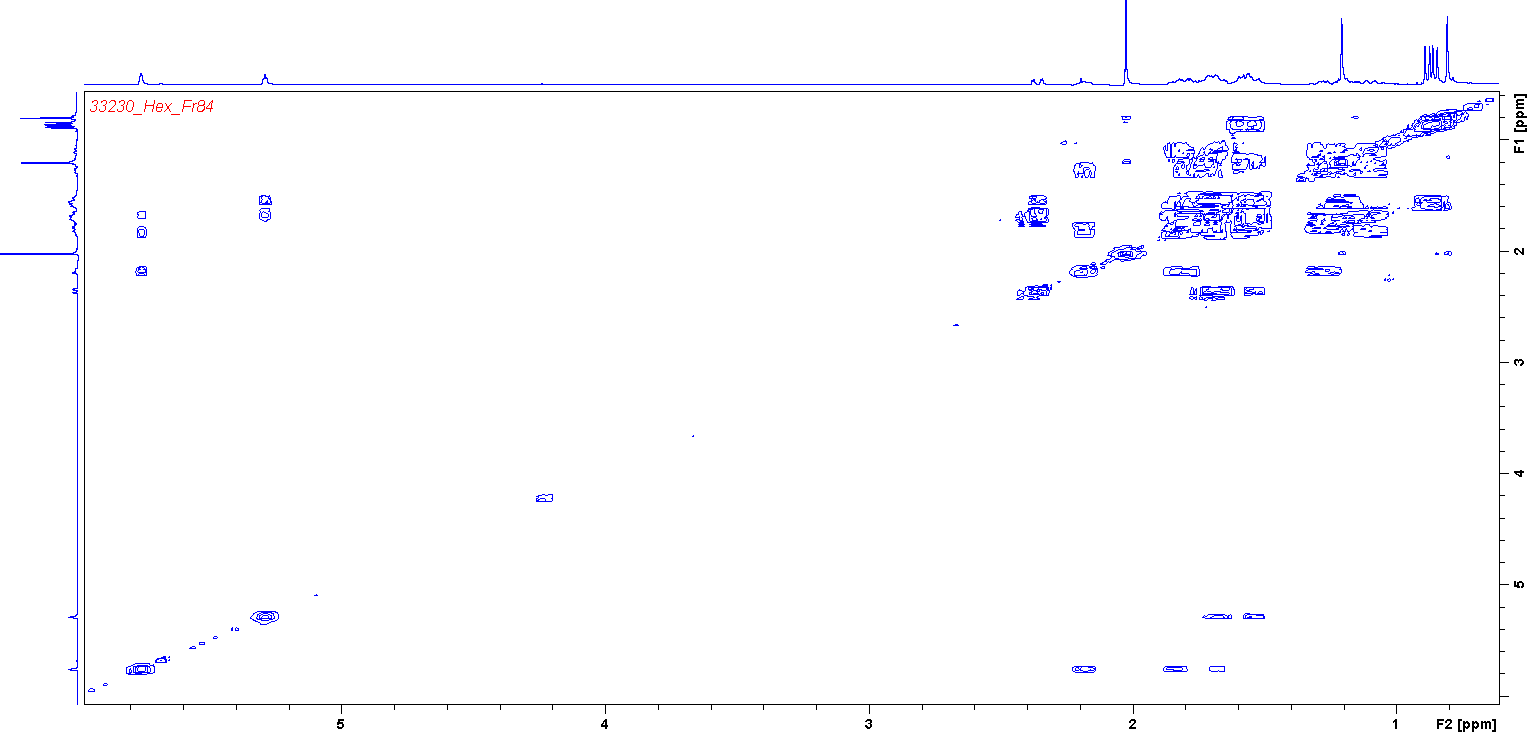


Figure S20. ^1^H,^1^H COSY spectrum of **7**


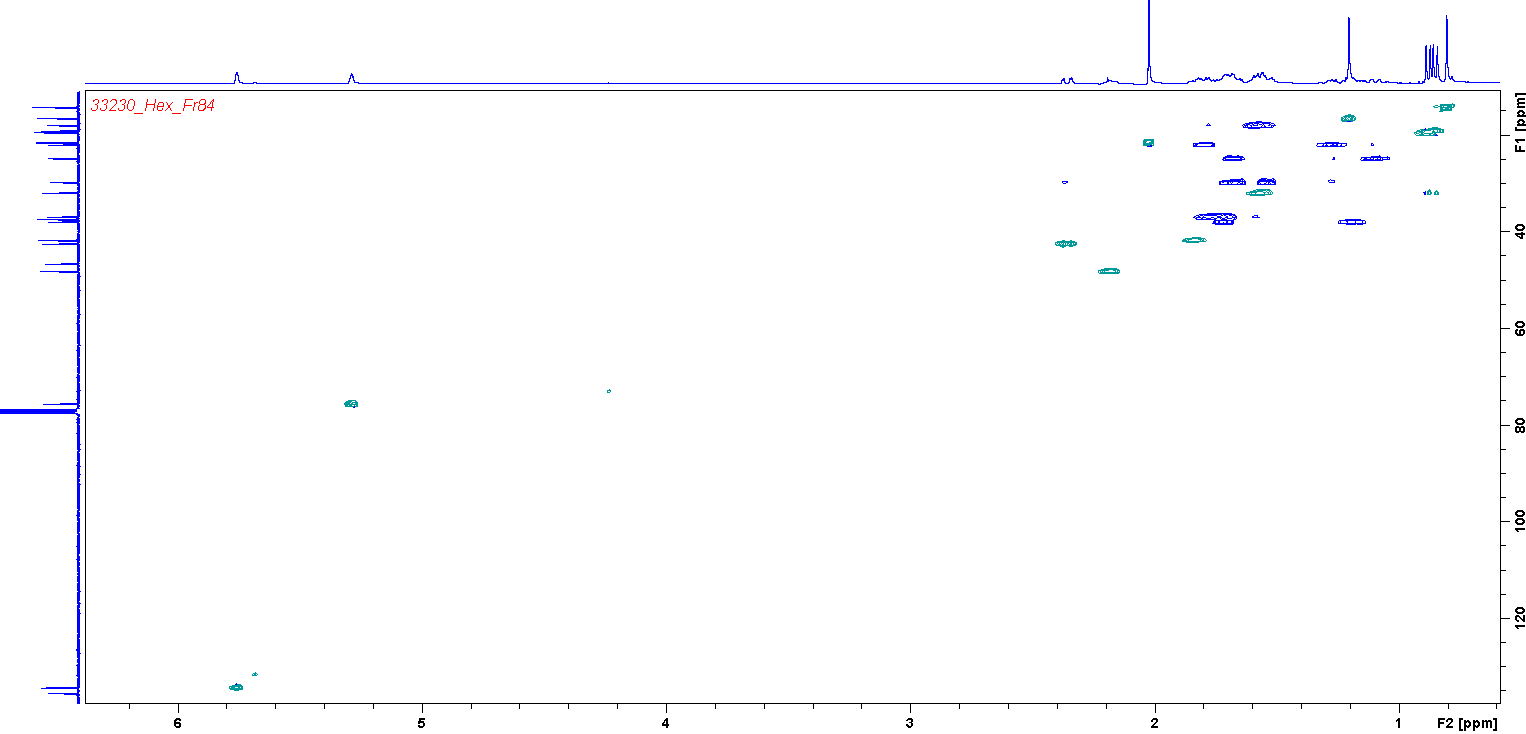


Figure S21. HSQC spectrum of **7**


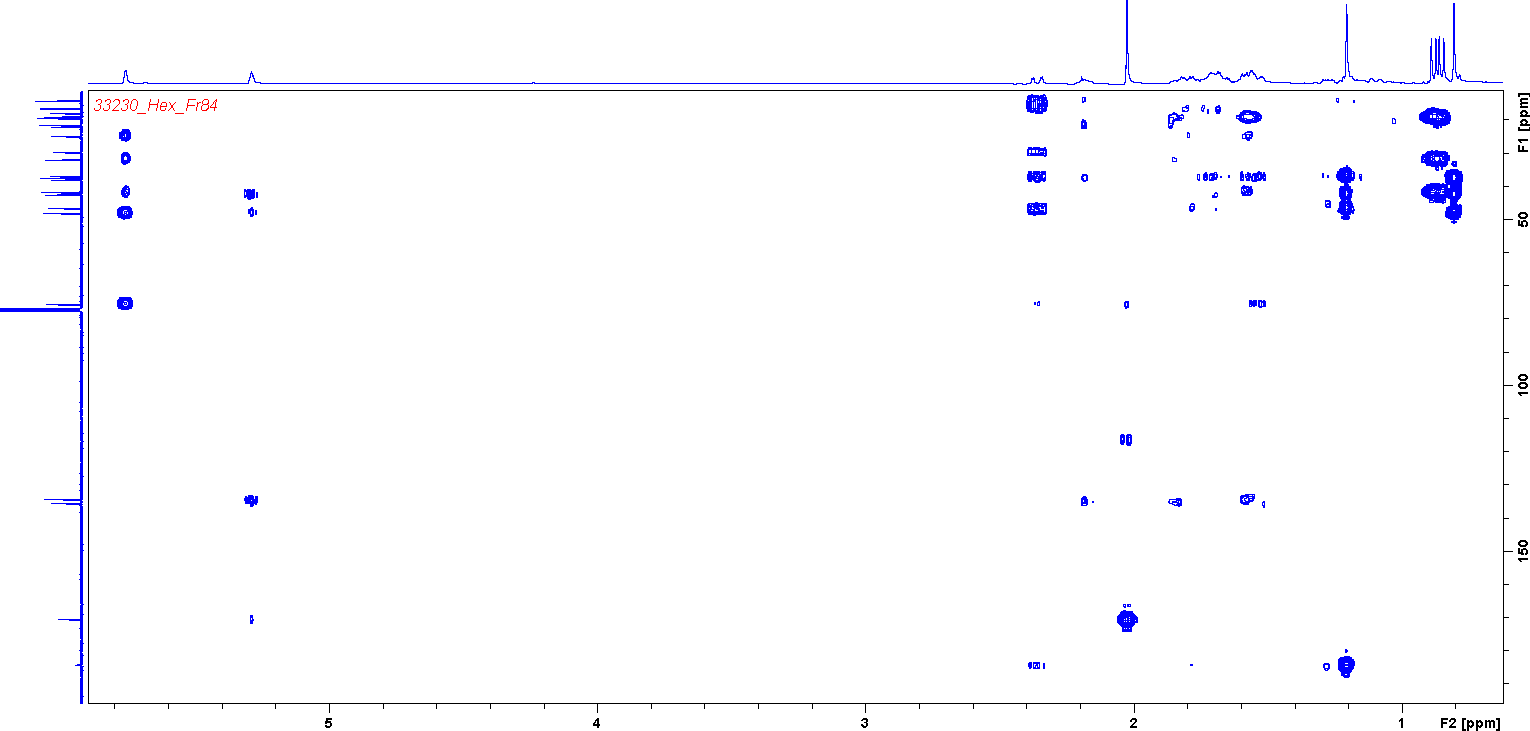


Figure S22. HMBC spectrum of **7**


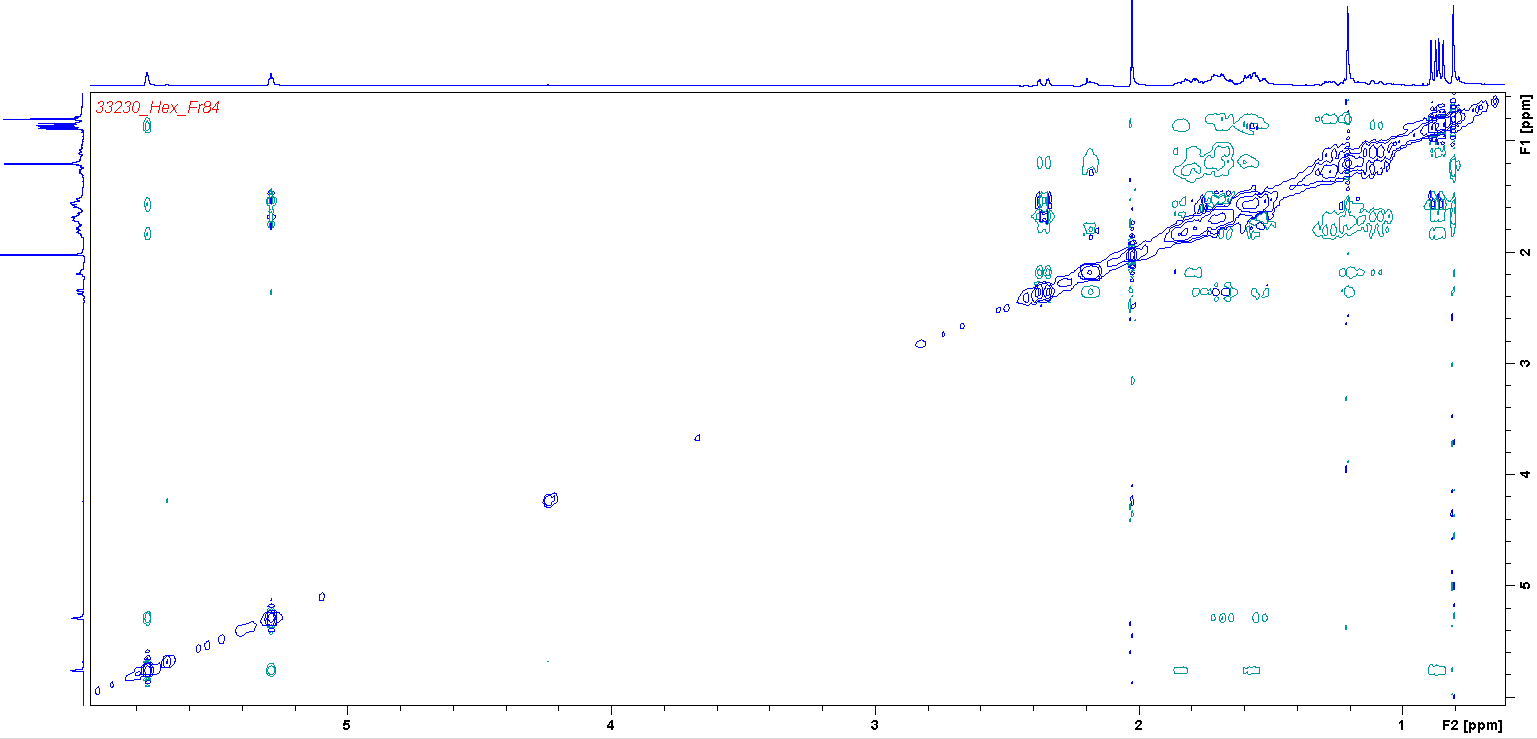


Figure S23. NOESY spectrum of **7**


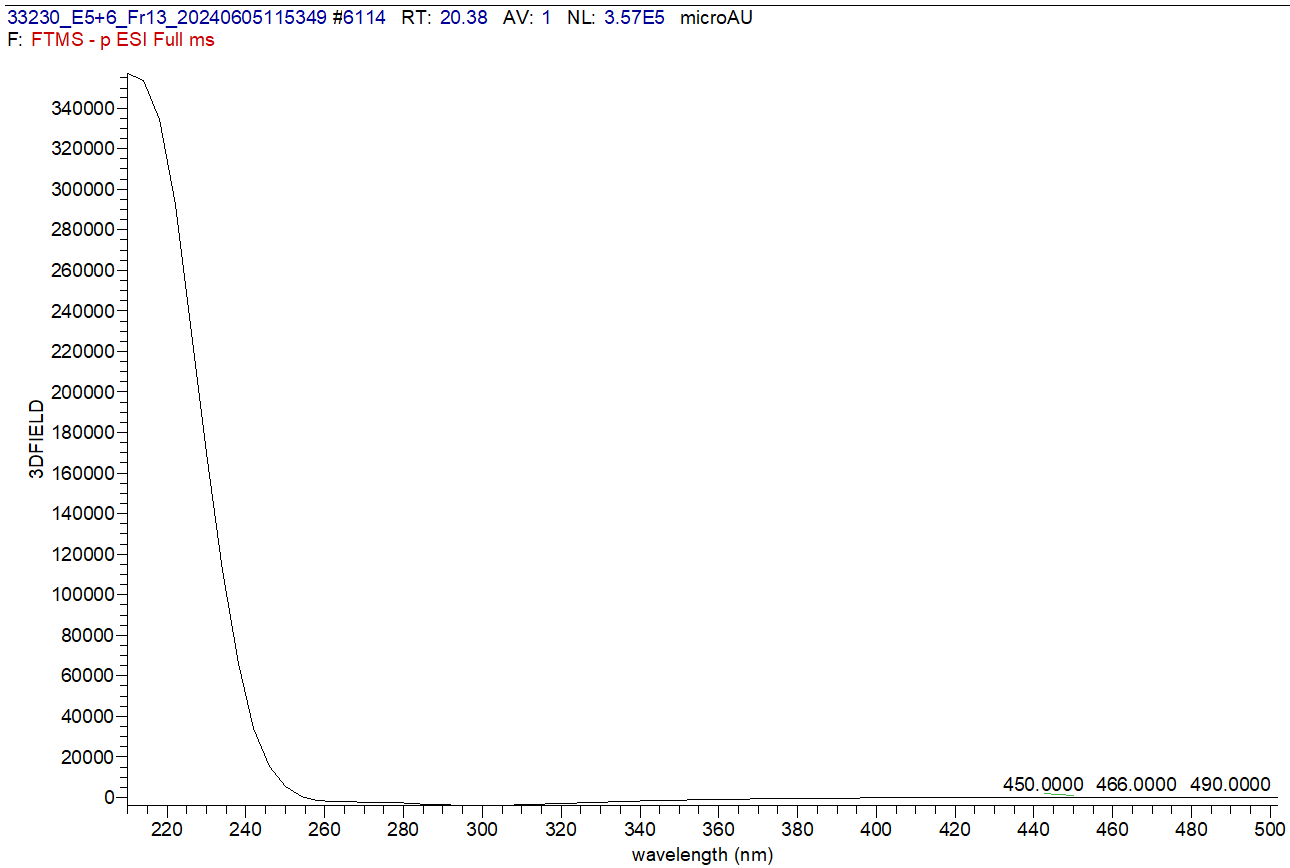


Figure S24. UV spectrum of the mixture of **8**+**11**


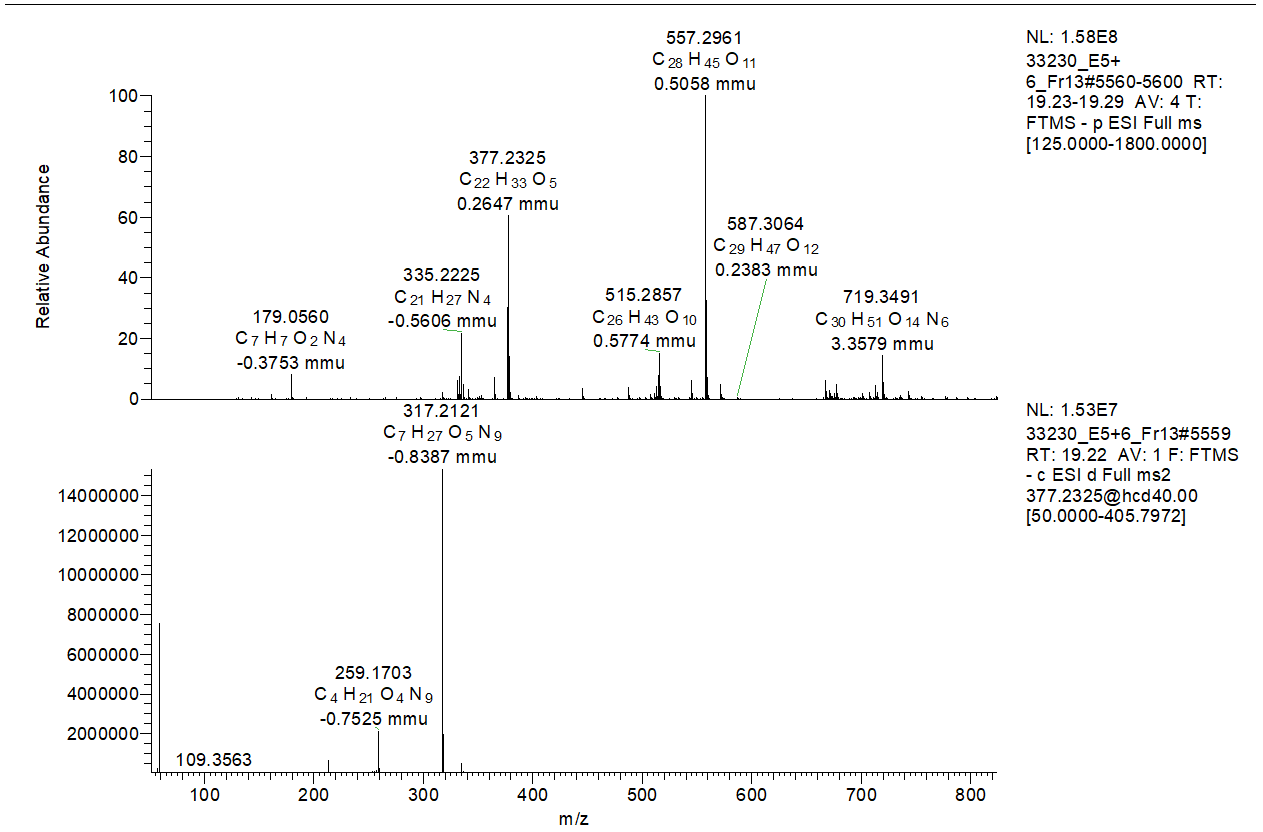


Figure S25. Full (-)-HRESI mass and MS2 spectra of **8**


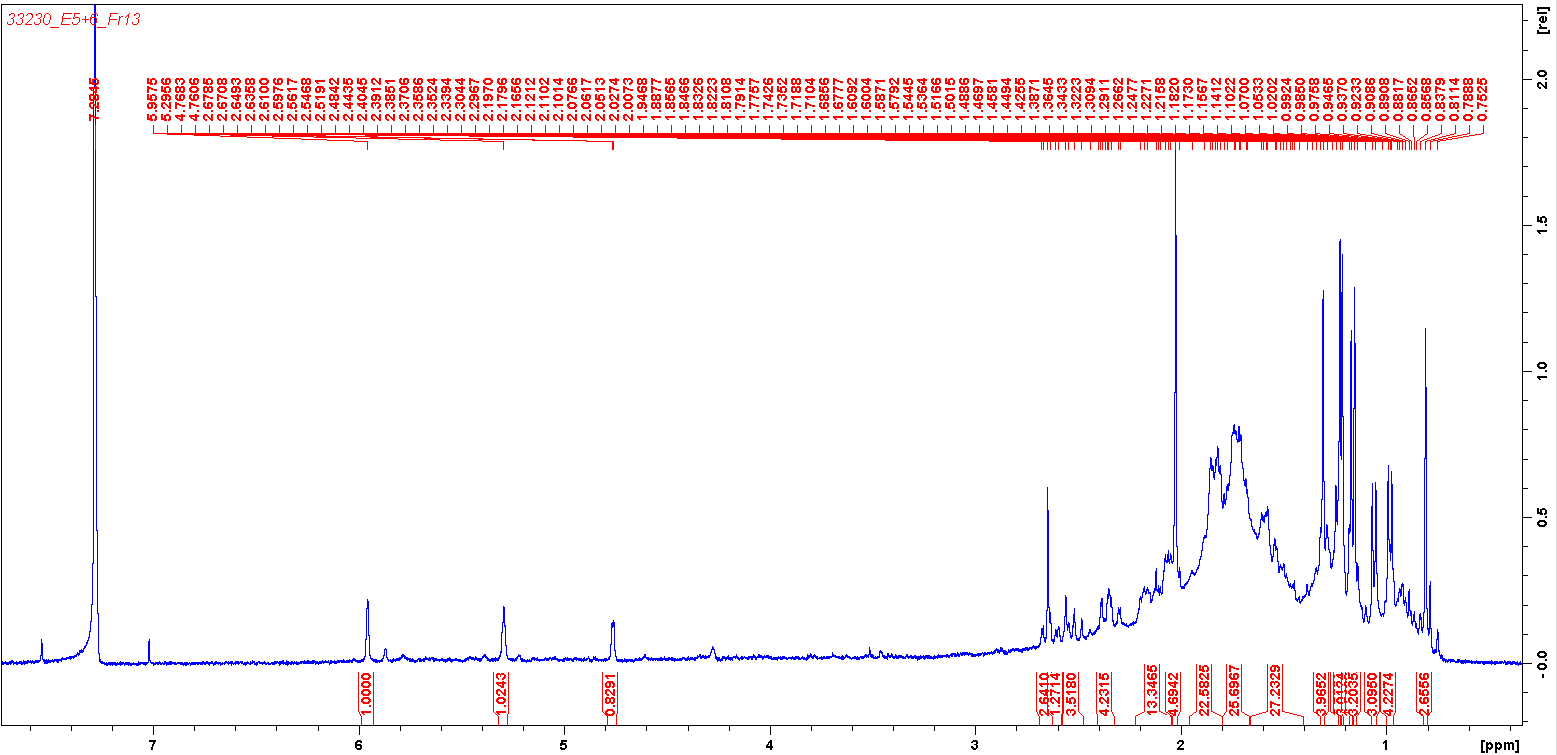


Figure S26. ^1^H NMR spectrum (400 MHz, CDCl_3_) of the mixture of **8**+**11**


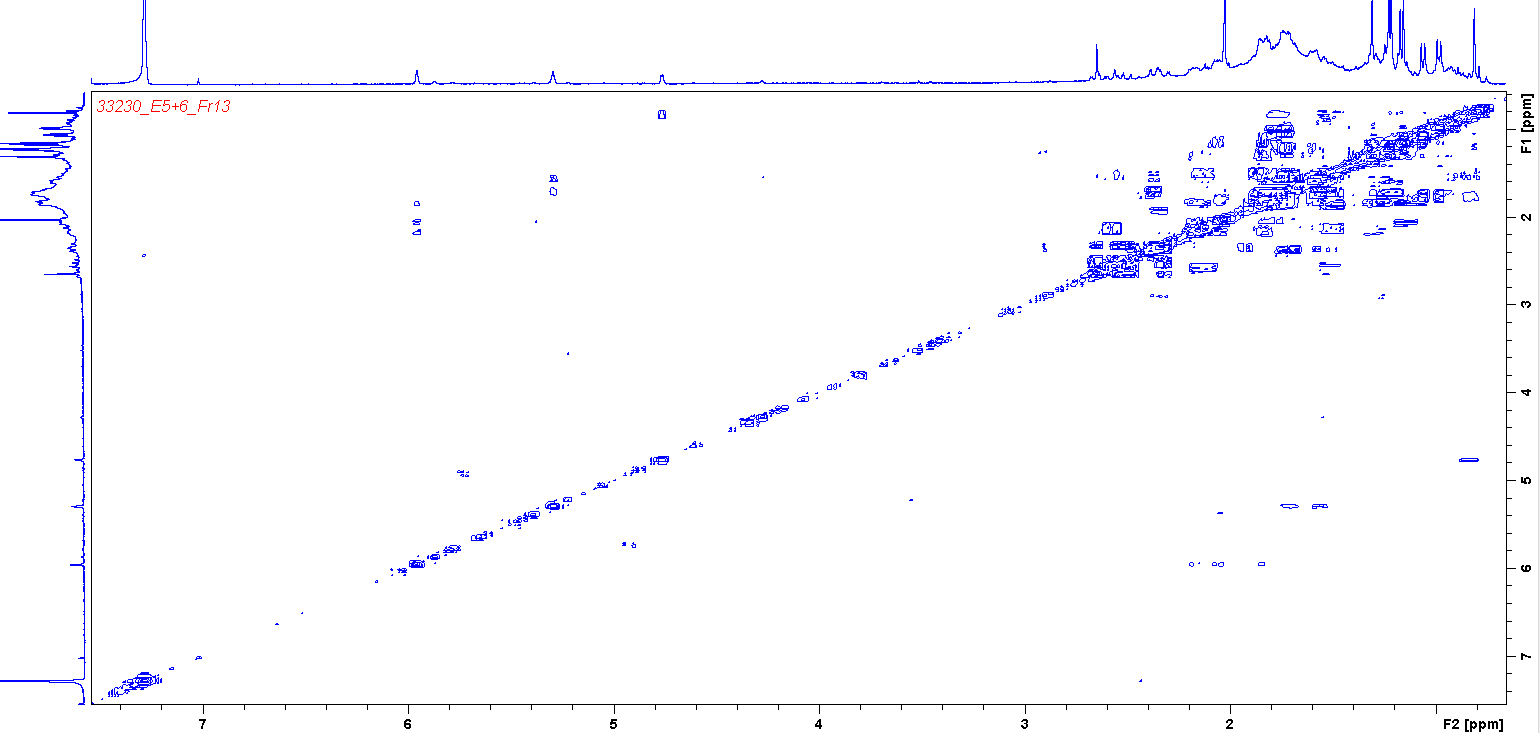


Figure S27. ^1^H,^1^H COSY spectrum of the mixture of **8**+**11**


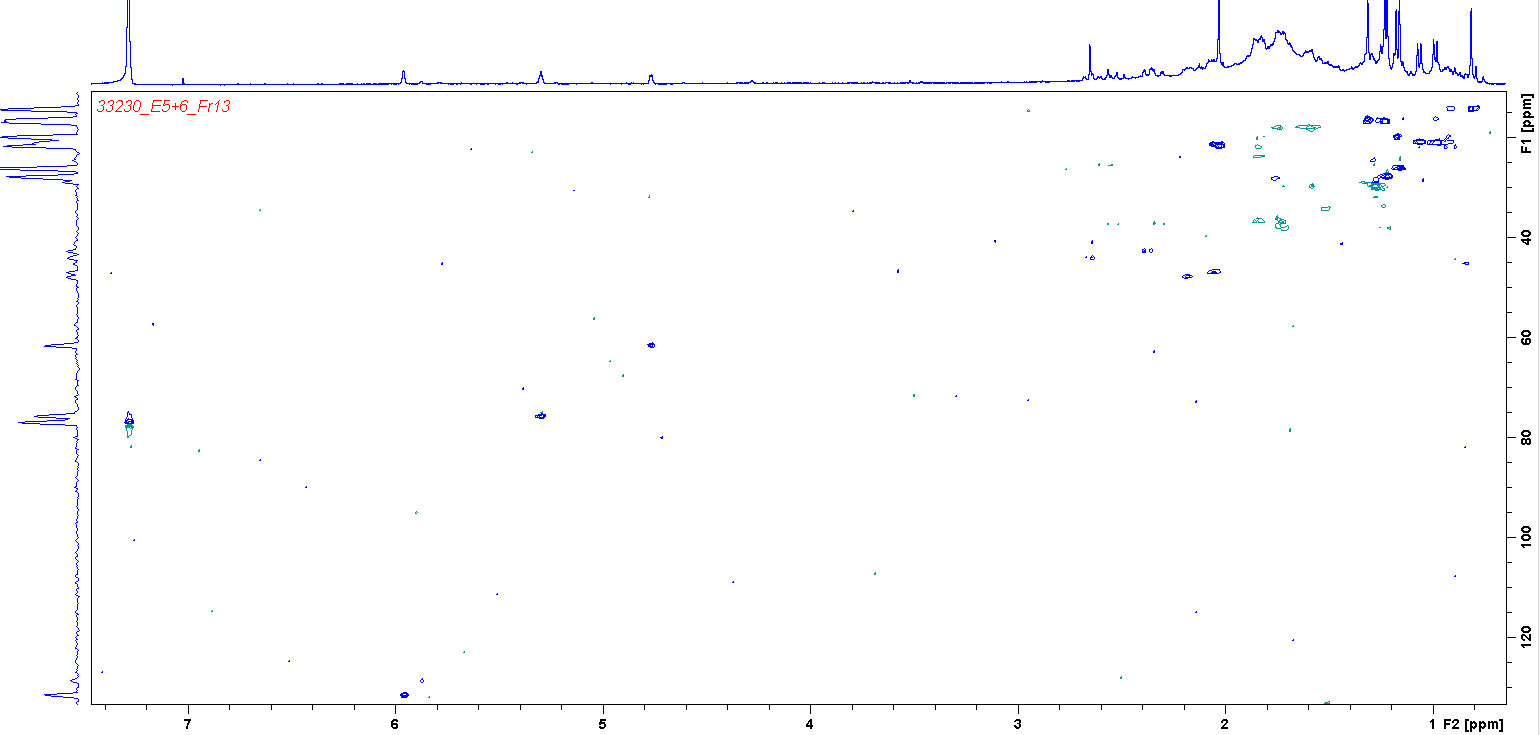


Figure S28. HSQC spectrum of the mixture of **8**+**11**


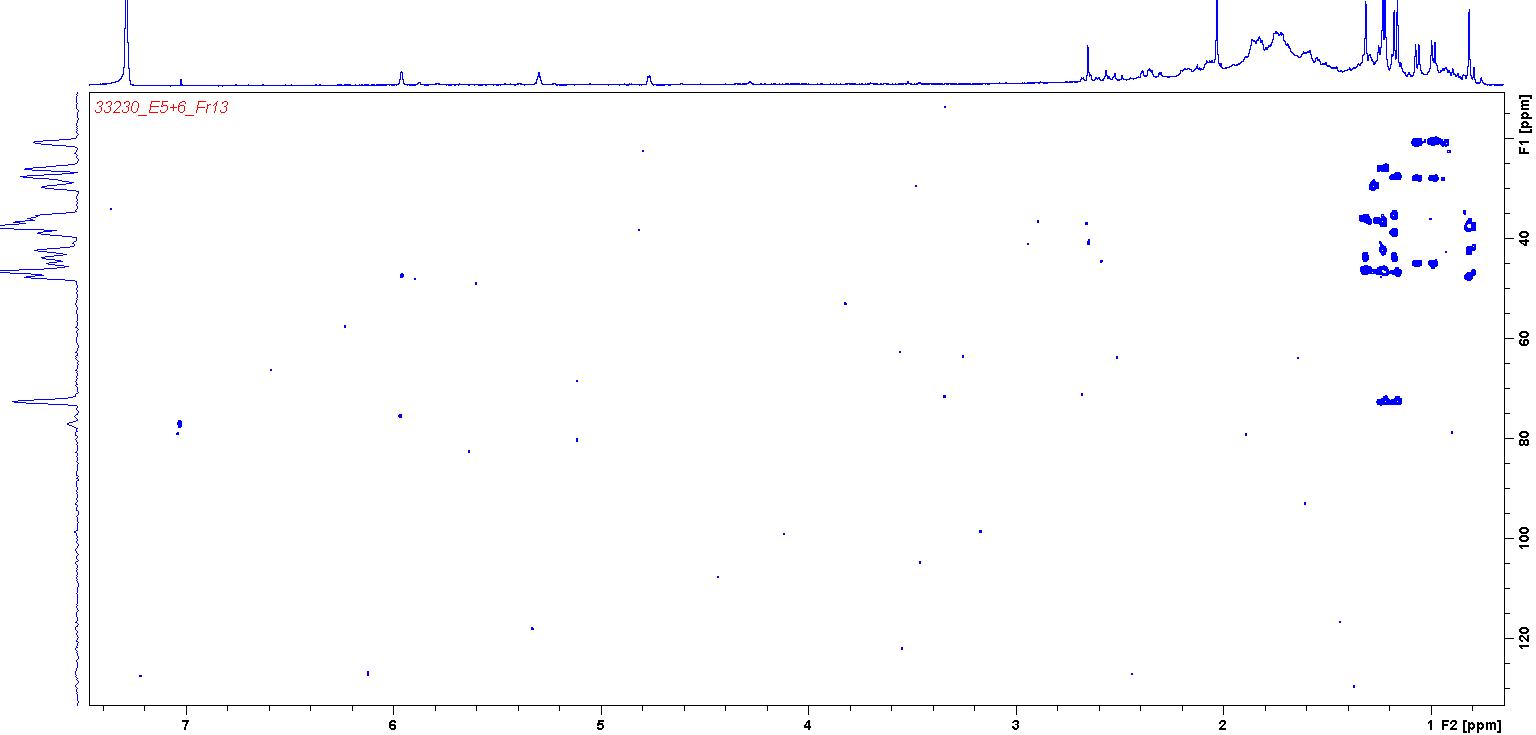


Figure S29. HMBC spectrum of the mixture of **8**+**11**


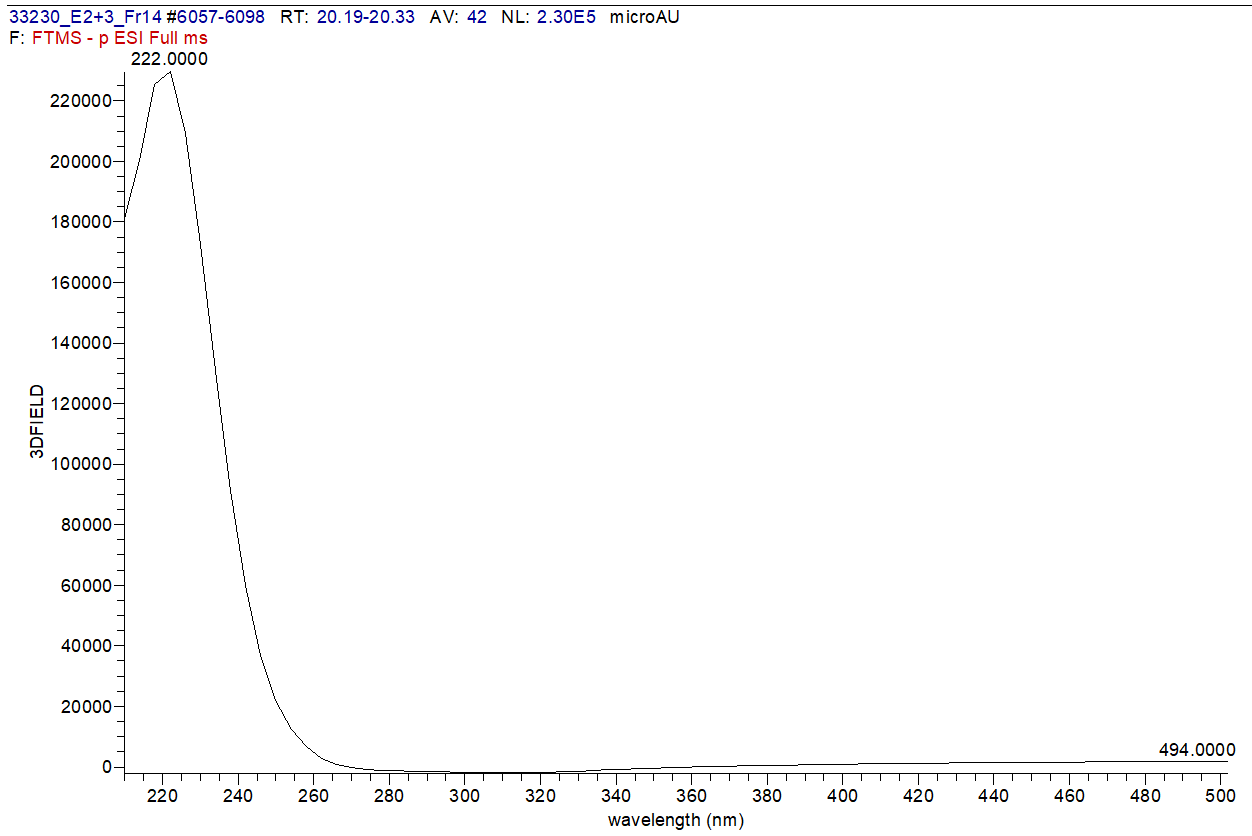


Figure S30. UV spectrum of **9**


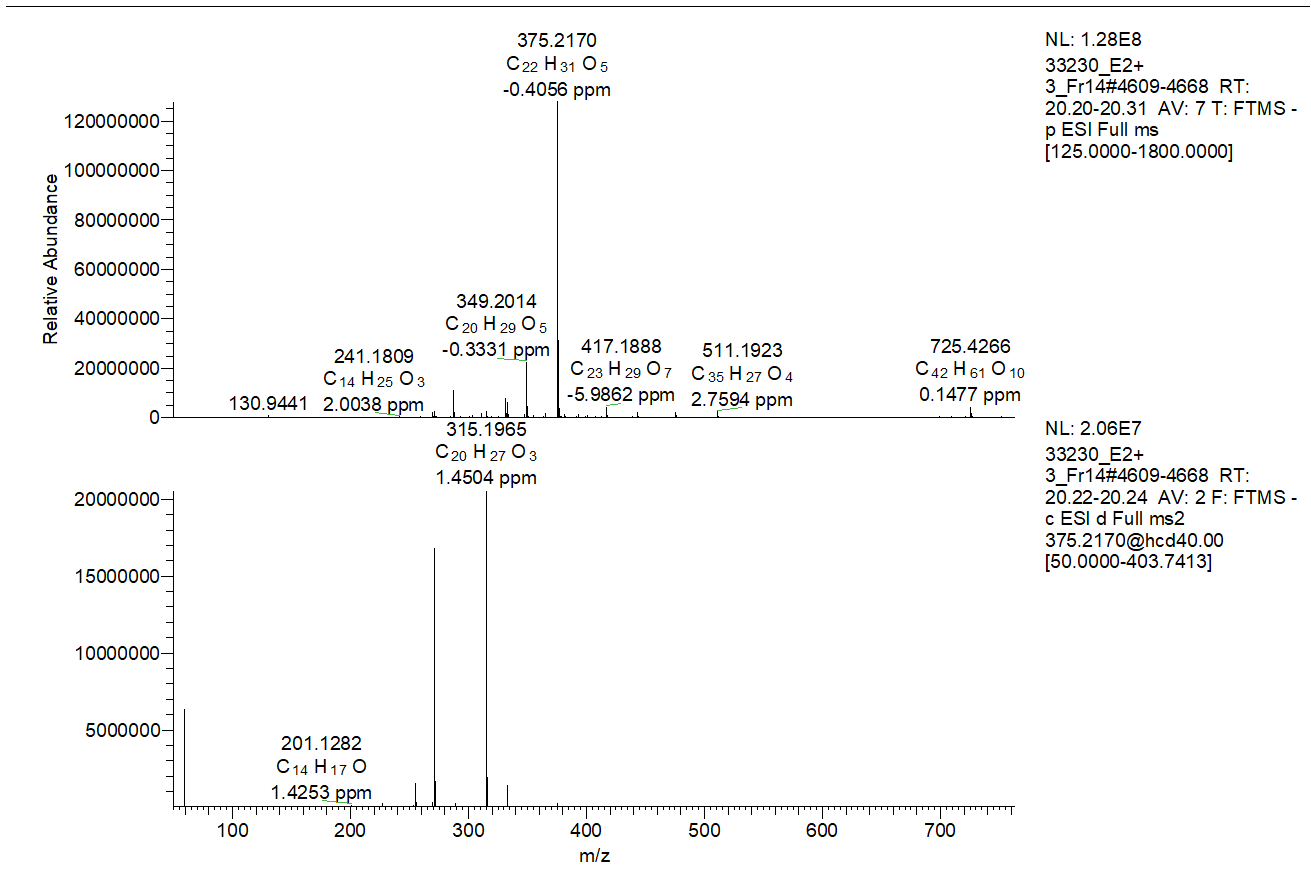


Figure S31. Full (-)-HRESI mass and MS2 spectra of **9**


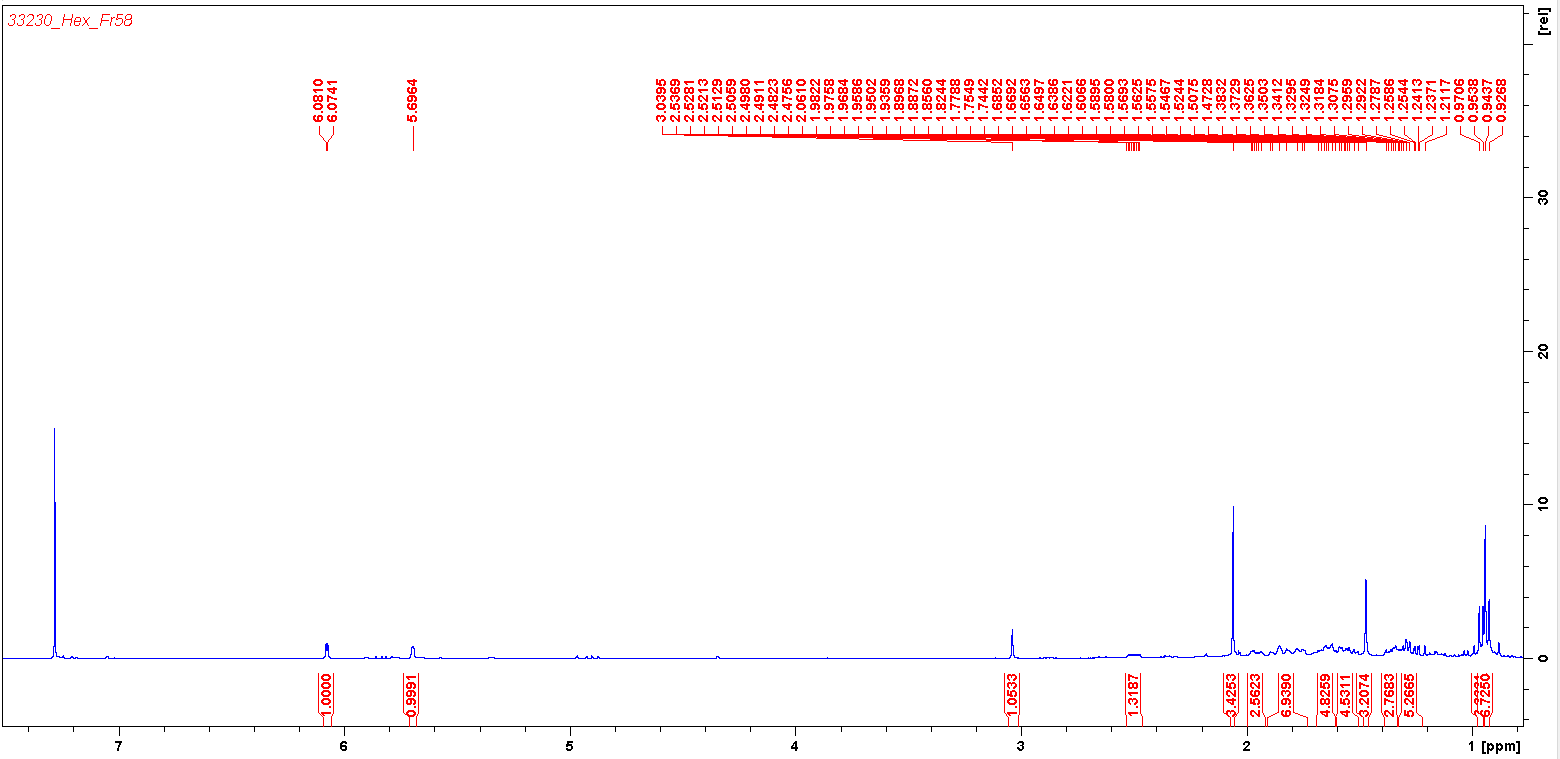


Figure S32. ^1^H NMR spectrum (400 MHz, CDCl_3_) of **9**


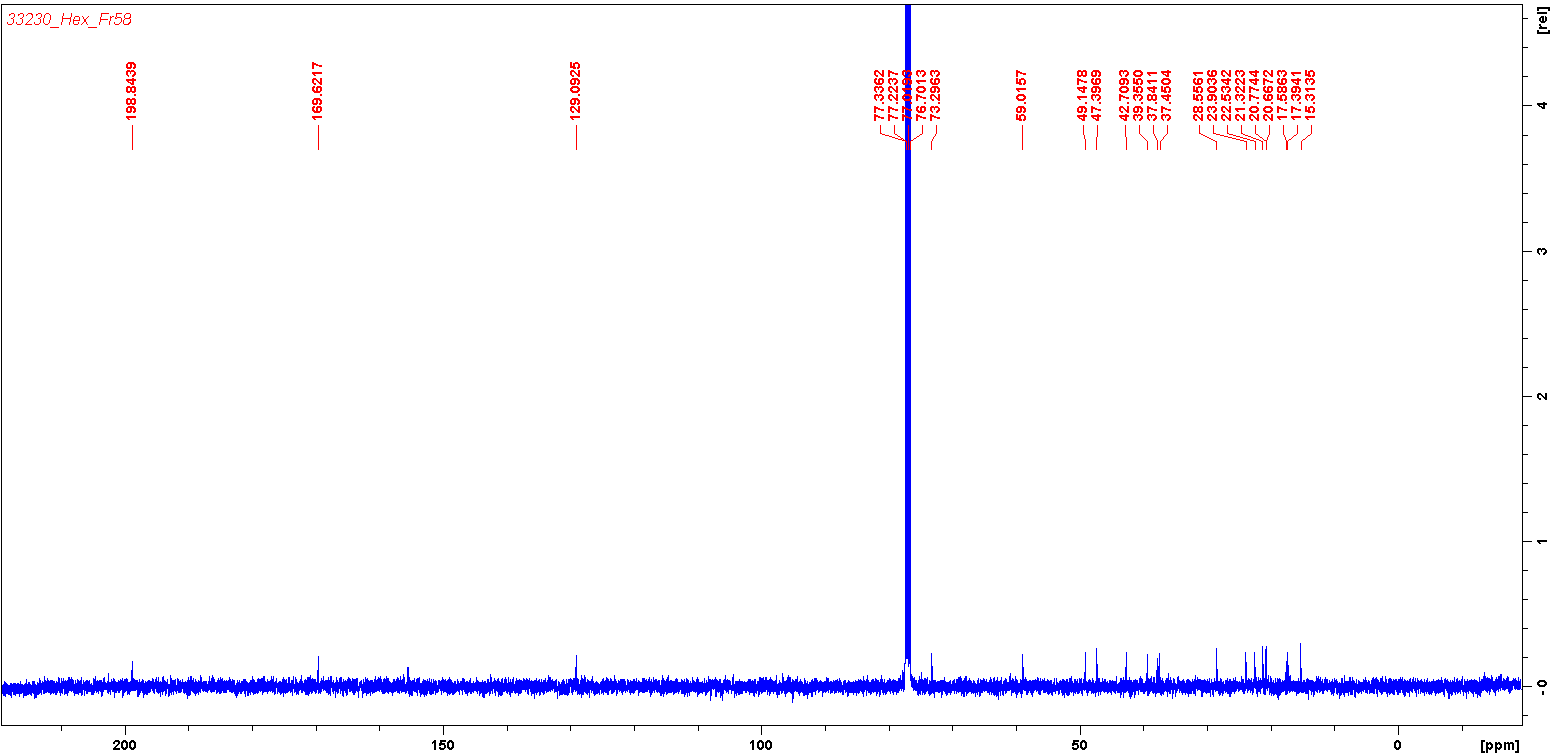


Figure S33. ^13^C NMR spectrum (100 MHz, CDCl_3_) of **9**


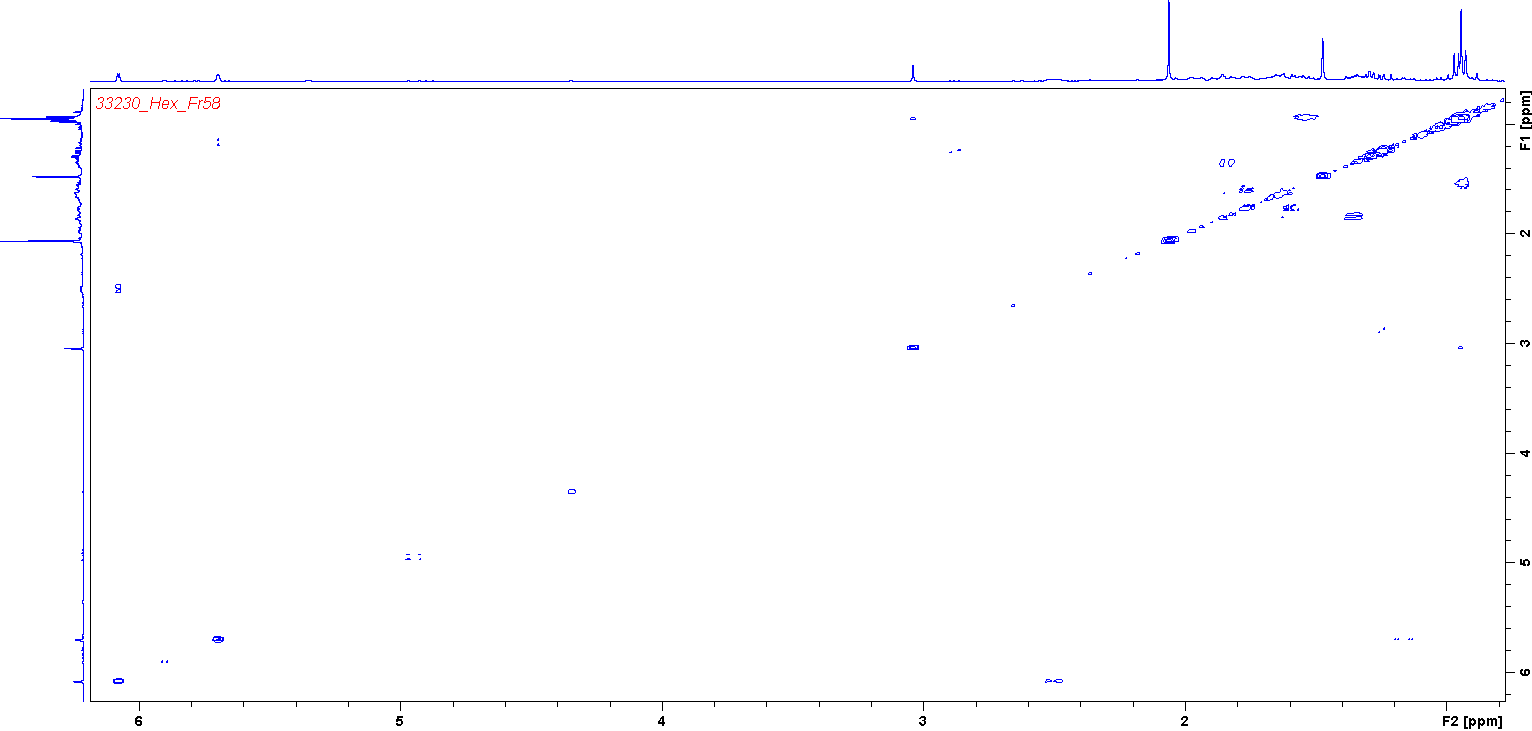


Figure S34. ^1^H,^1^H COSY spectrum of **9**


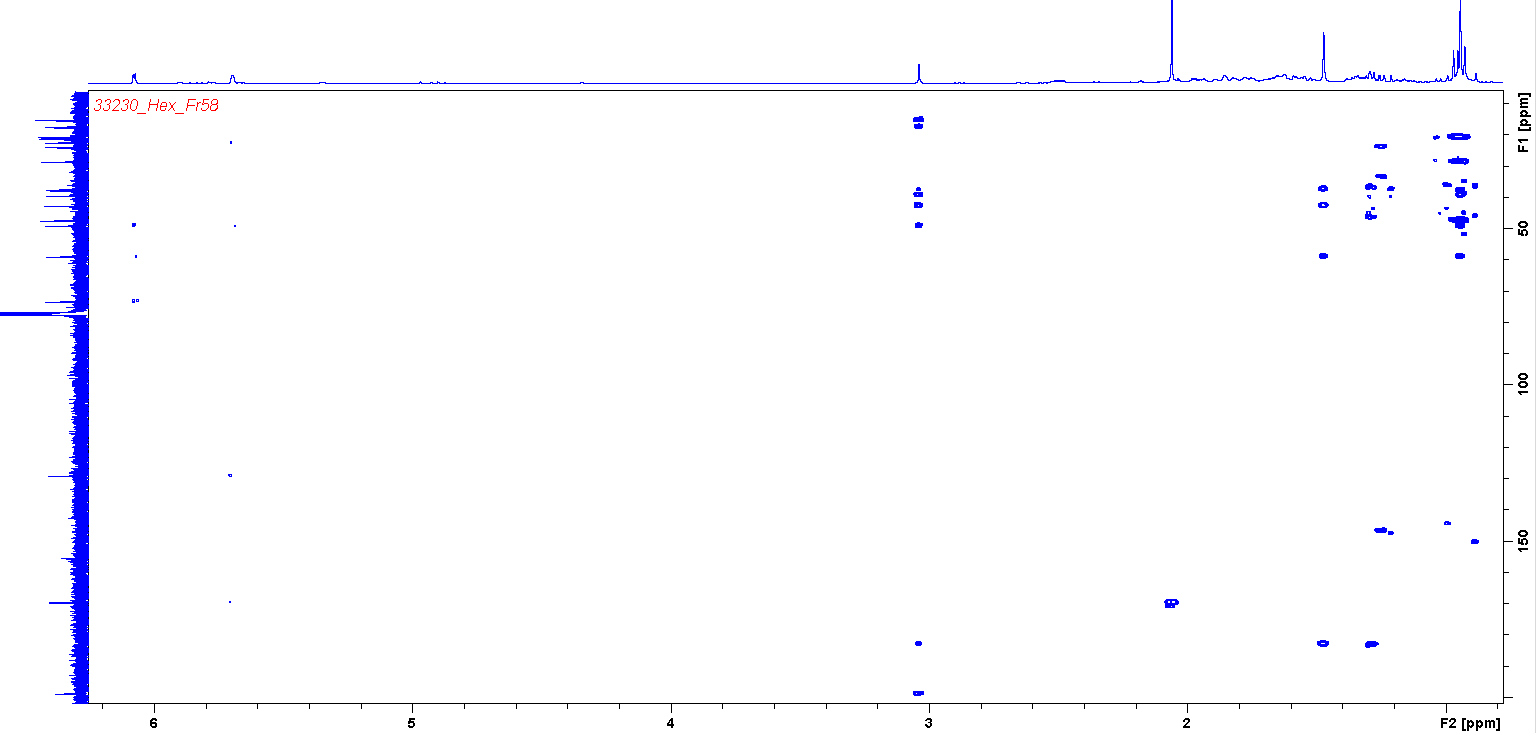


Figure S35. HMBC spectrum of **9**


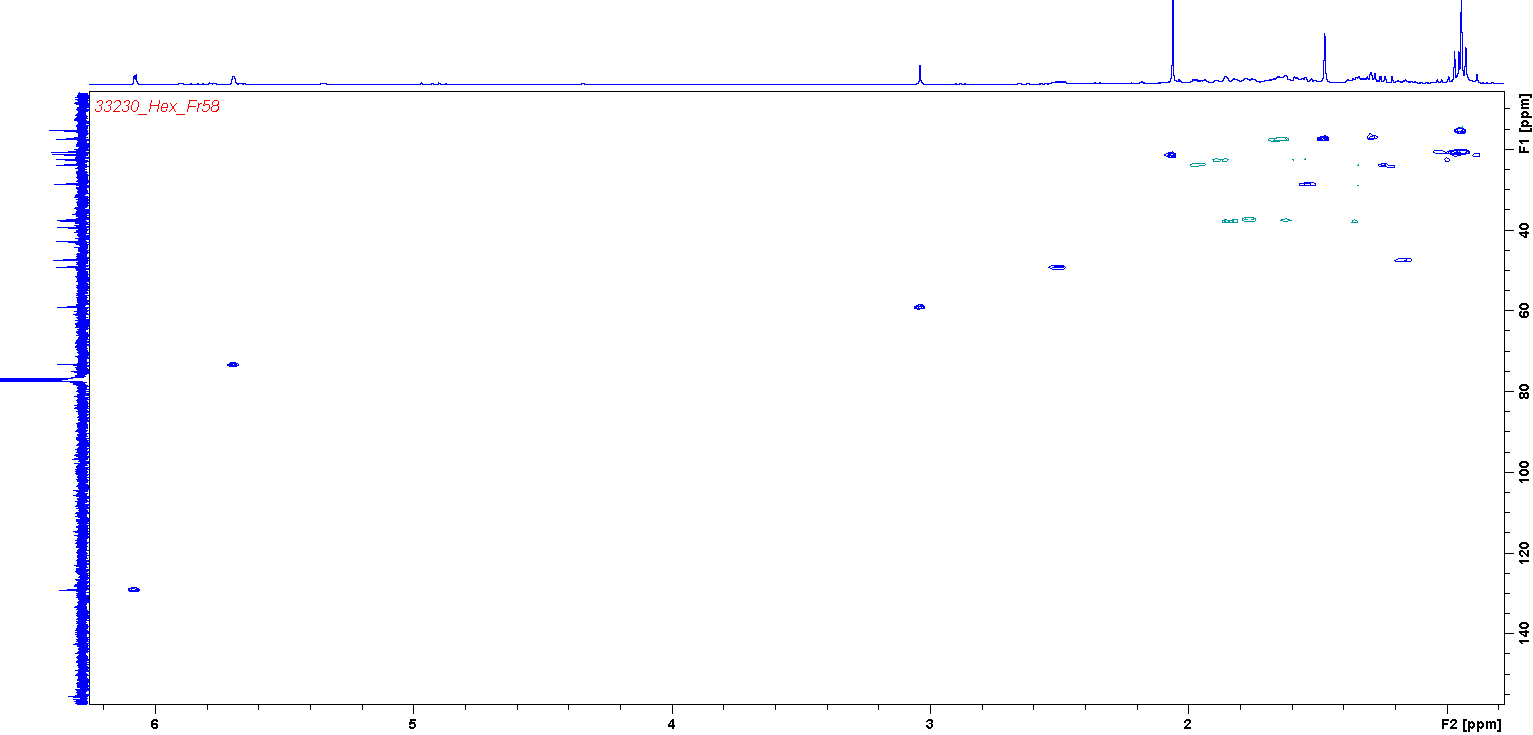


Figure S36. HSQC spectrum of **9**


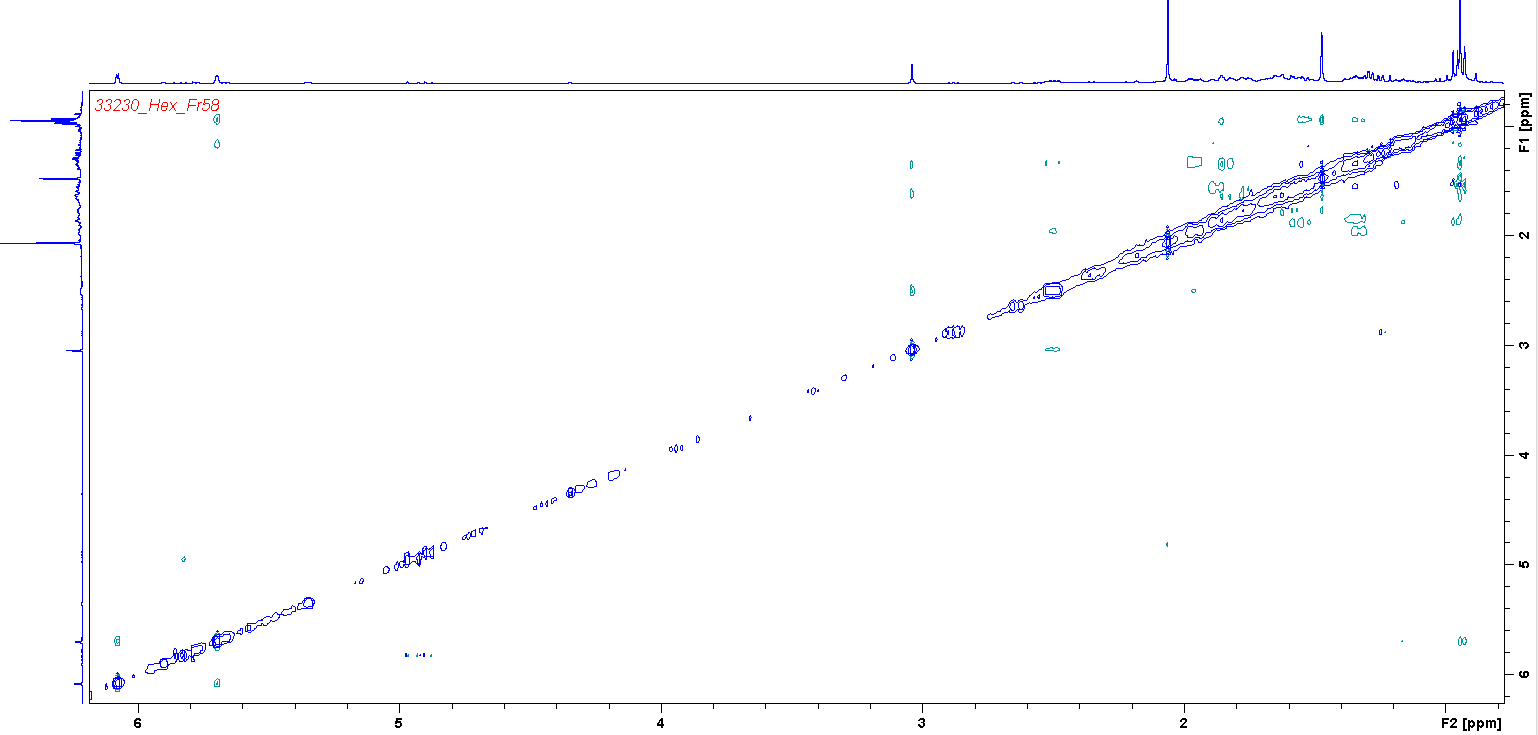


Figure S37. NOESY spectrum of **9**


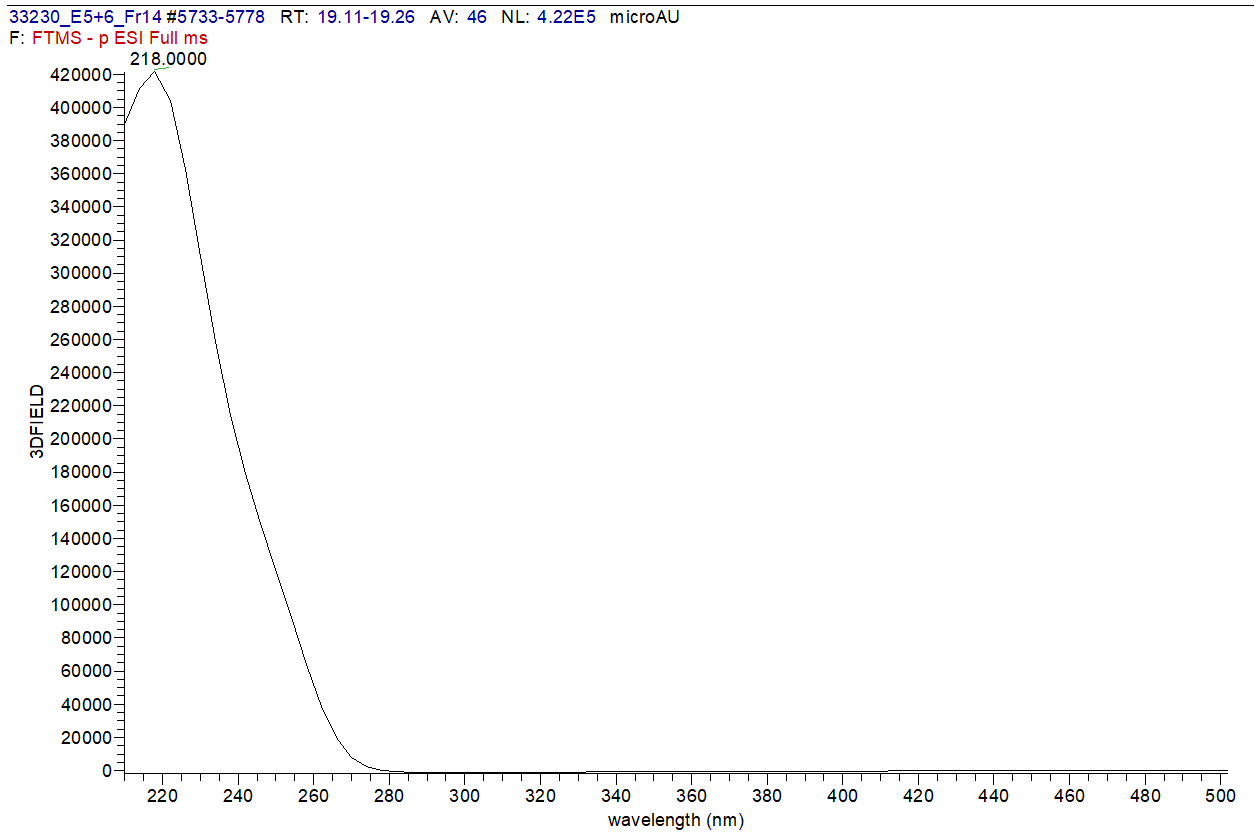


Figure S38. UV spectrum of **10**


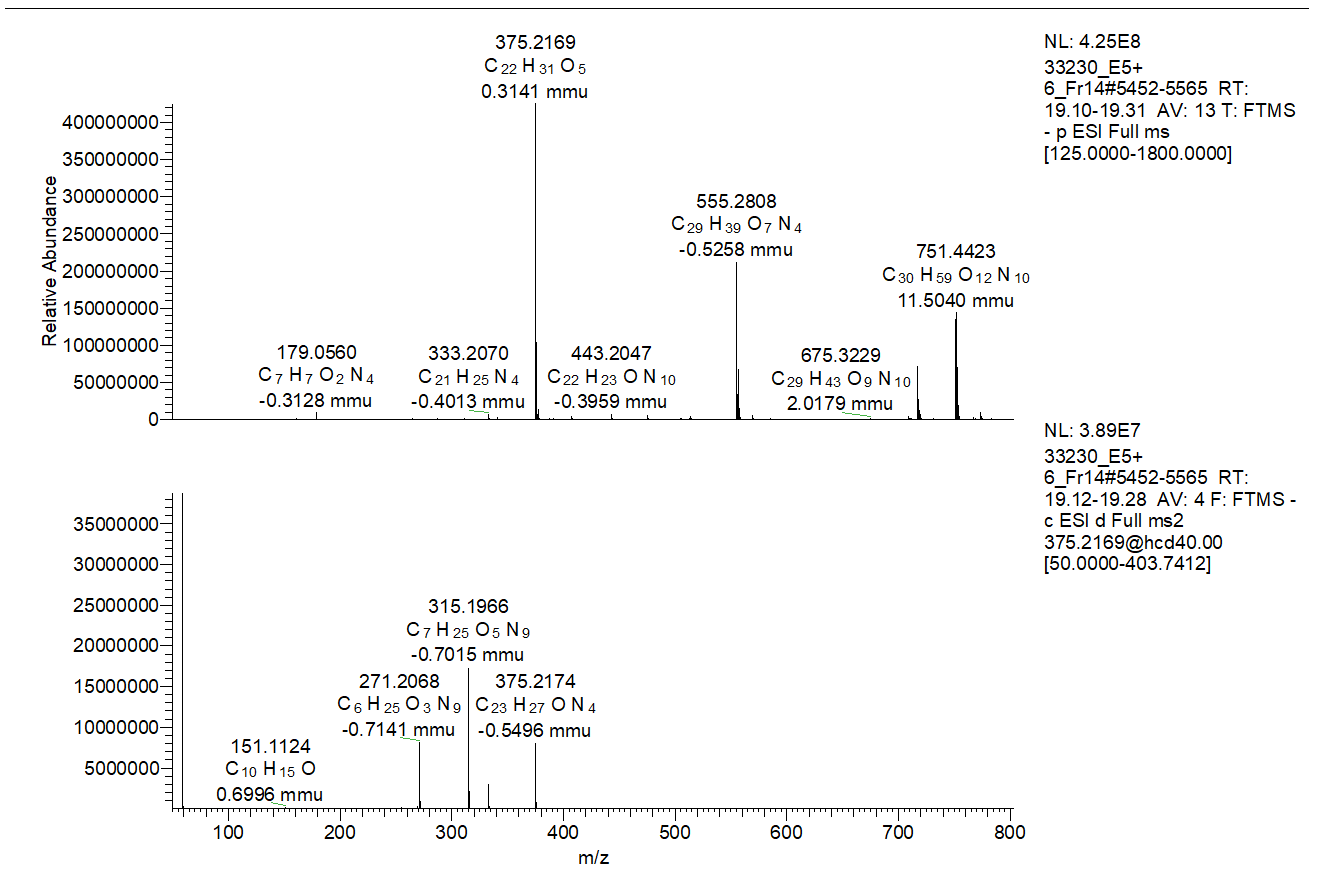


Figure S39. Full (-)-HRESI mass and MS2 spectra of **10**


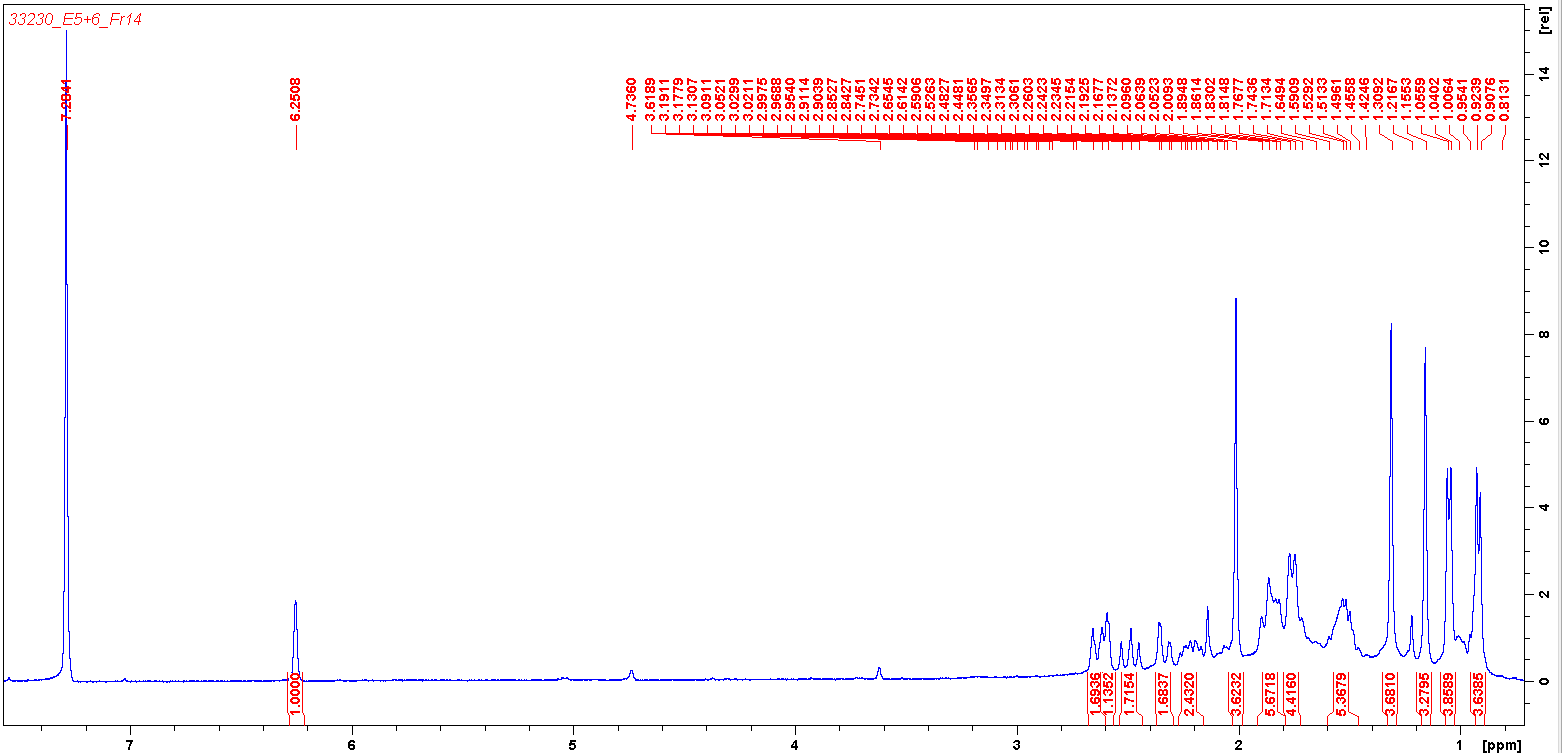


Figure S40. ^1^H NMR spectrum (400 MHz, CDCl_3_) of **10**


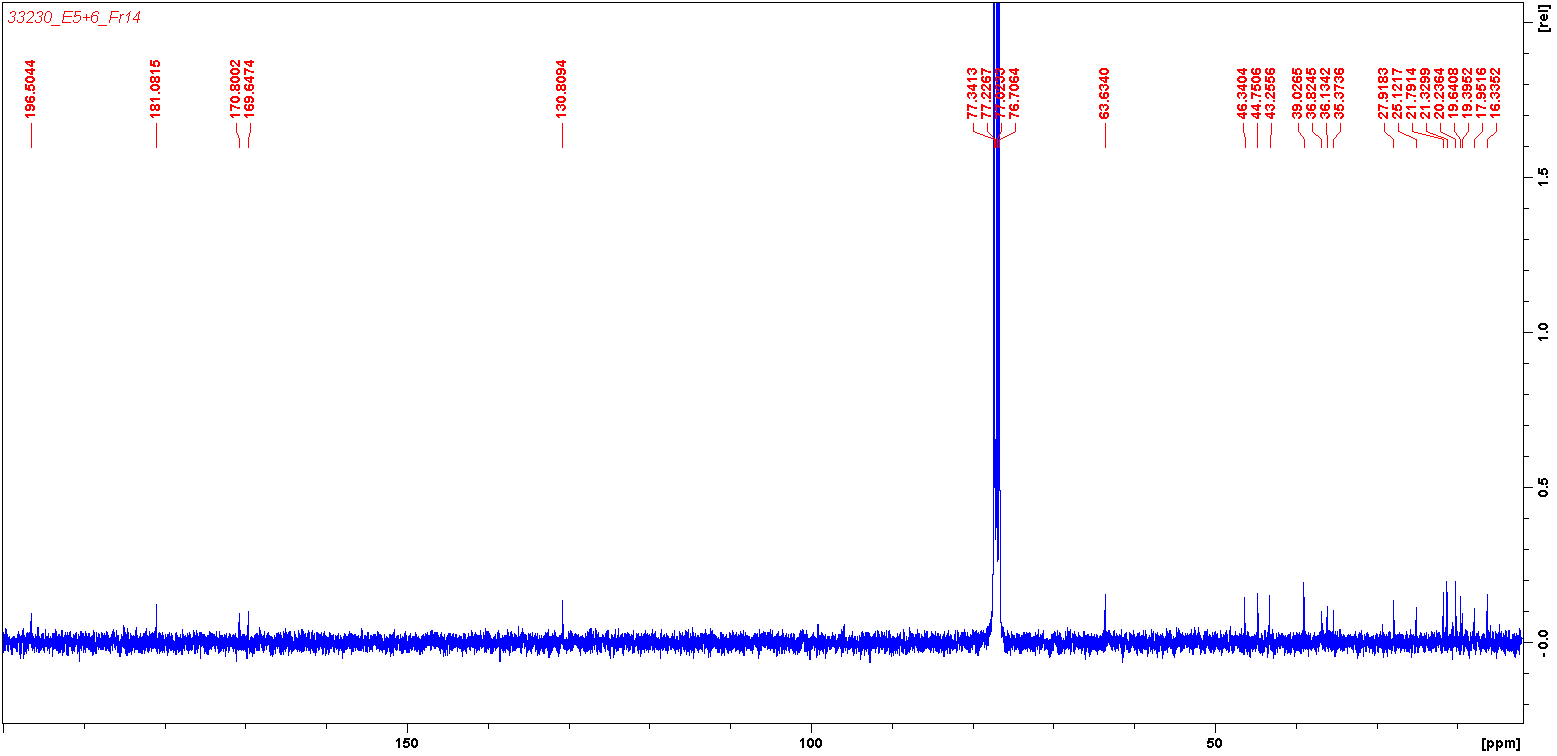


Figure S41. ^13^C NMR spectrum (100 MHz, CDCl_3_) of **10**


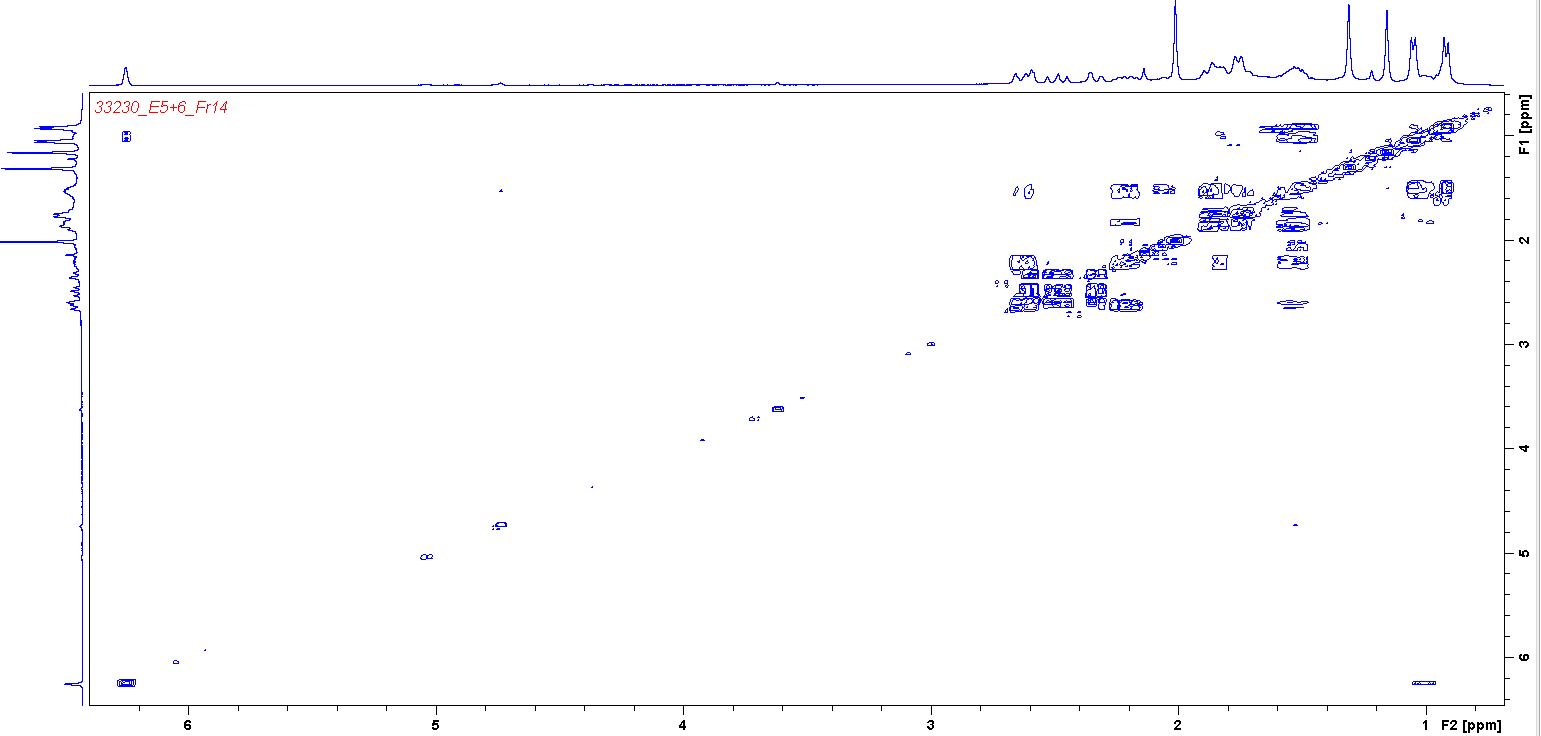


Figure S42. ^1^H,^1^H COSY spectrum of **10**


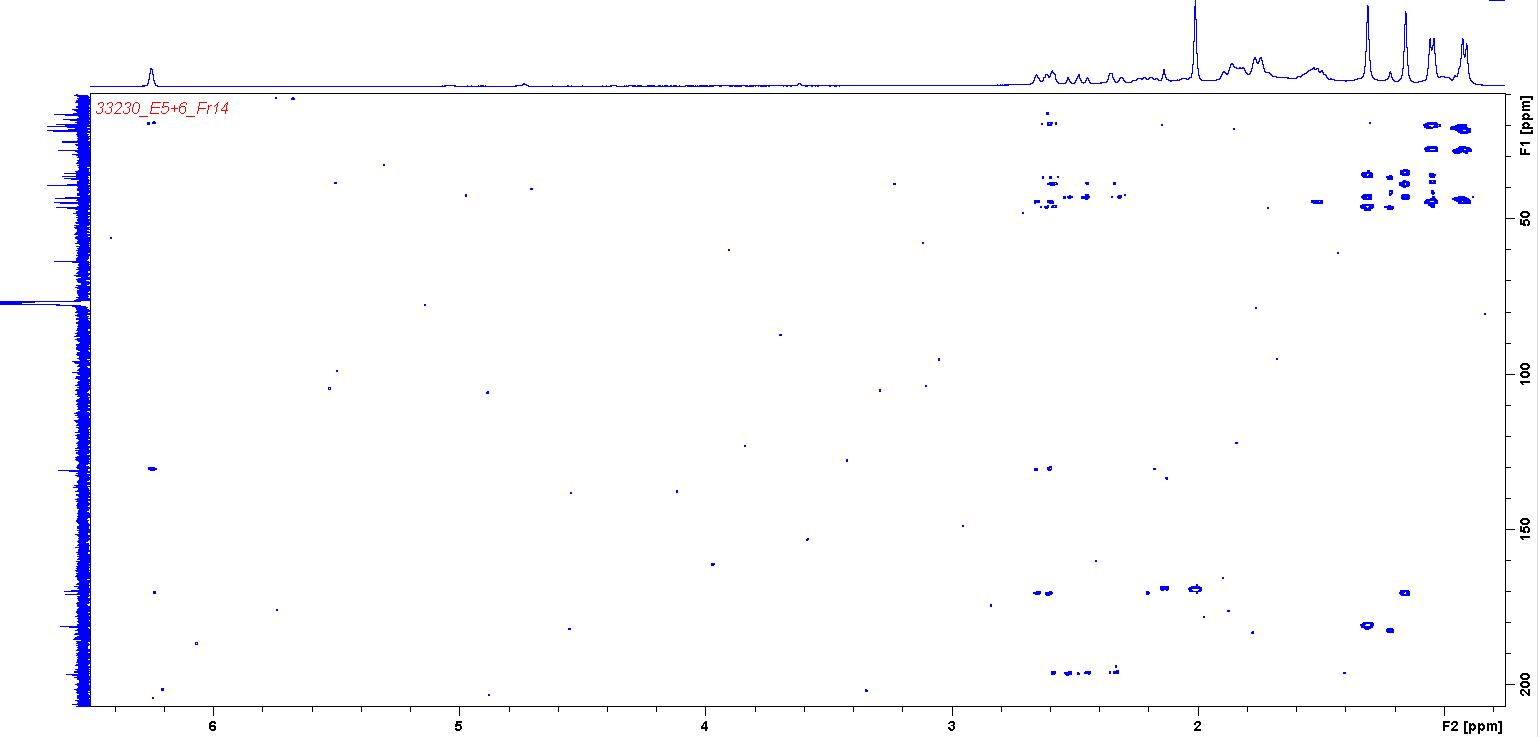


Figure S43. HMBC spectrum of **10**


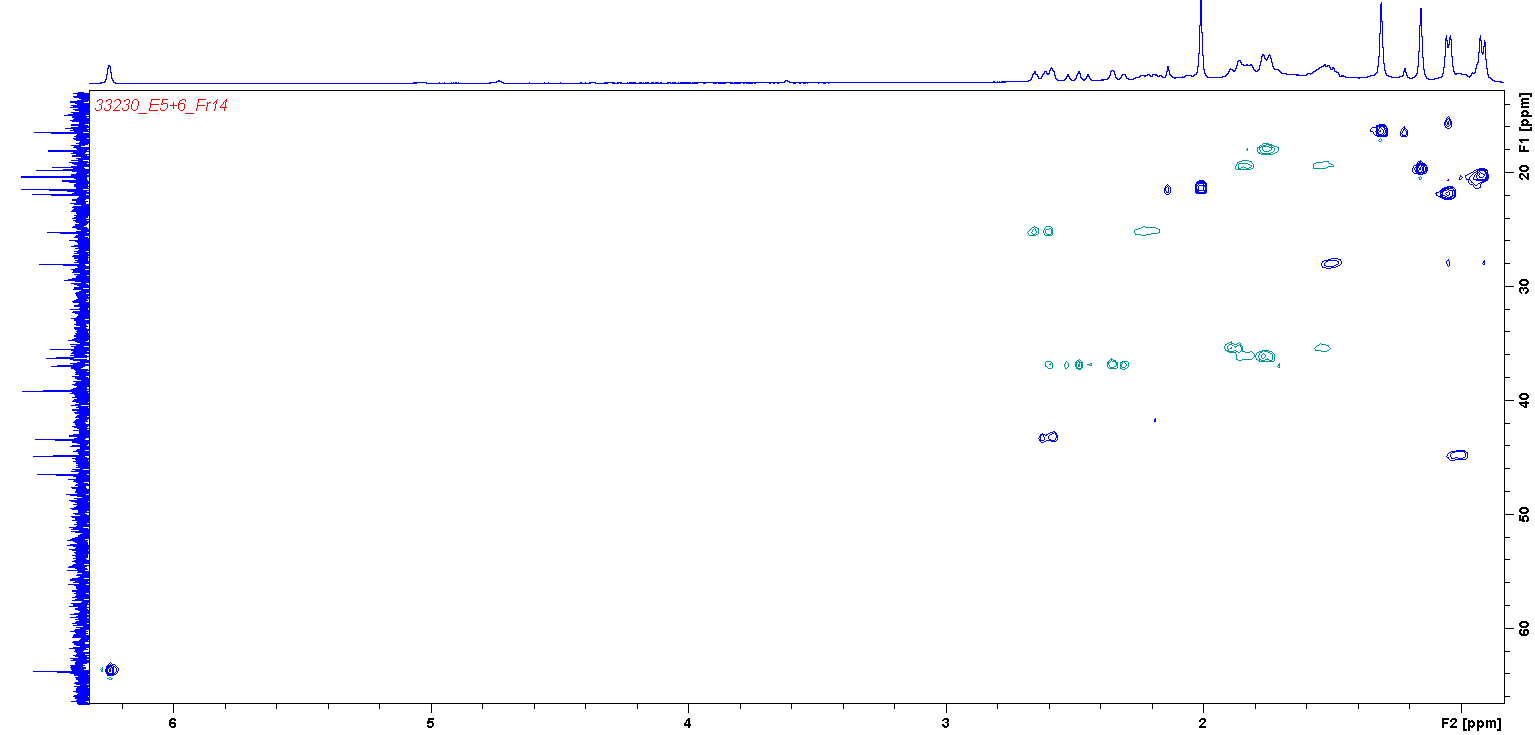


Figure S44. HSQC spectrum of **10**


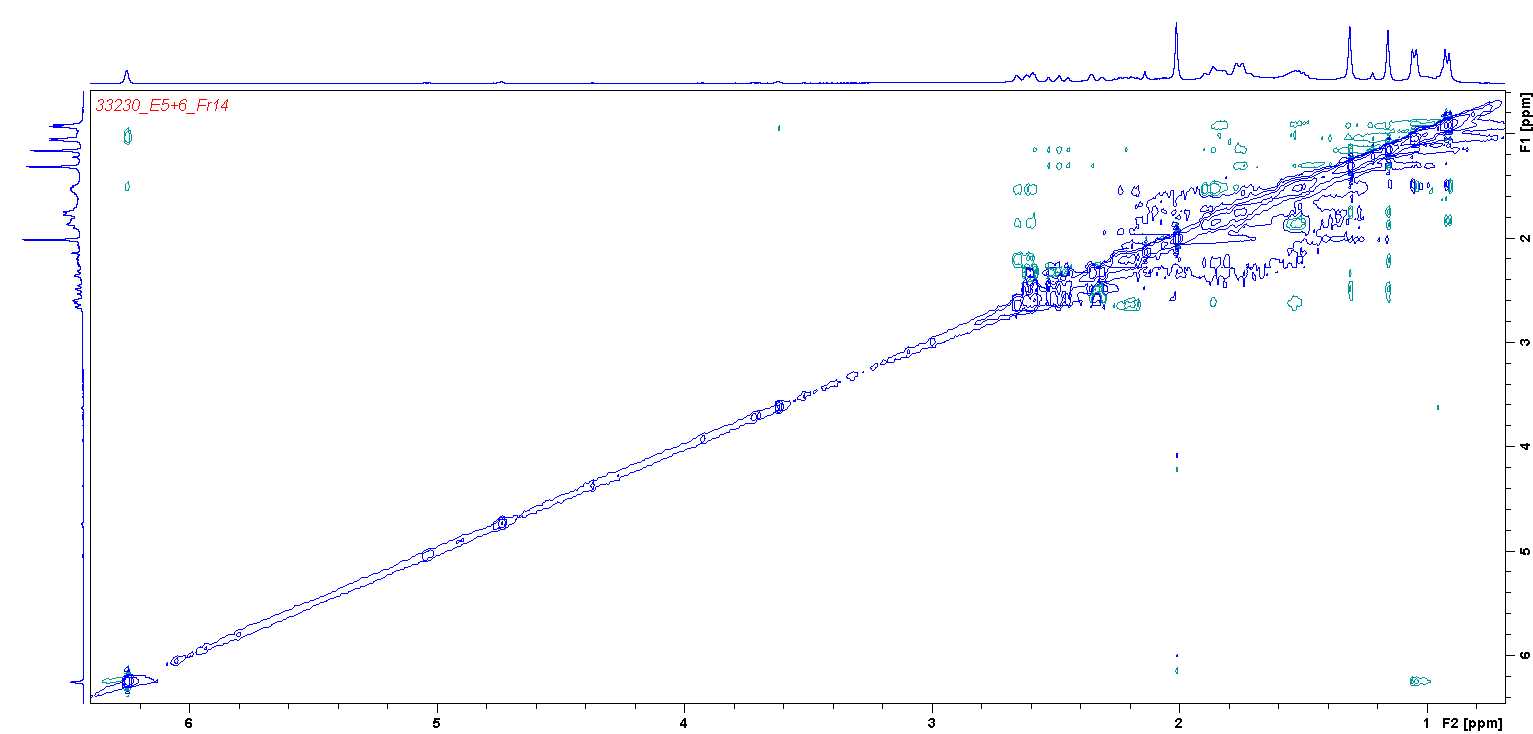


Figure S45. NOESY spectrum of **10**


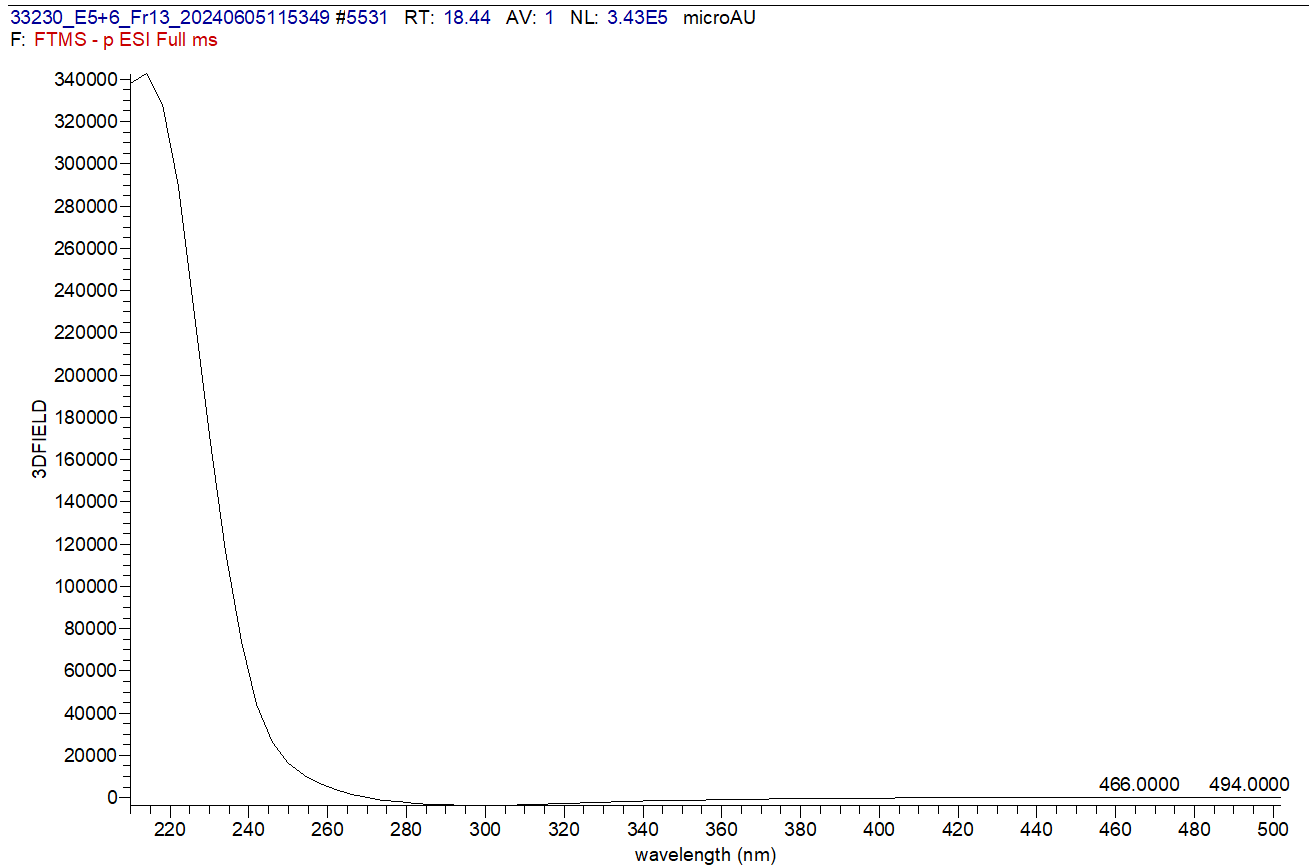


Figure S46. UV spectrum of the mixture of **8**+**11**


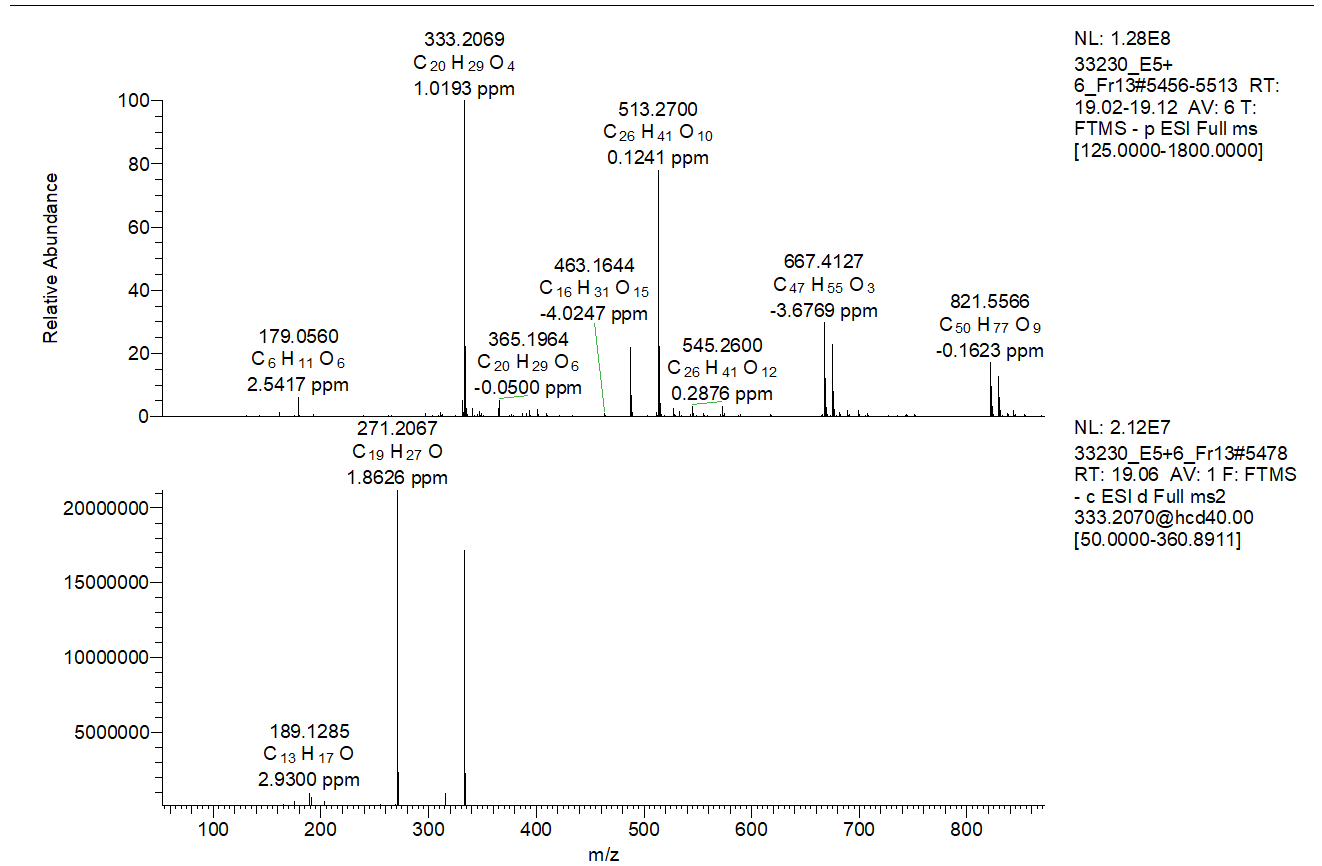


Figure S47. Full (-)-HRESI mass and MS2 spectra of **11**


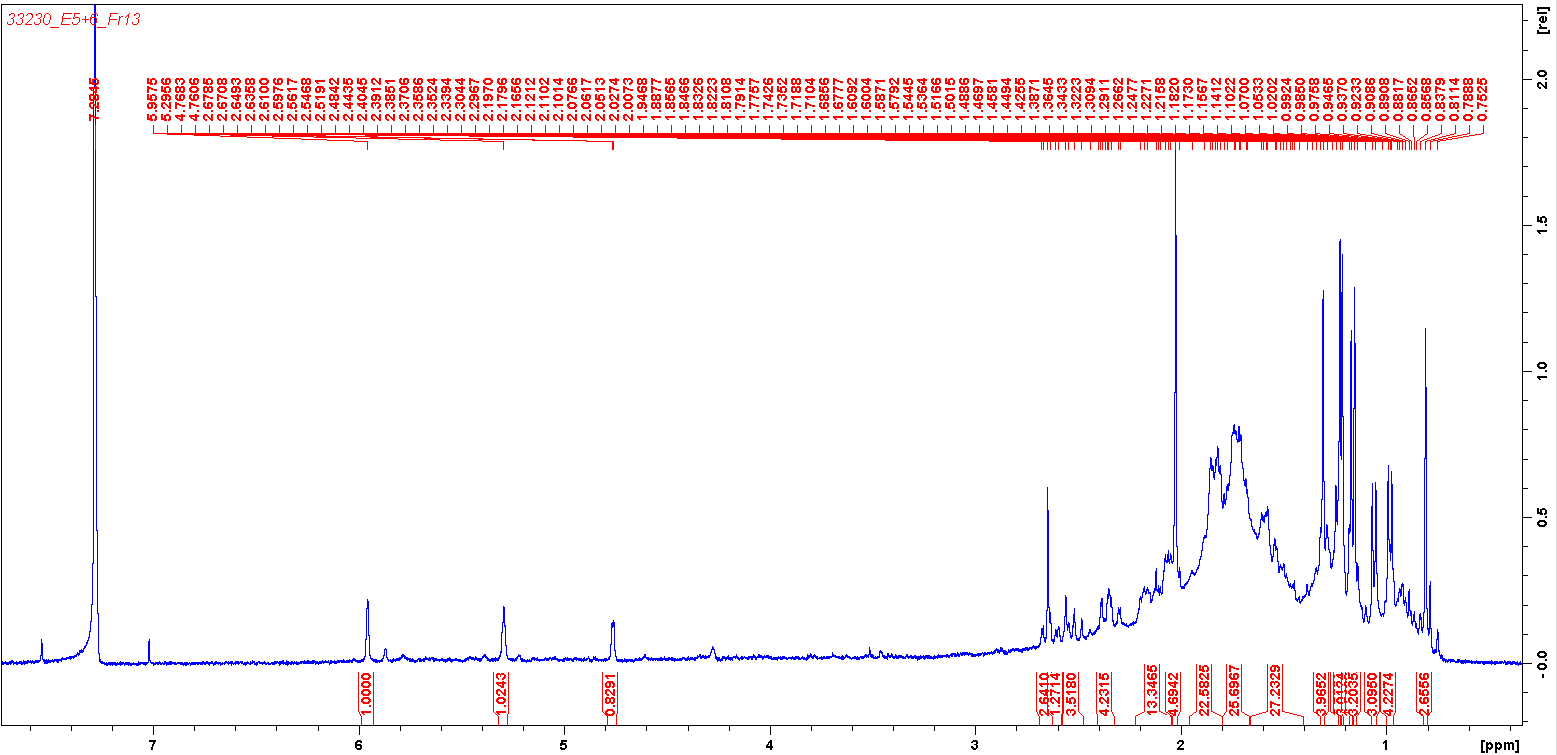


Figure S48. ^1^H NMR spectrum (400 MHz, CDCl_3_) of the mixture of **8**+**11**


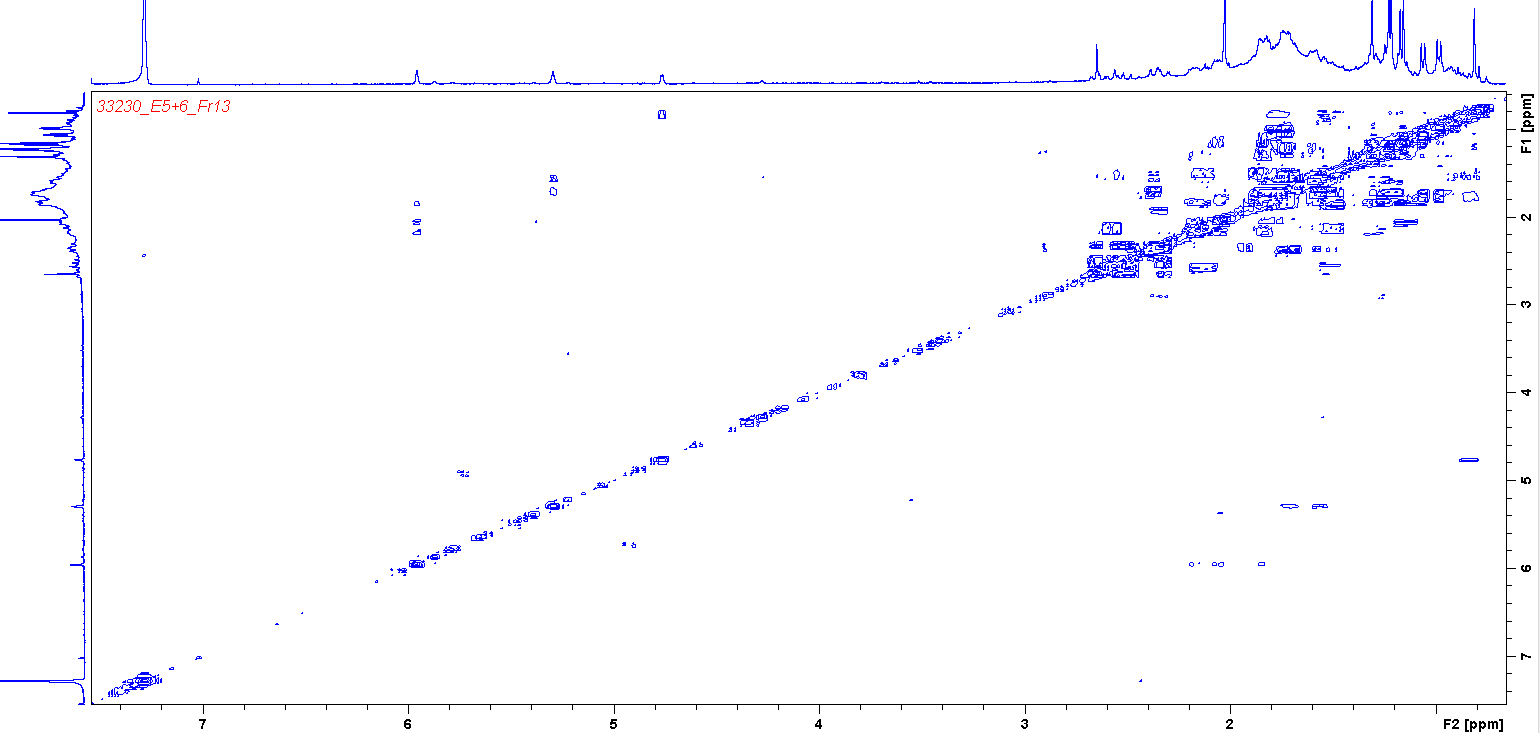


Figure S49. ^1^H,^1^H COSY spectrum of the mixture of **8**+**11**


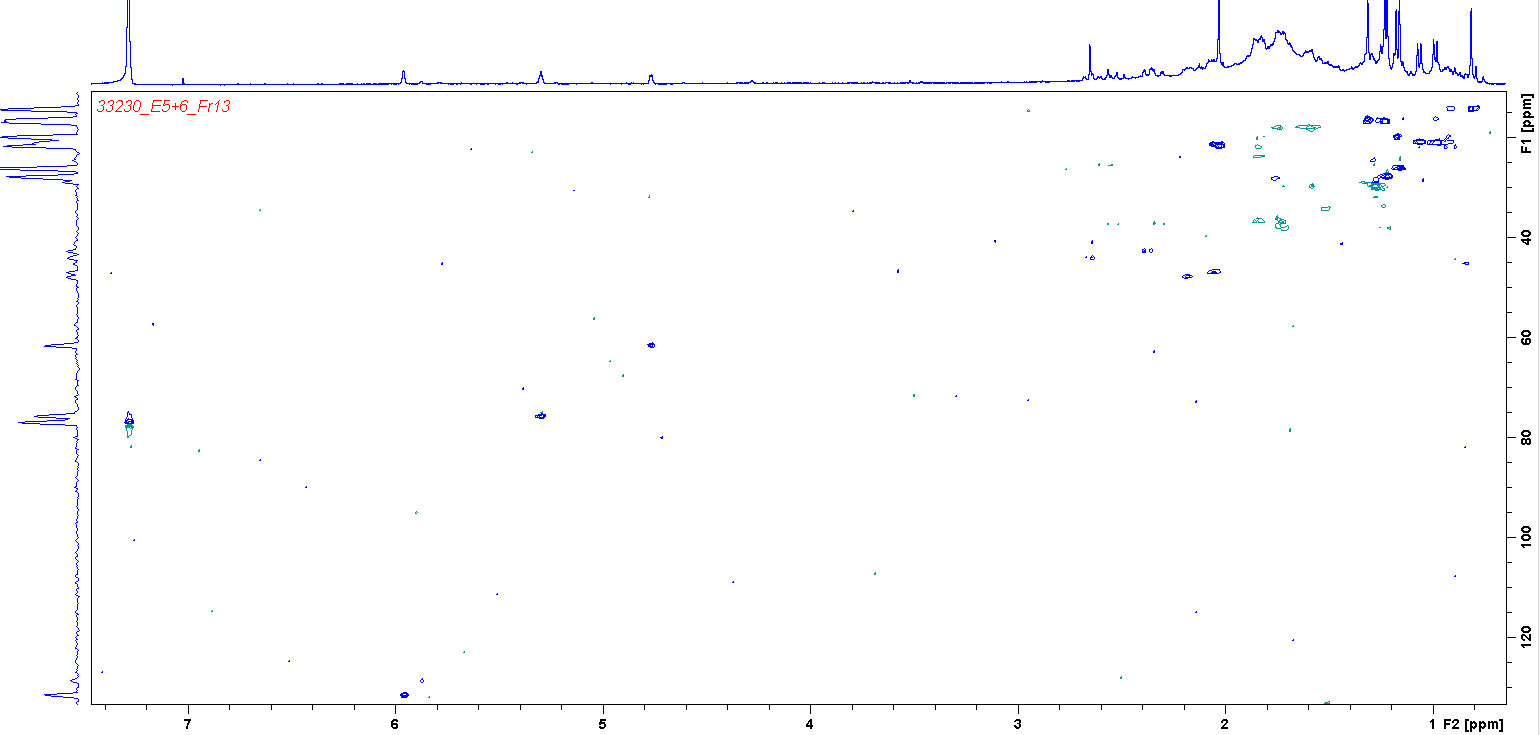


Figure S50. HSQC spectrum of the mixture of **8**+**11**


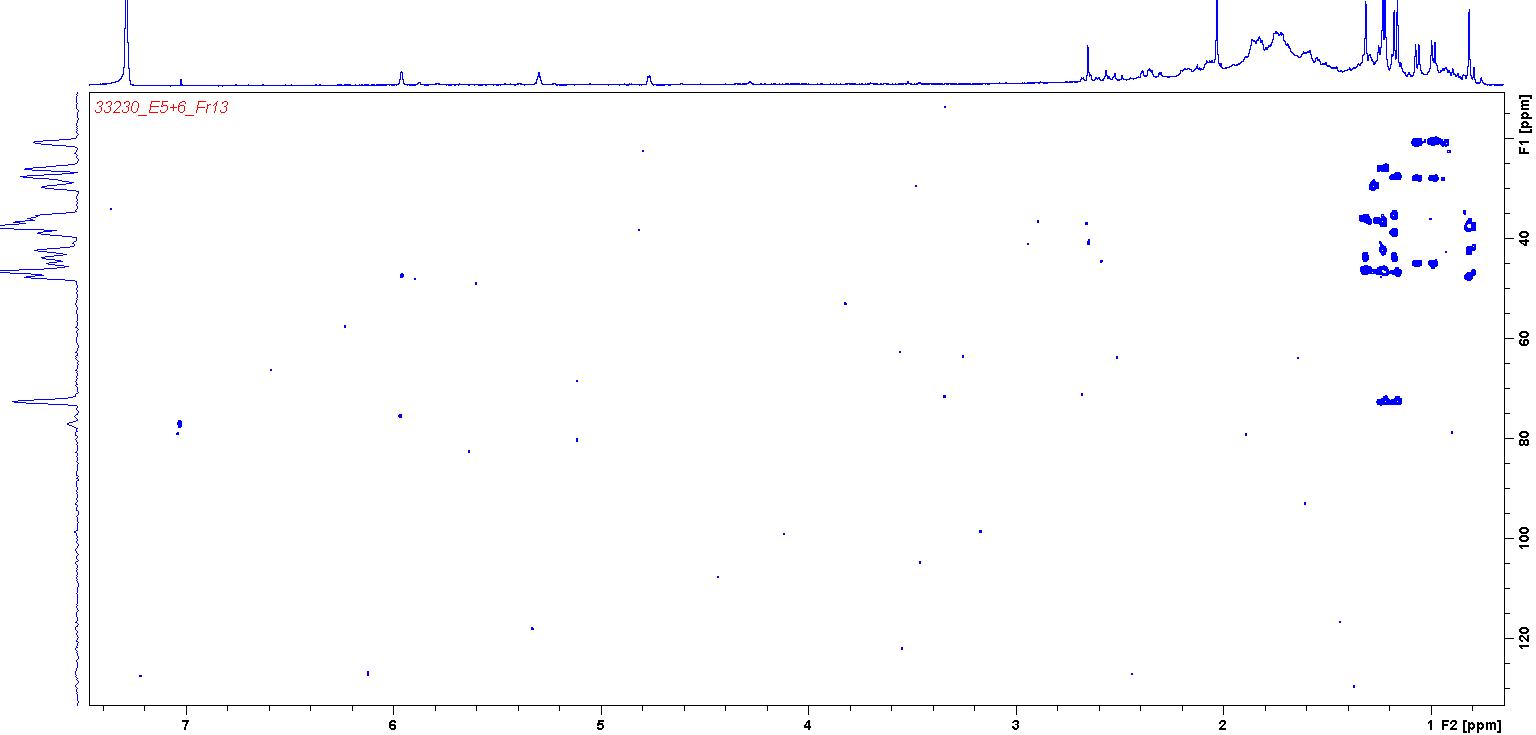


Figure S51. HMBC spectrum of the mixture of **8**+**11**


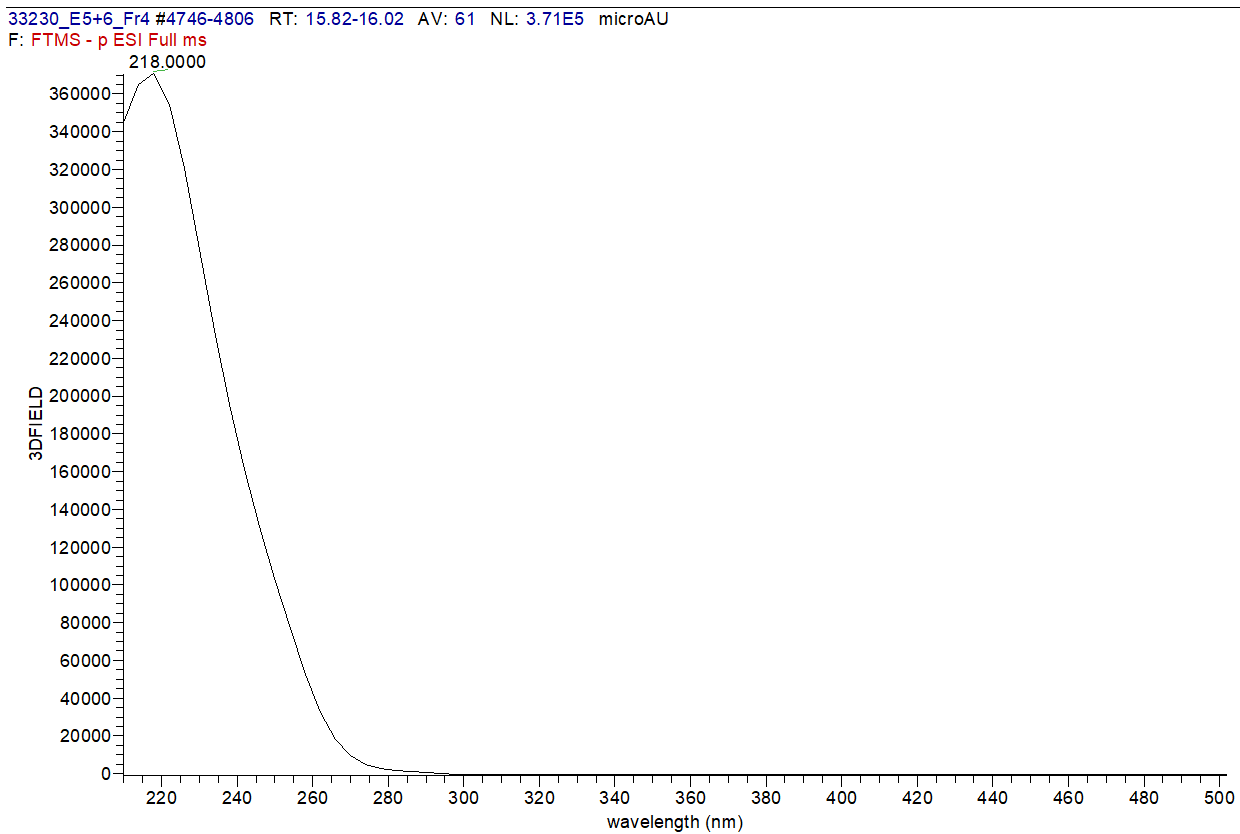


Figure S52. UV spectrum of **12**


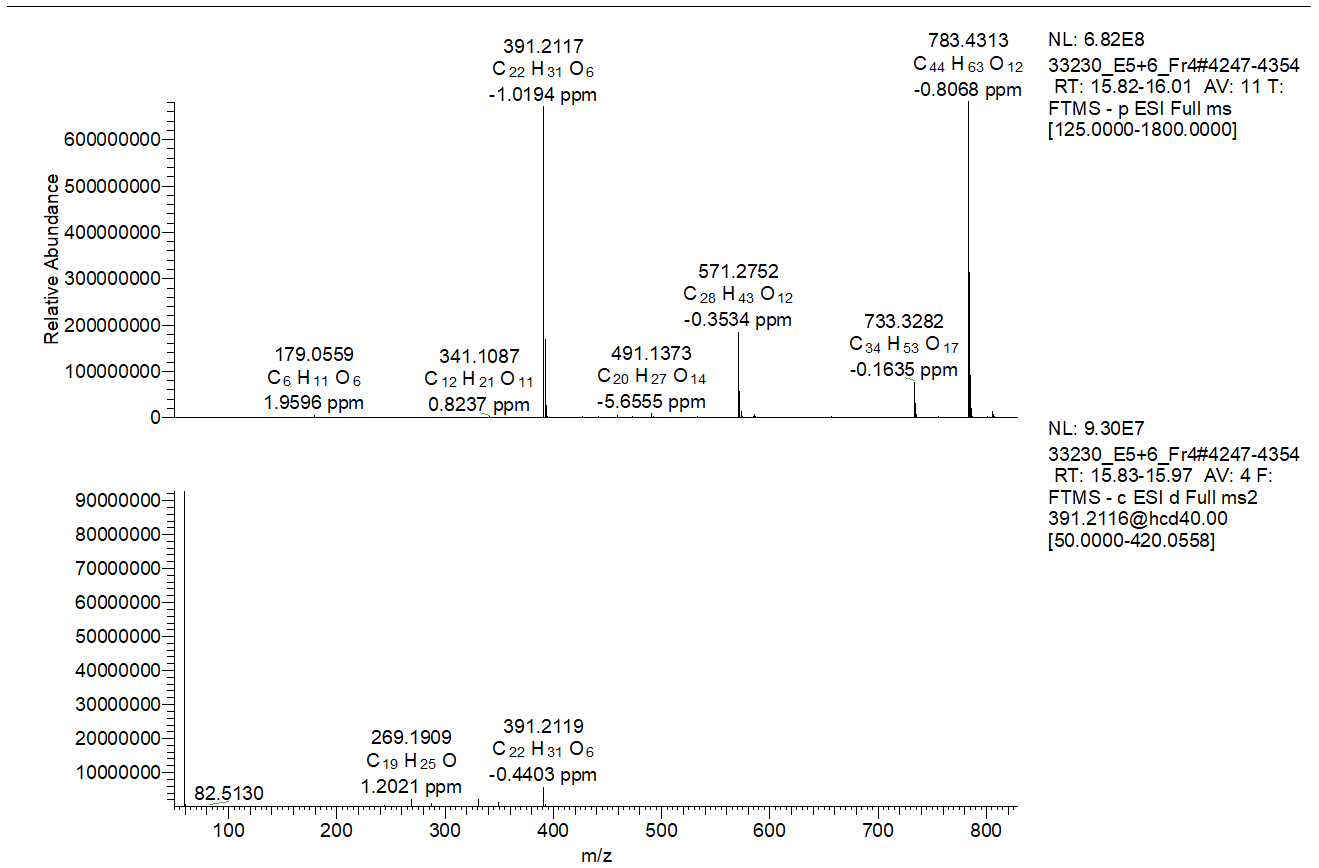


Figure S53. Full (-)-HRESI mass and MS2 spectra of **12**


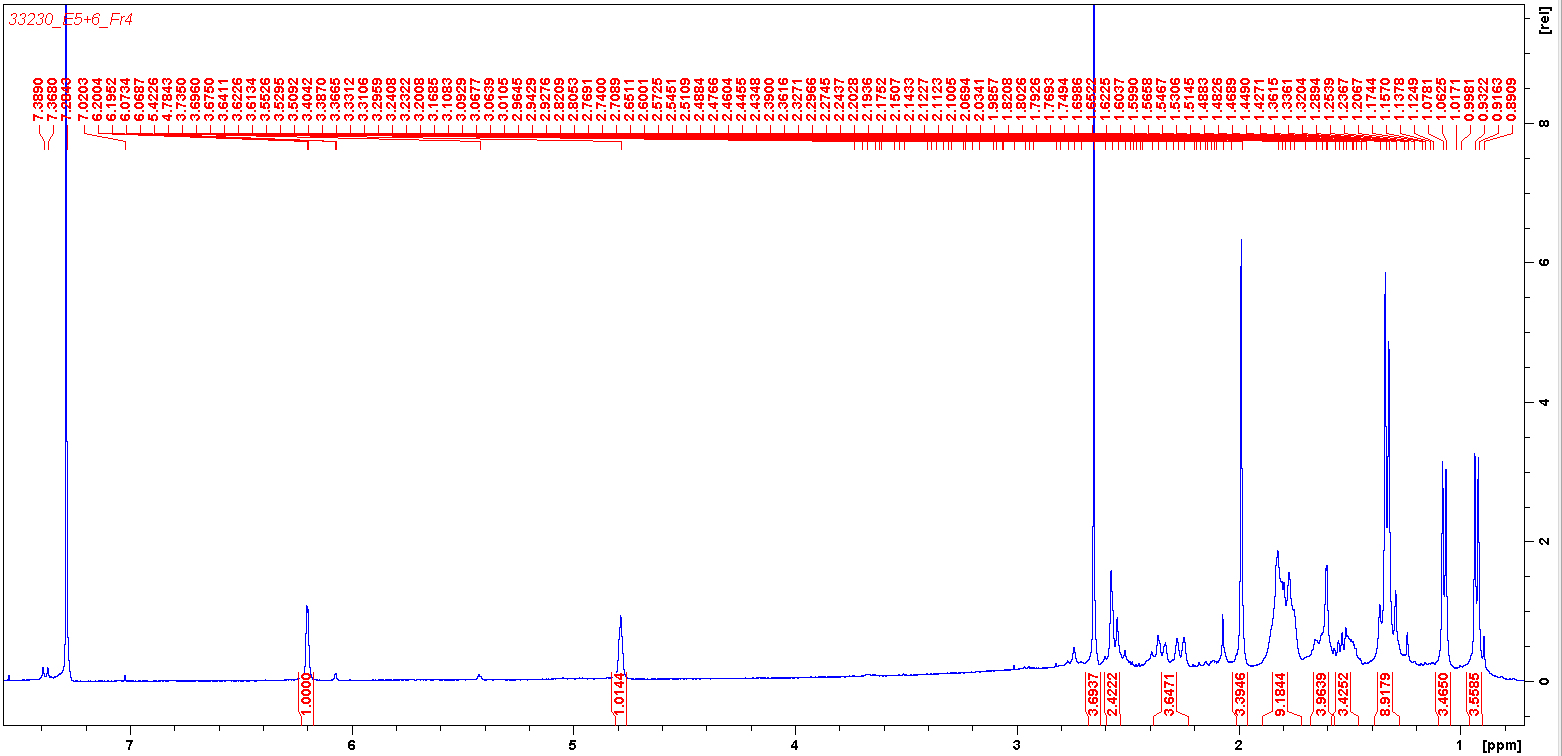


Figure S54. ^1^H NMR spectrum (400 MHz, CDCl_3_) of **12**


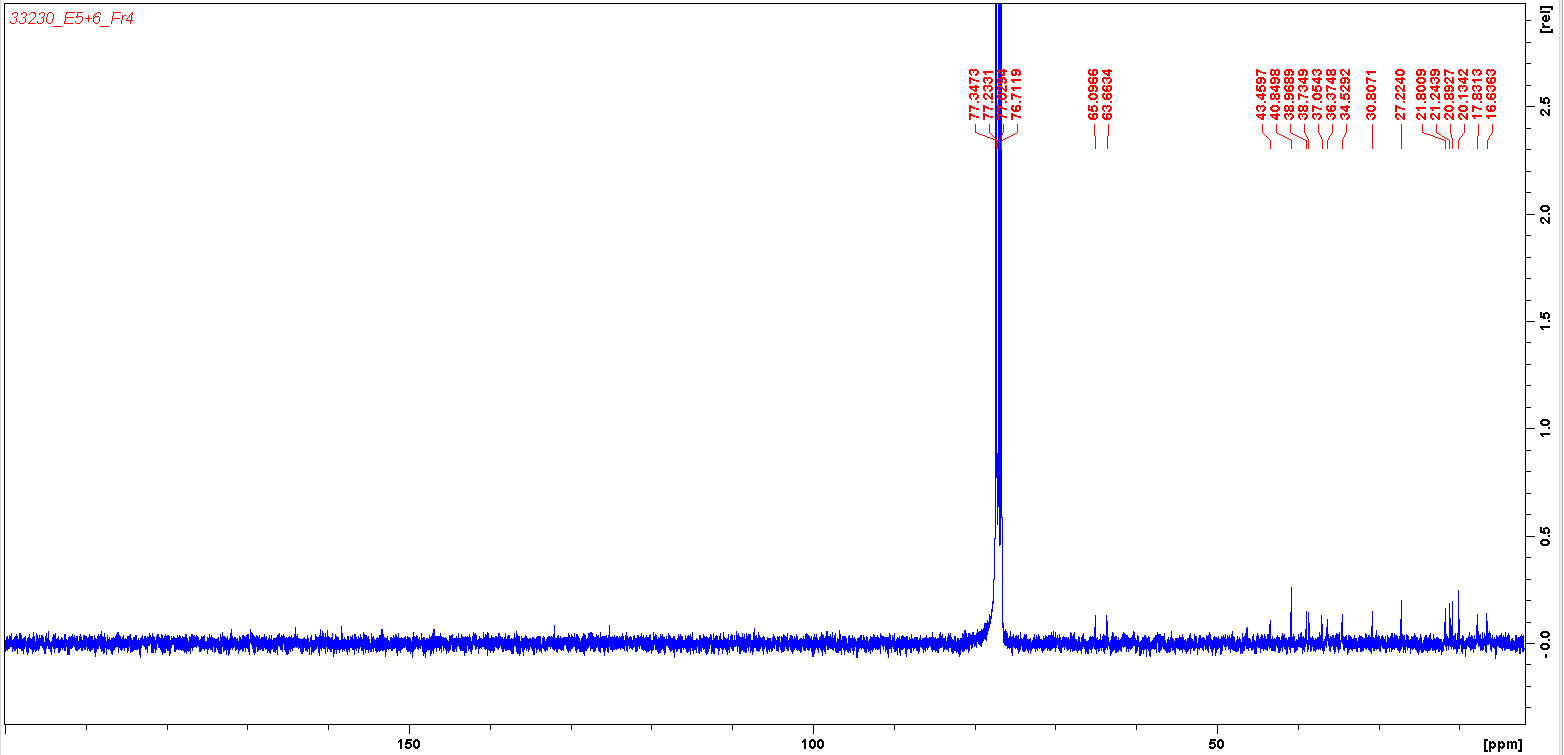


Figure S55. ^13^C NMR spectrum (100 MHz, CDCl_3_) of **12**


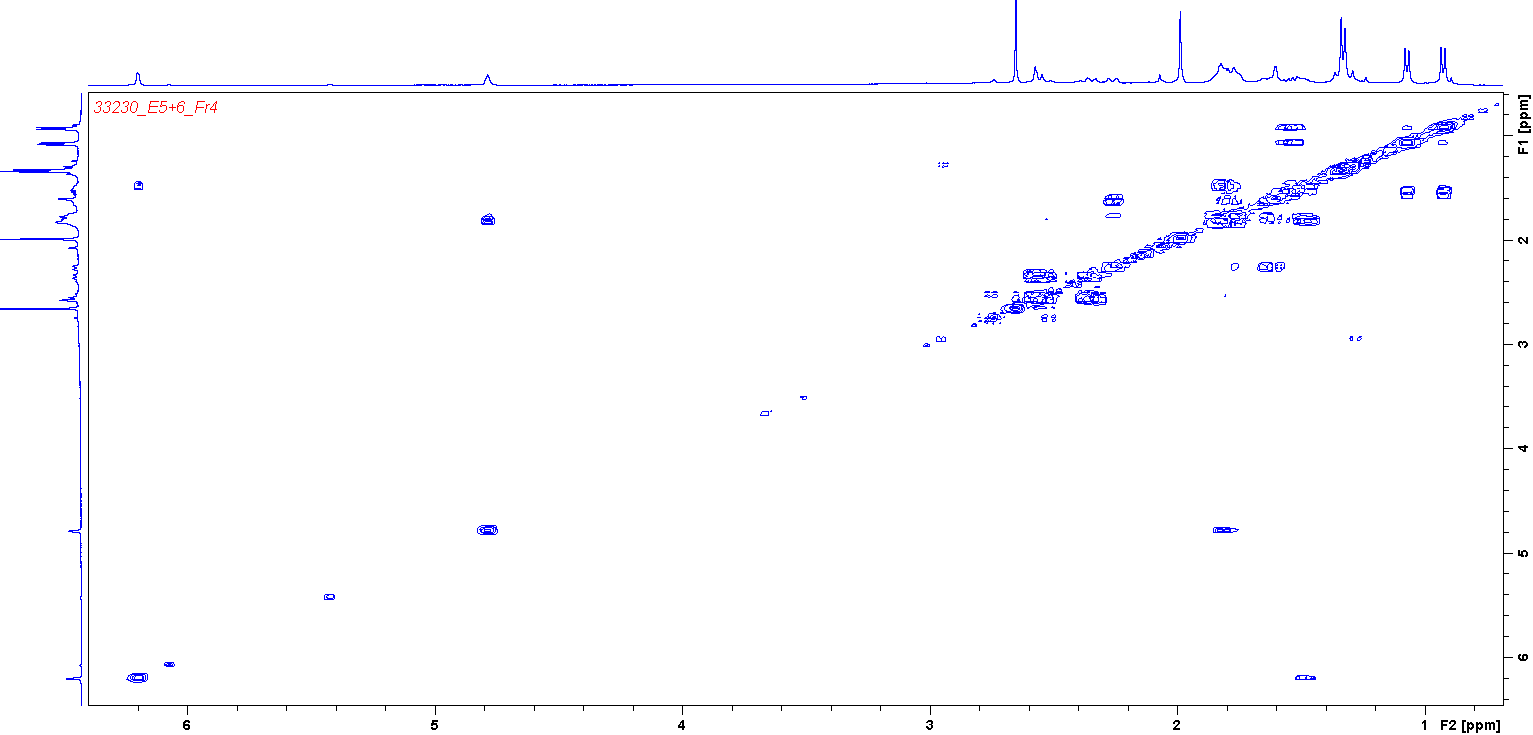


Figure S56. ^1^H,^1^H COSY spectrum of **12**


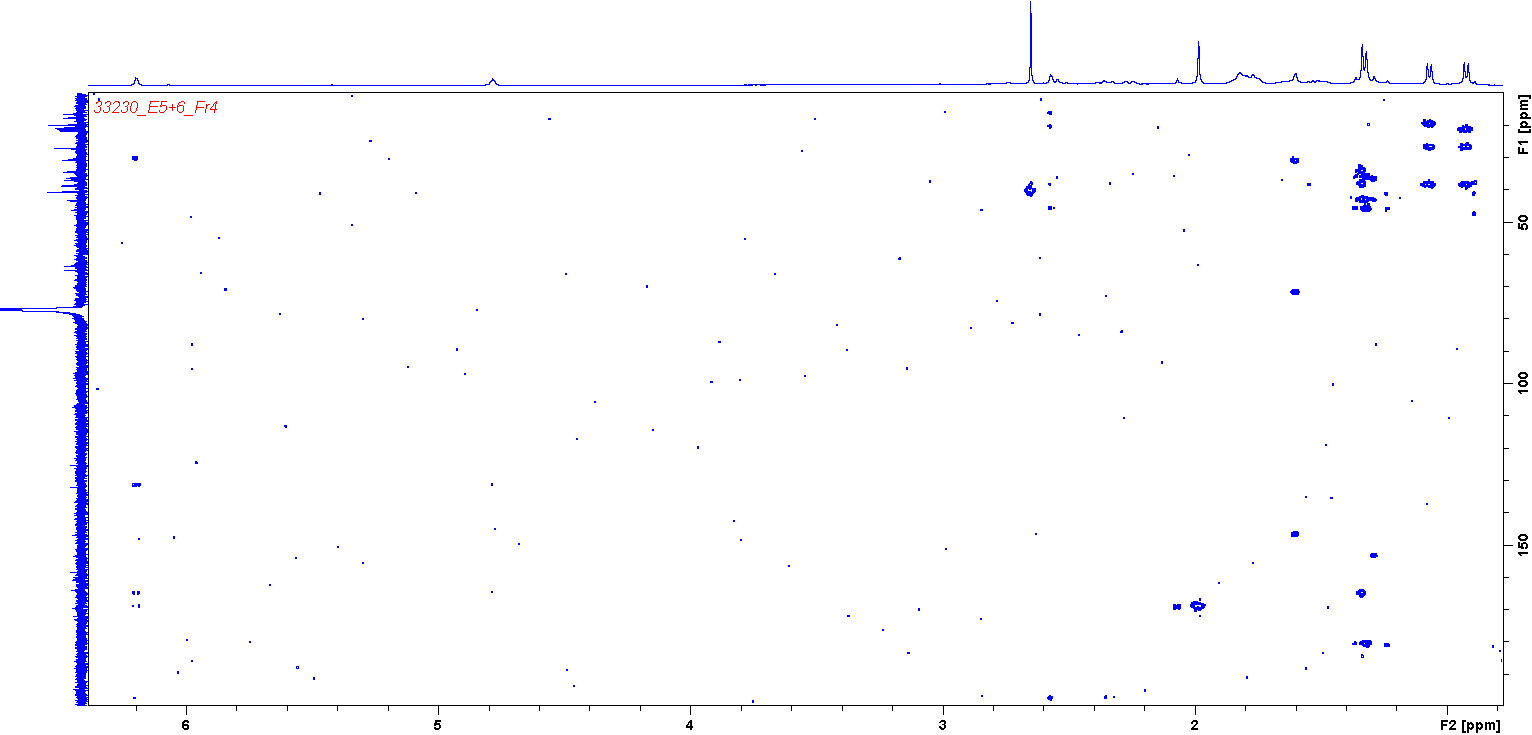


Figure S57. HMBC spectrum of **12**


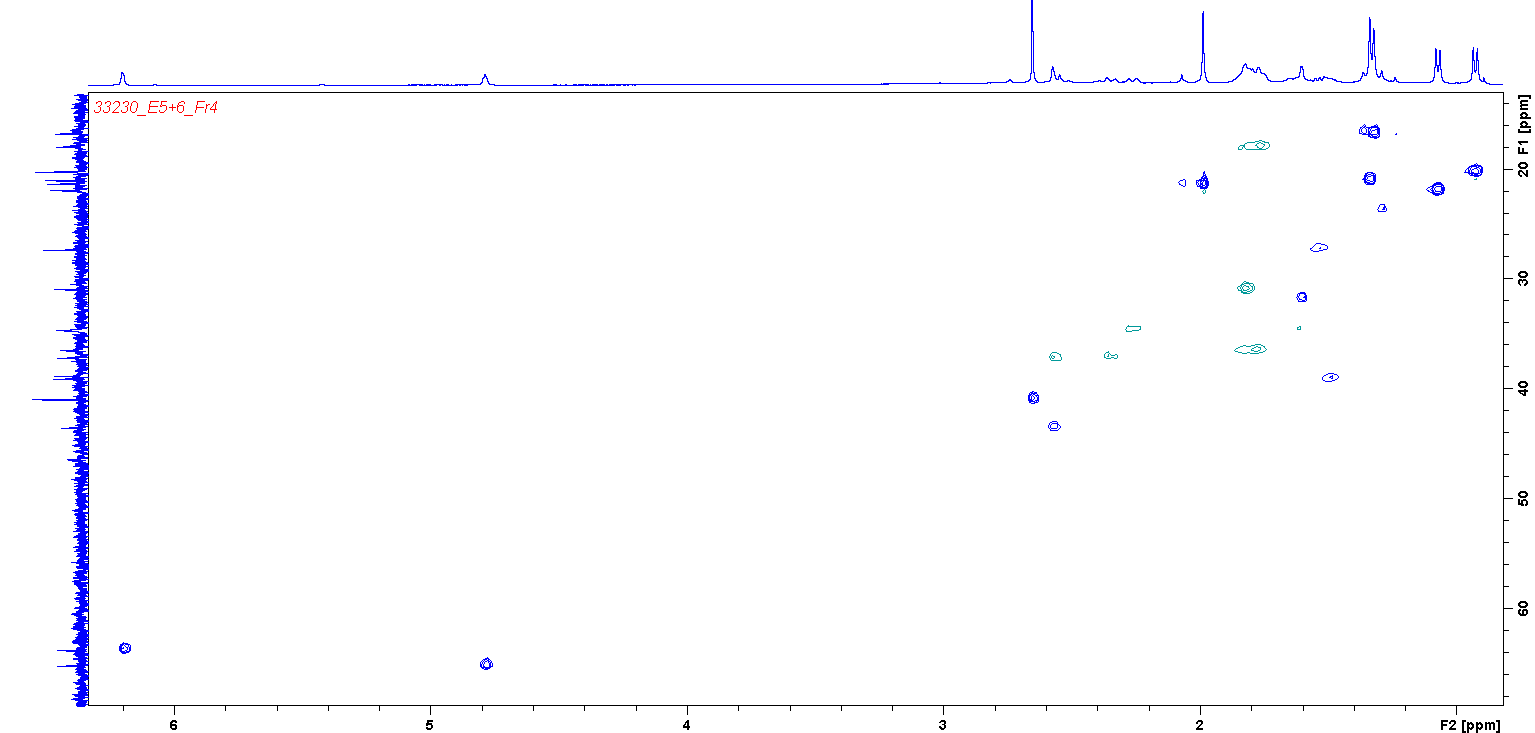


Figure S58. HSQC spectrum of **12**


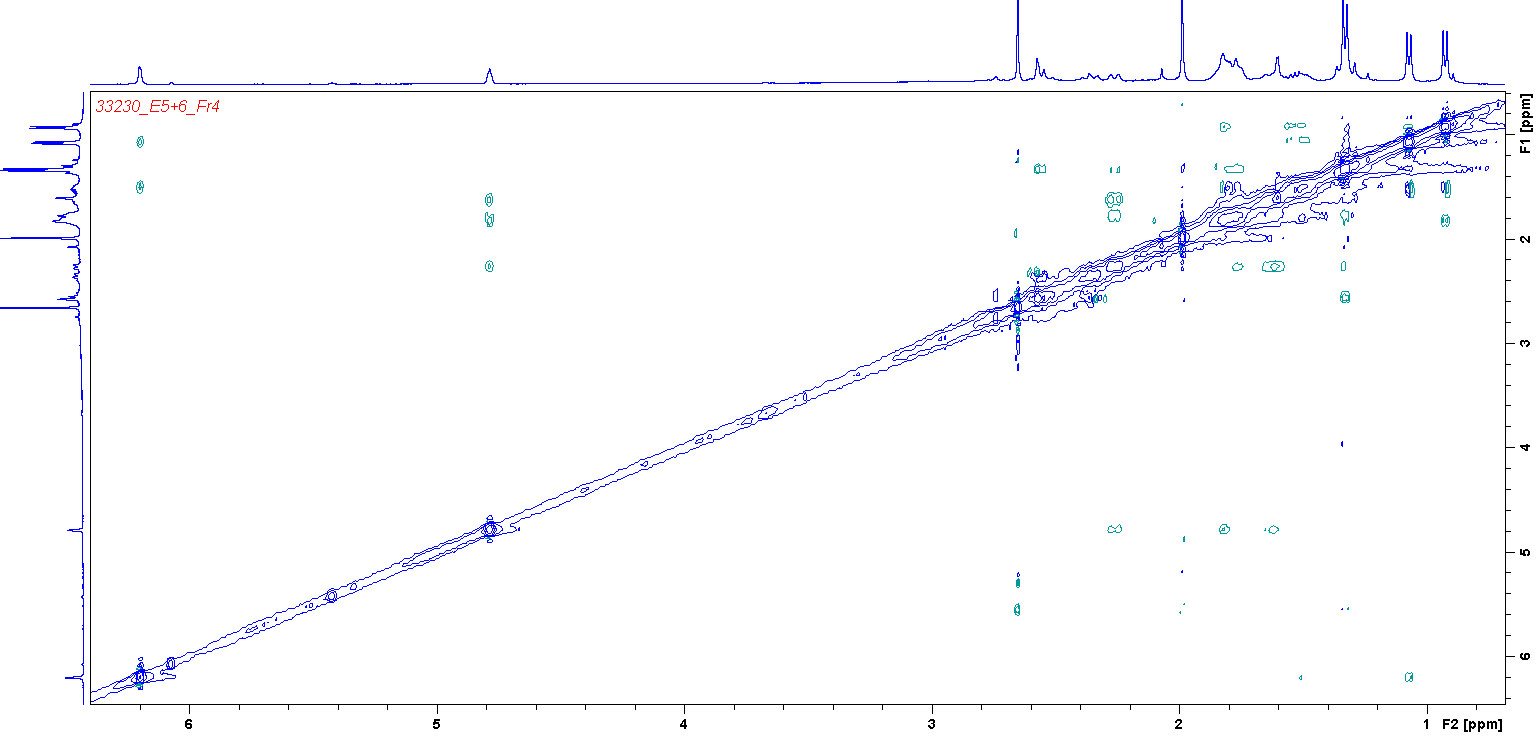


Figure S59. NOESY spectrum of **12**


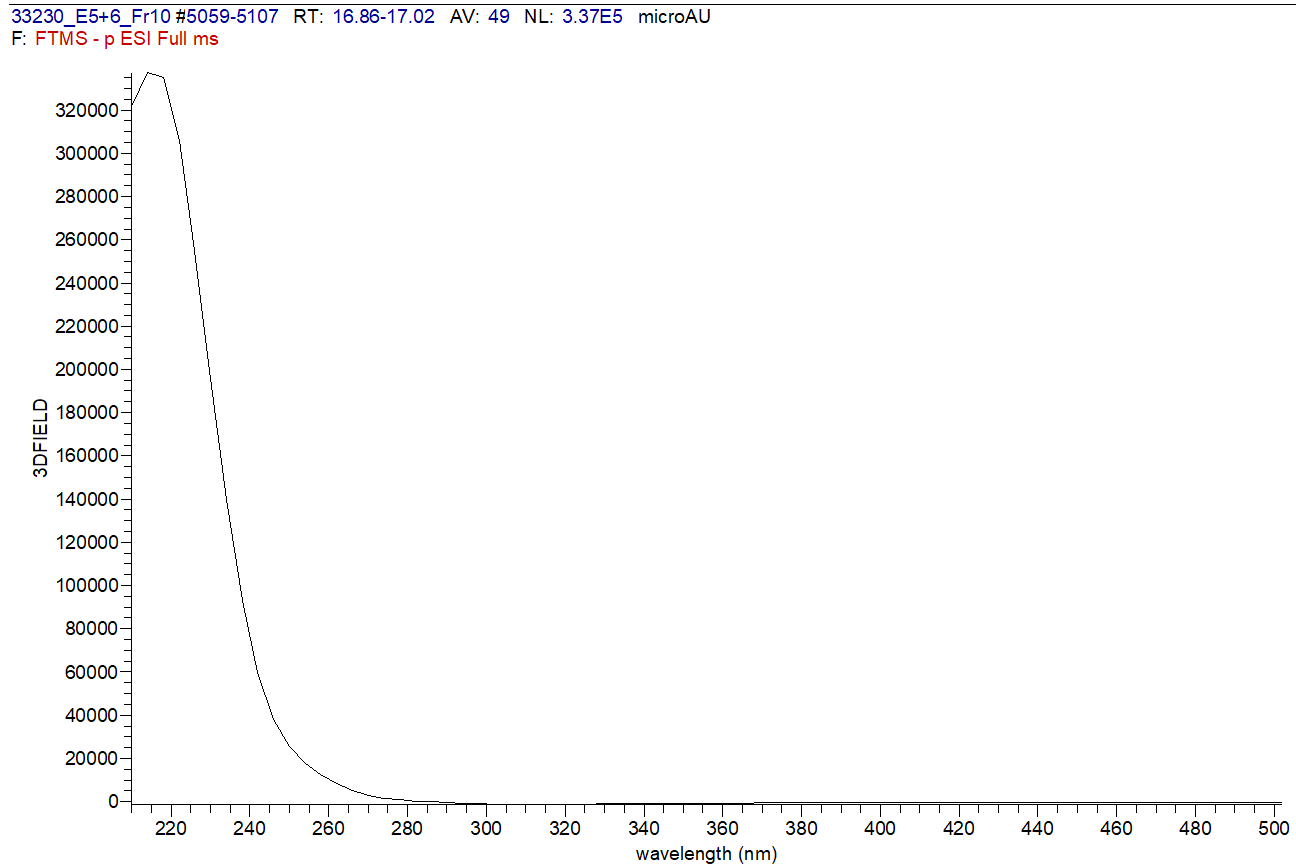


Figure S60. UV spectrum of **13**


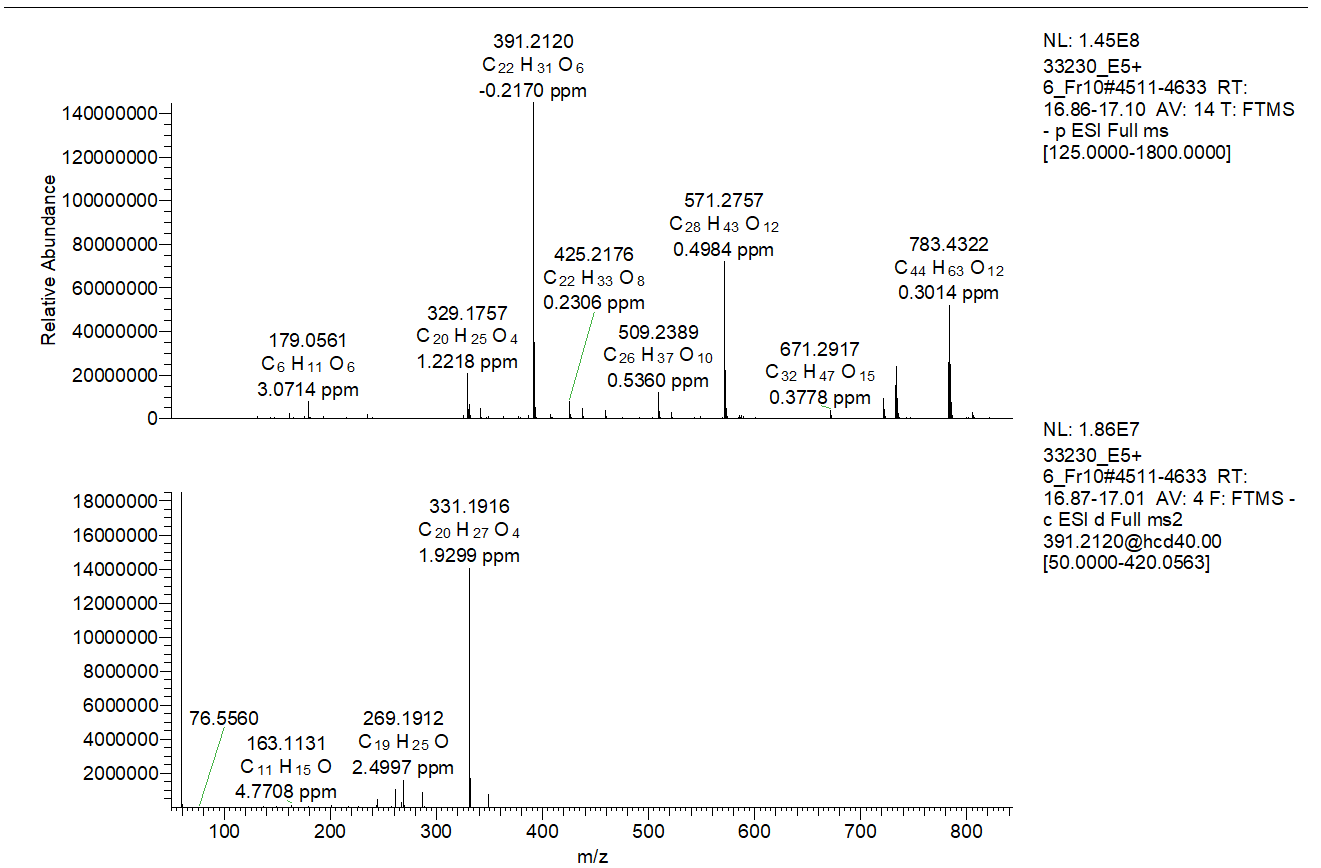


Figure S61. Full (-)-HRESI mass and MS2 spectra of **13**


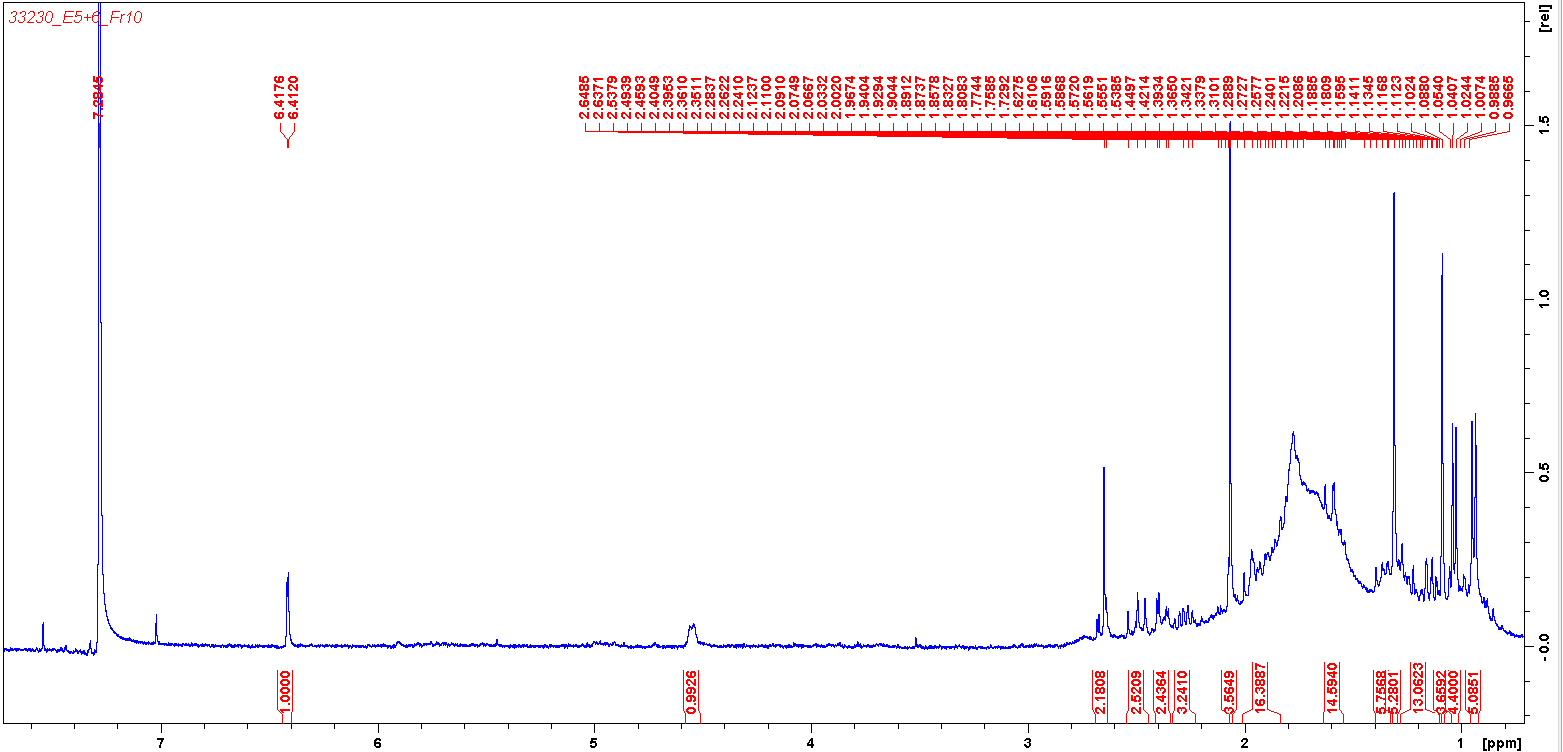


Figure S62. ^1^H NMR spectrum (400 MHz, CDCl_3_) of **13**


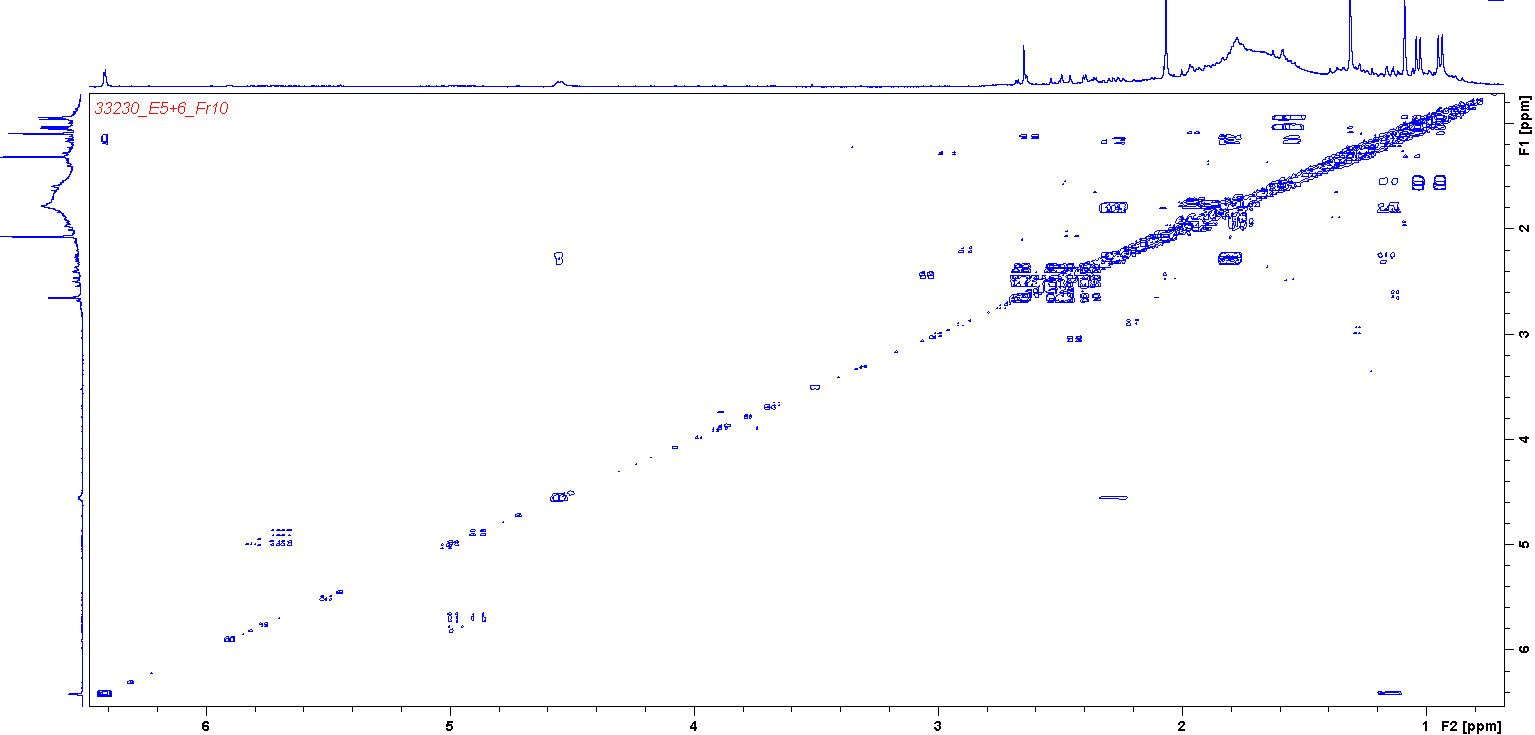


Figure S63. ^1^H,^1^H COSY spectrum of **13**


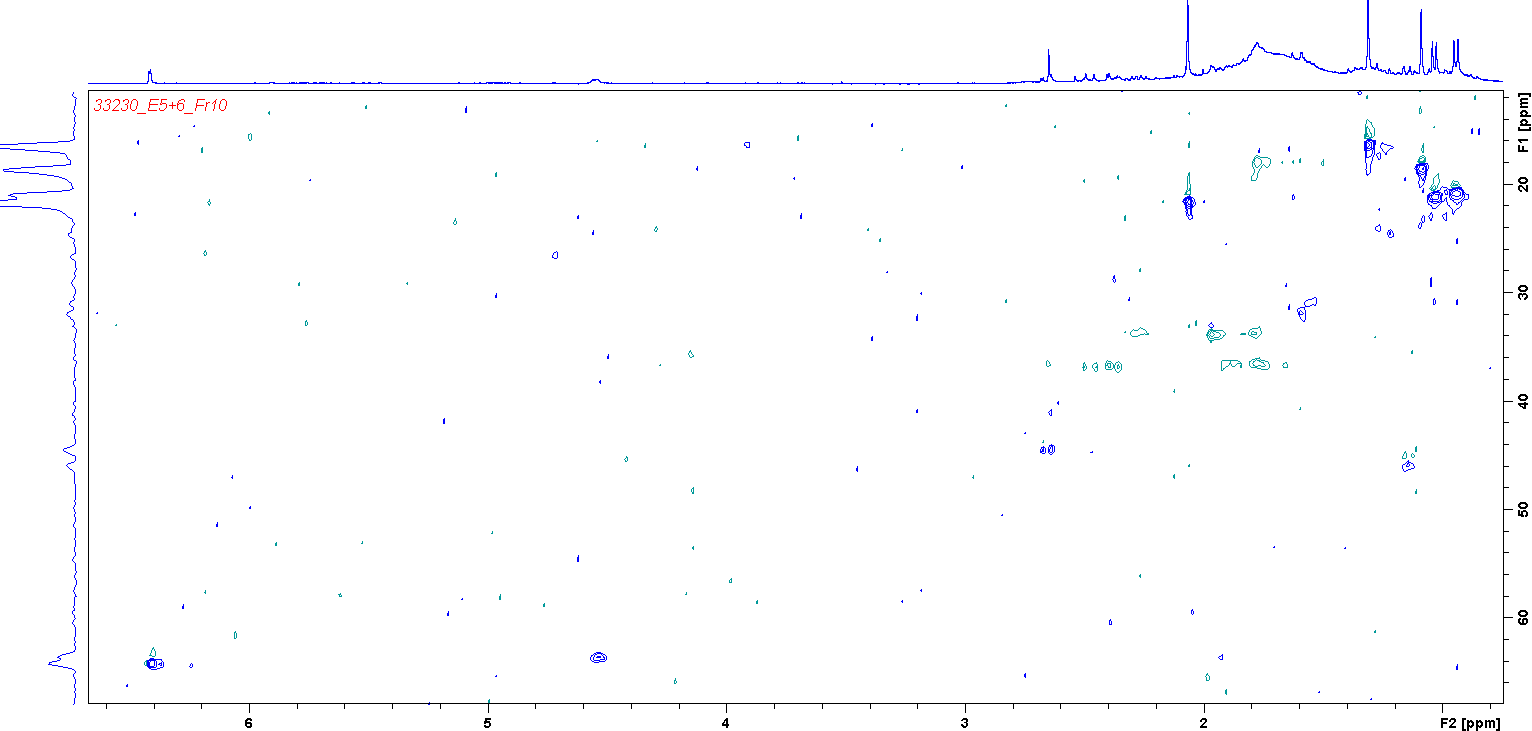


Figure S64. HSQC spectrum of **13**


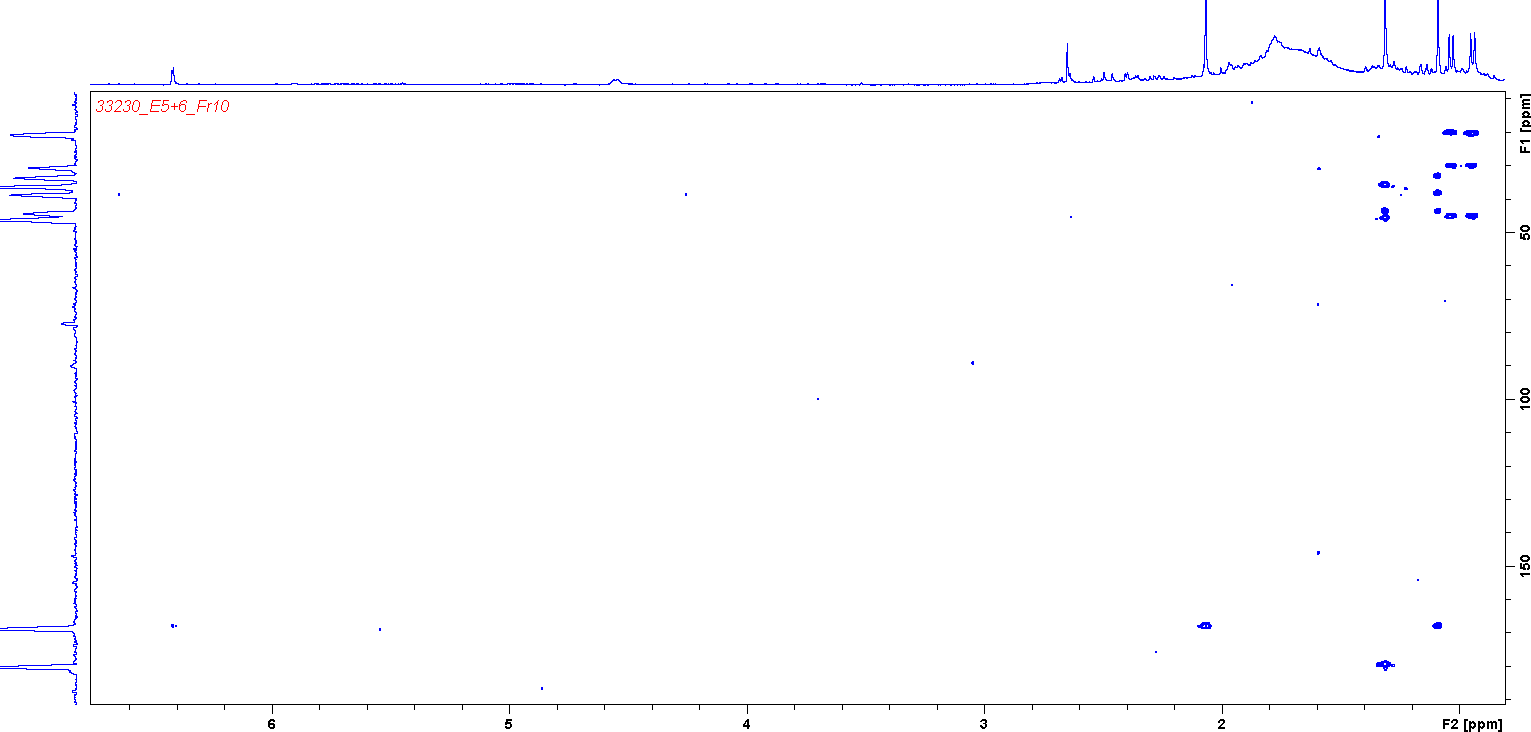


Figure S65. HMBC spectrum of **13**


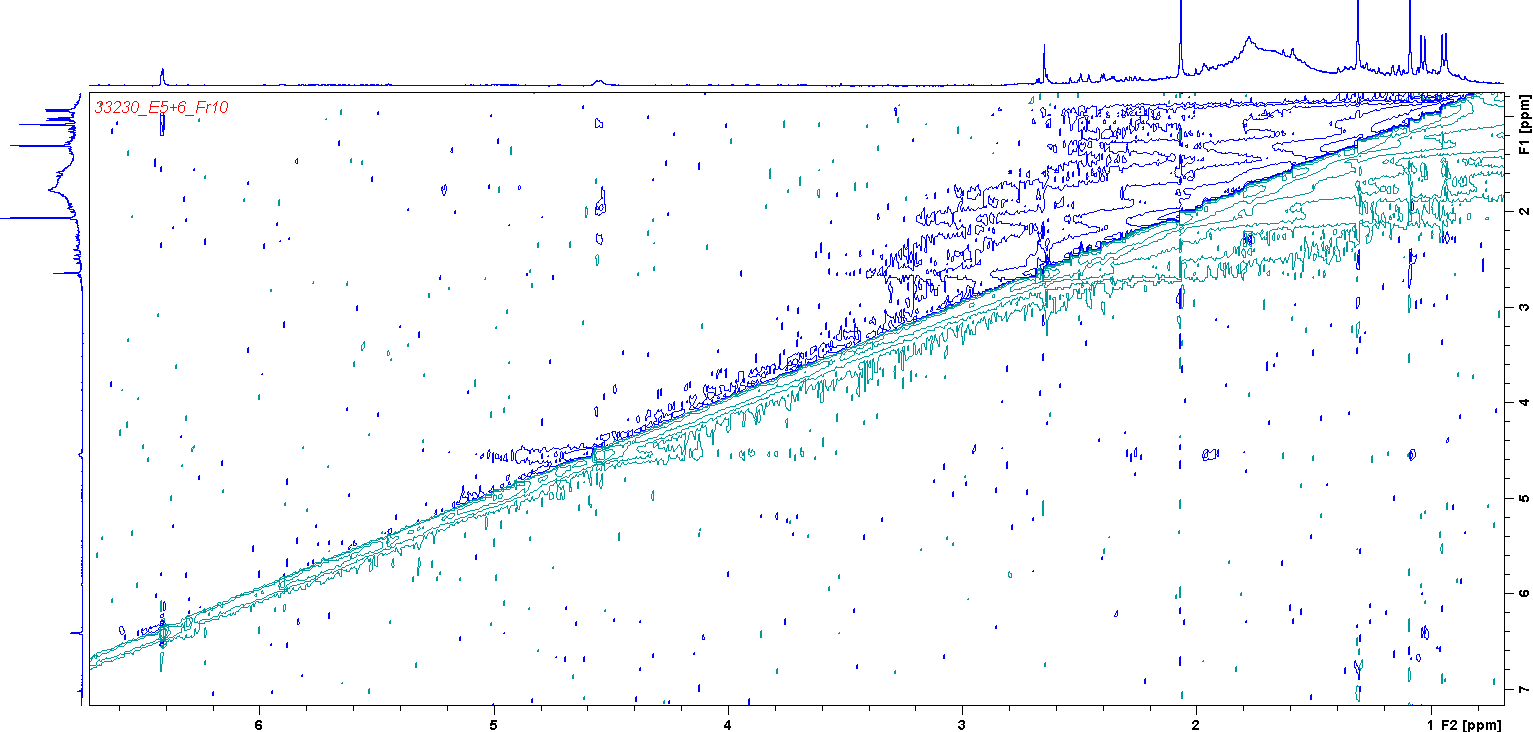


Figure S66. NOESY spectrum of **13**


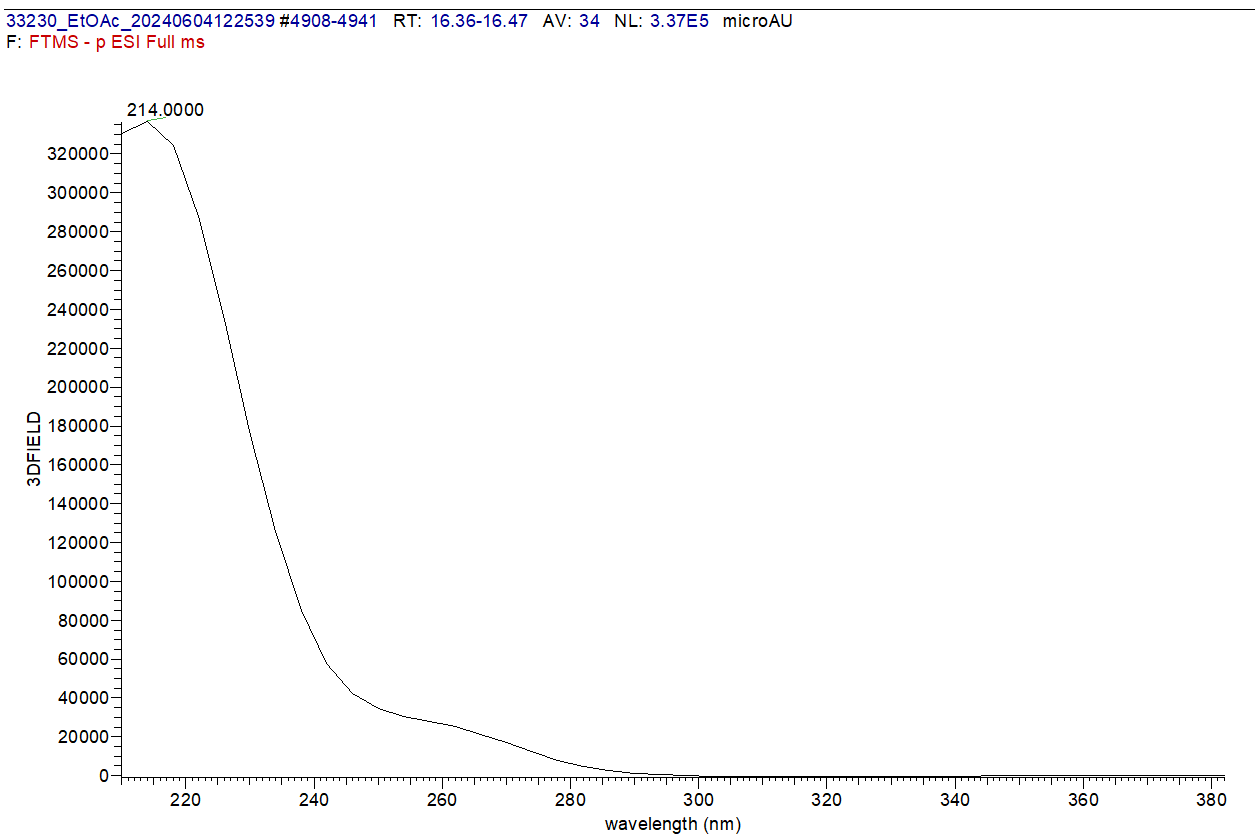


Figure S67. UV spectrum of **14**


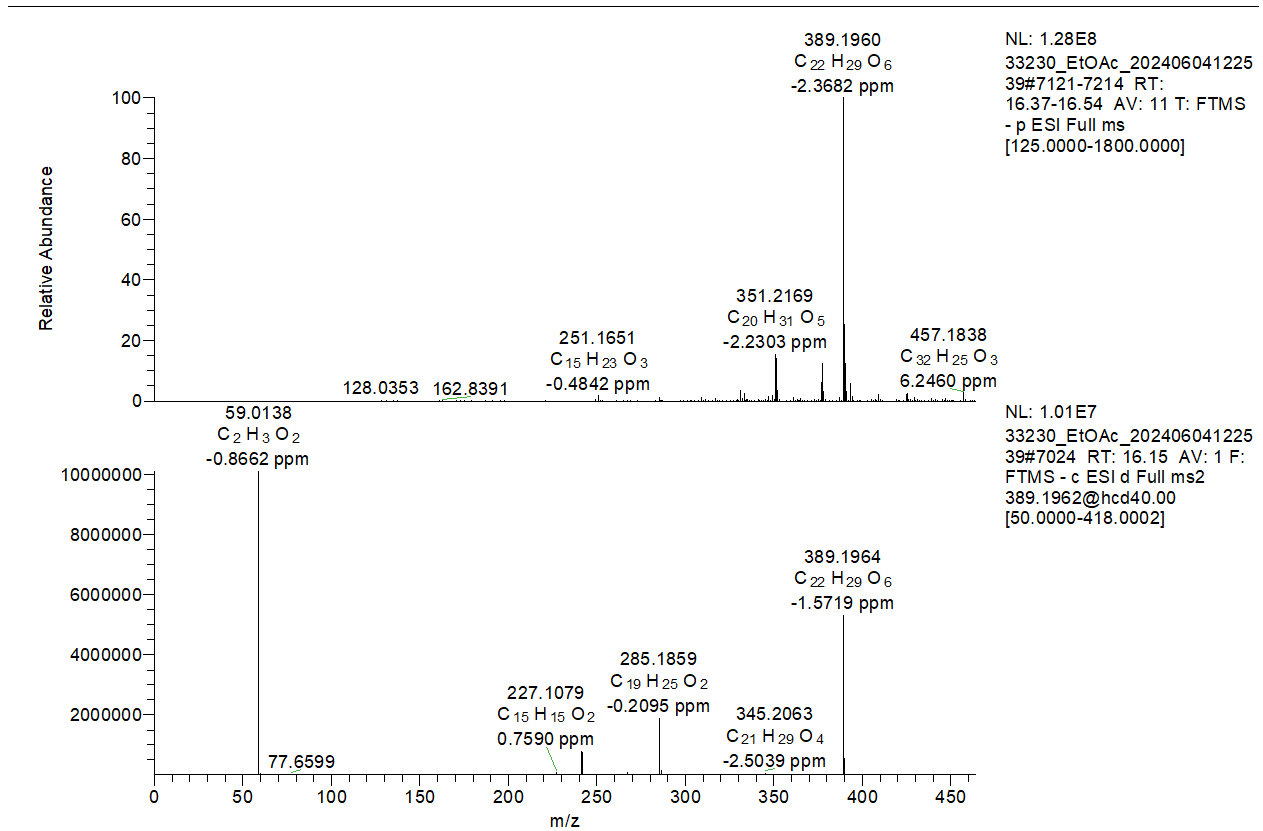


Figure S68. Full (-)-HRESI mass and MS2 spectra of **14**


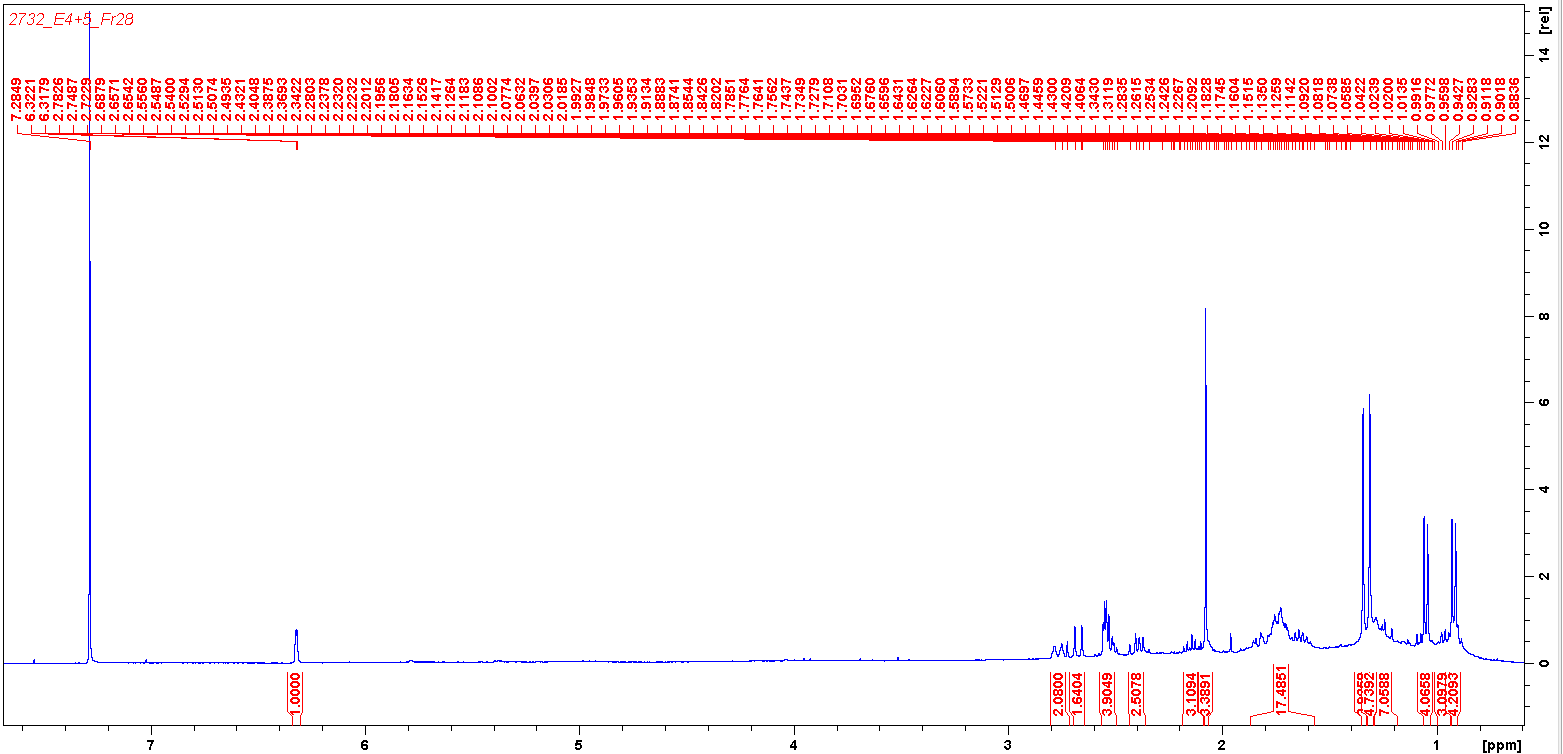


Figure S69. ^1^H NMR spectrum (400 MHz, CDCl_3_) of **14**


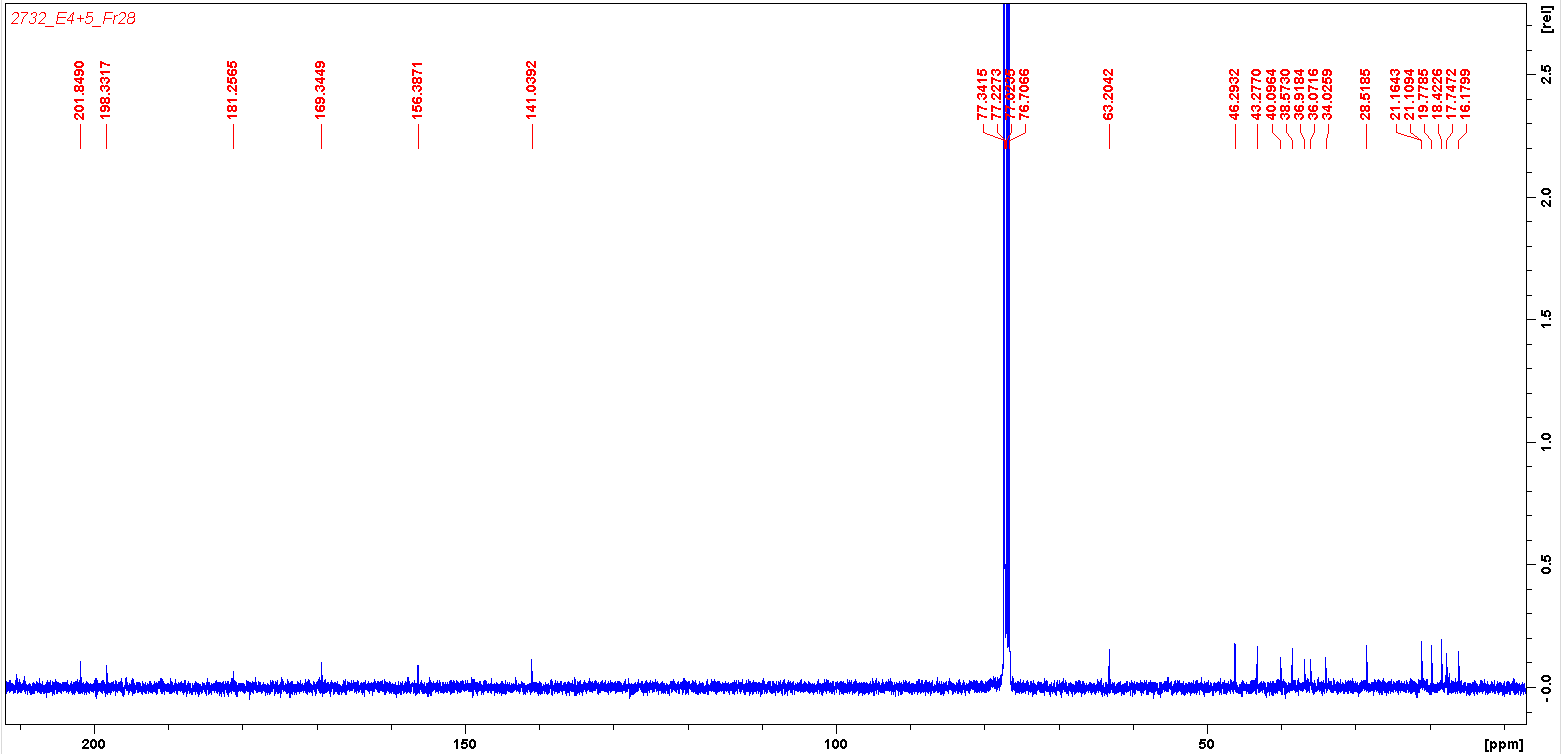


Figure S70. ^13^C NMR spectrum (100 MHz, CDCl_3_) of **14**


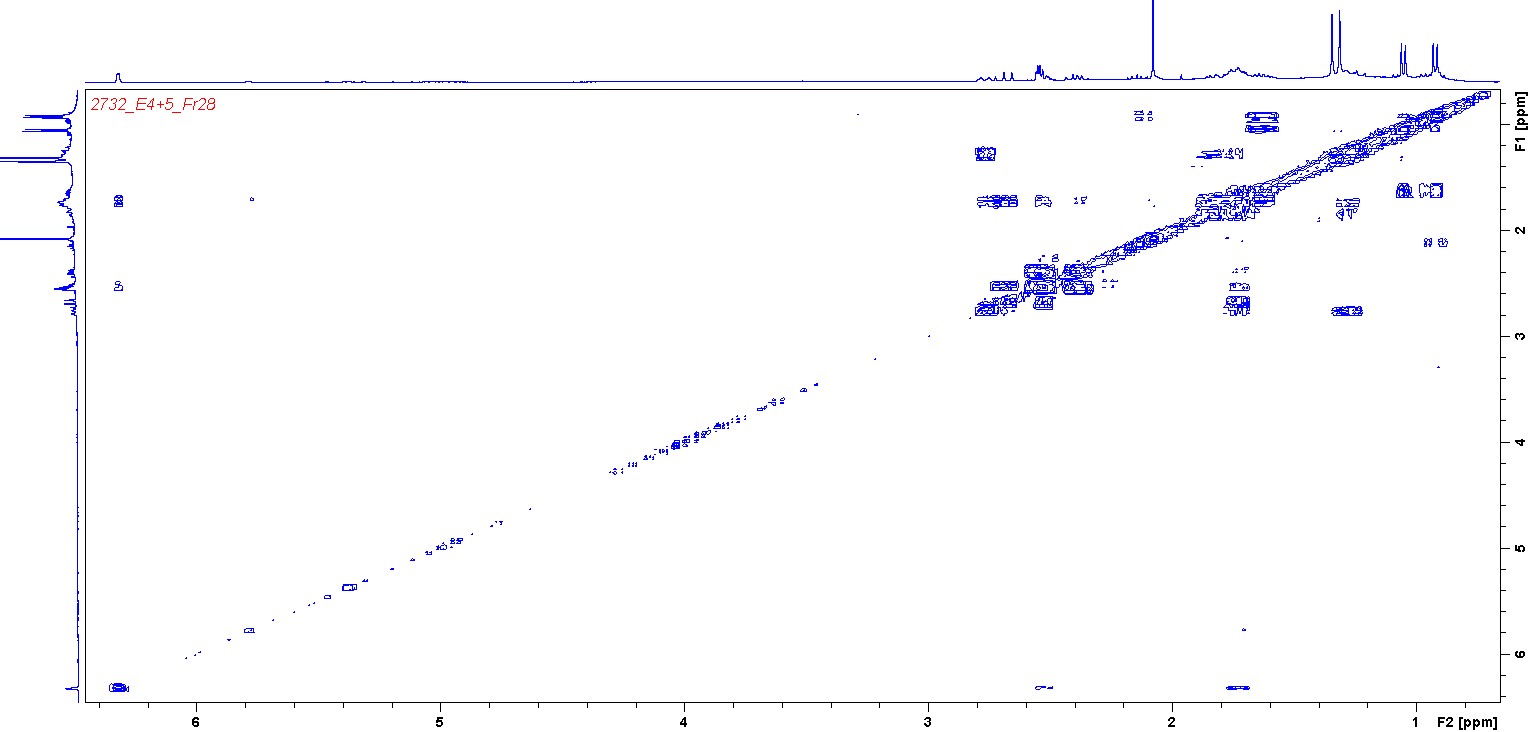


Figure S71. ^1^H,^1^H COSY spectrum of **14**


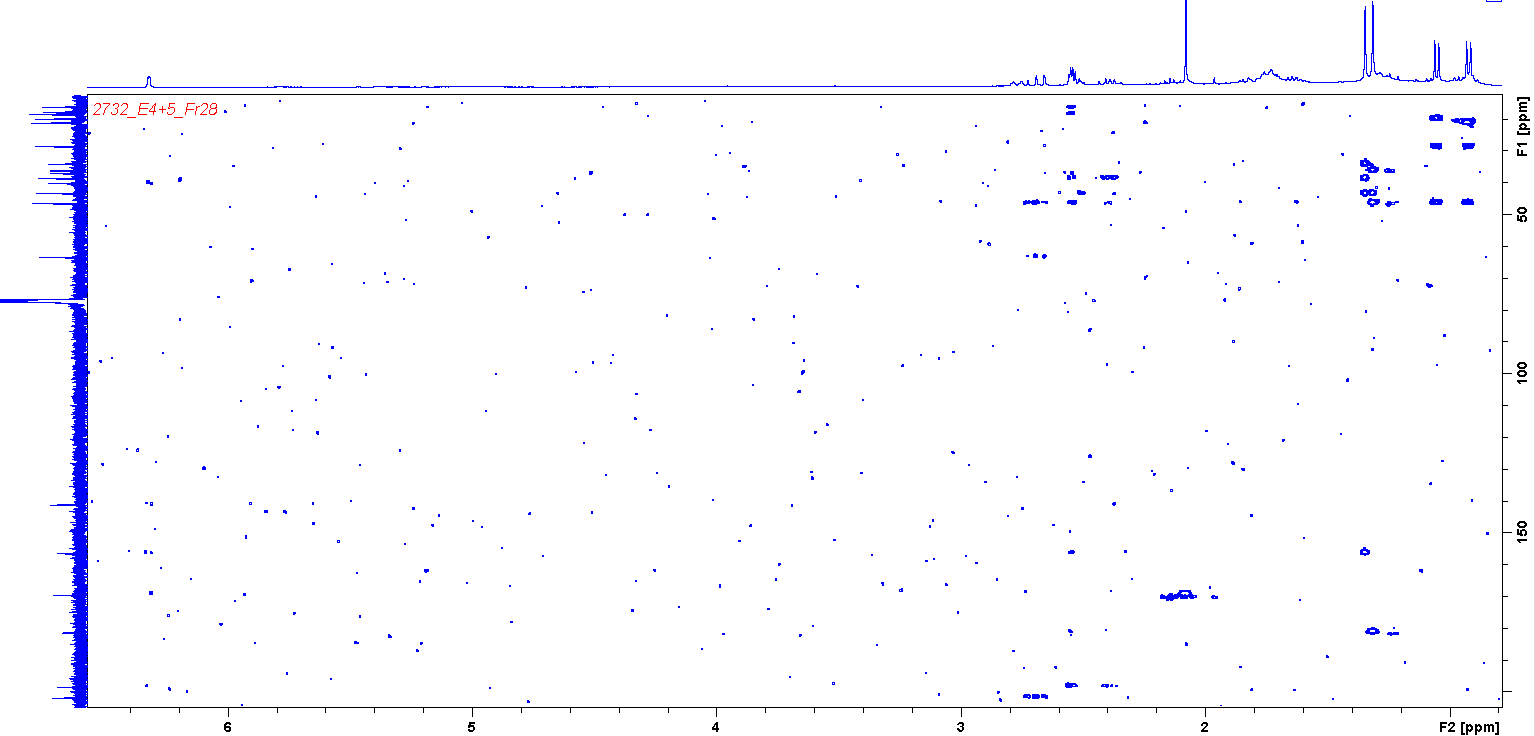


Figure S72. HMBC spectrum of **14**


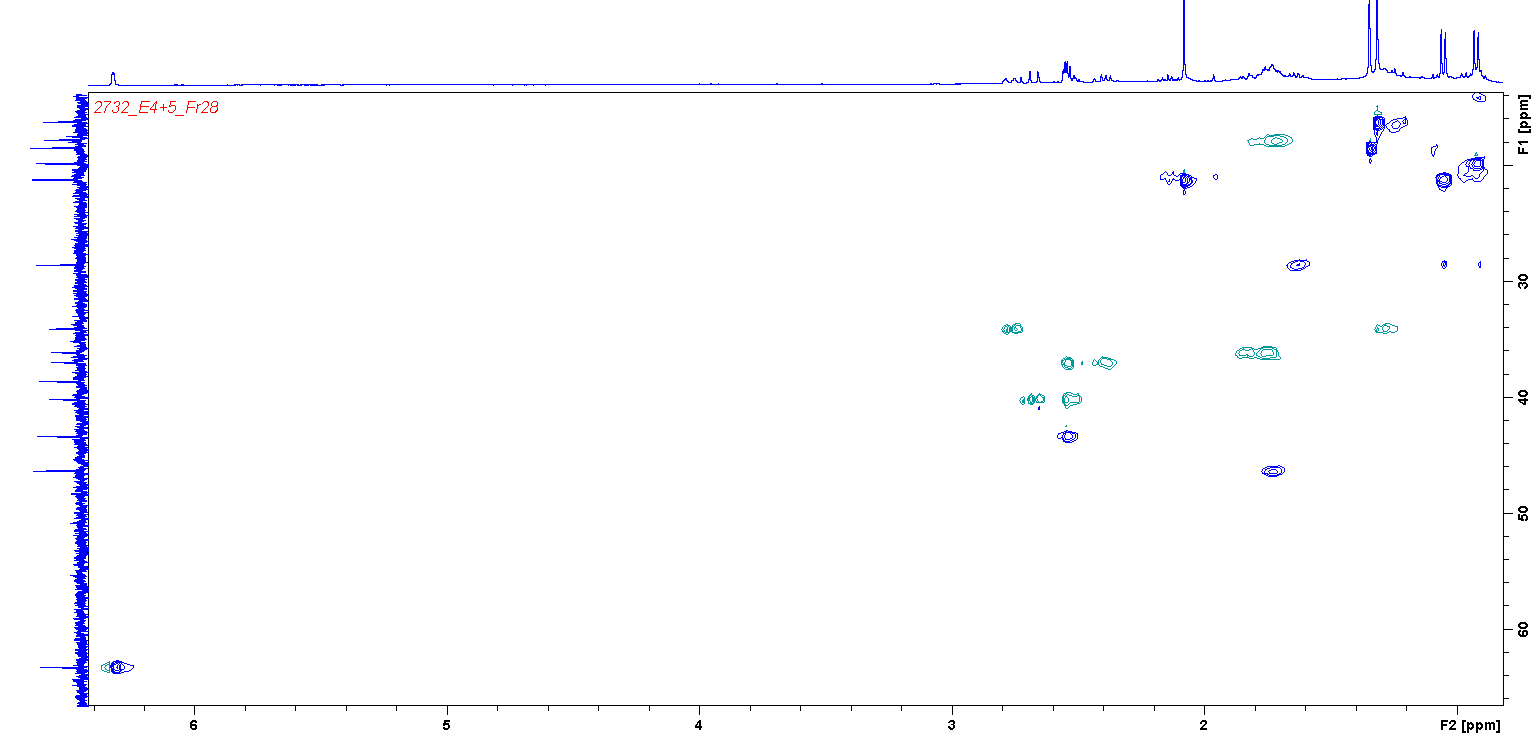


Figure S73. HSQC spectrum of **14**


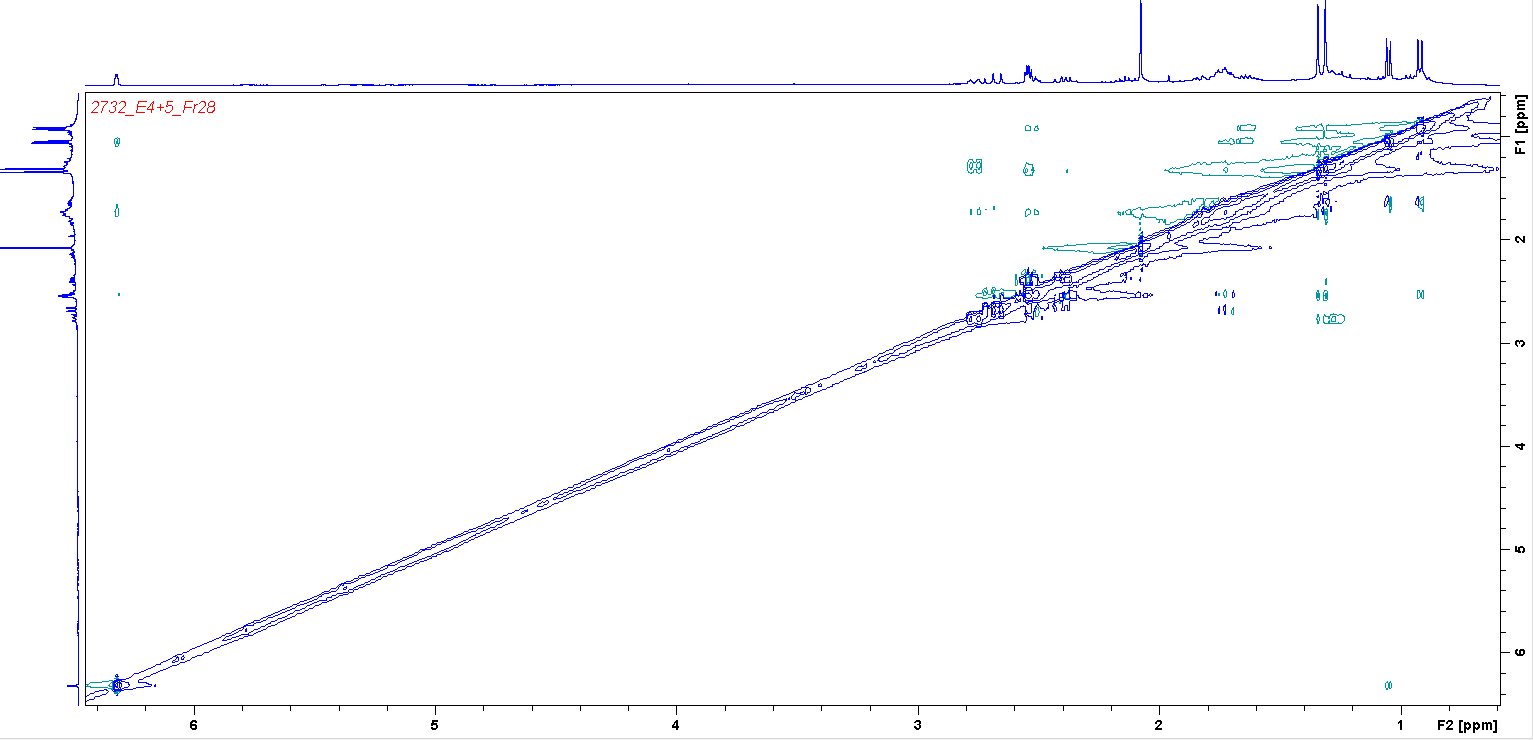


Figure S74. NOESY spectrum of **14**


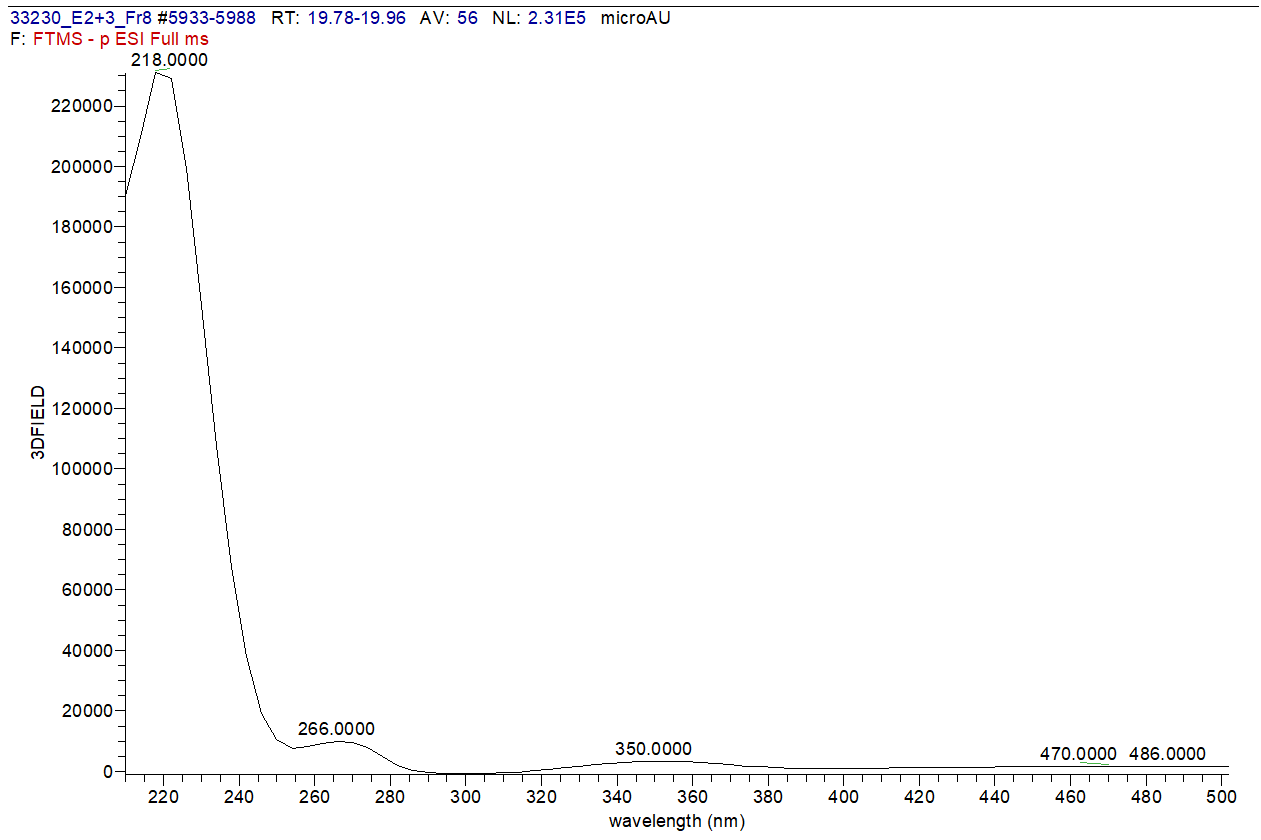


Figure S75. UV spectrum of the mixture of **15**+**16**


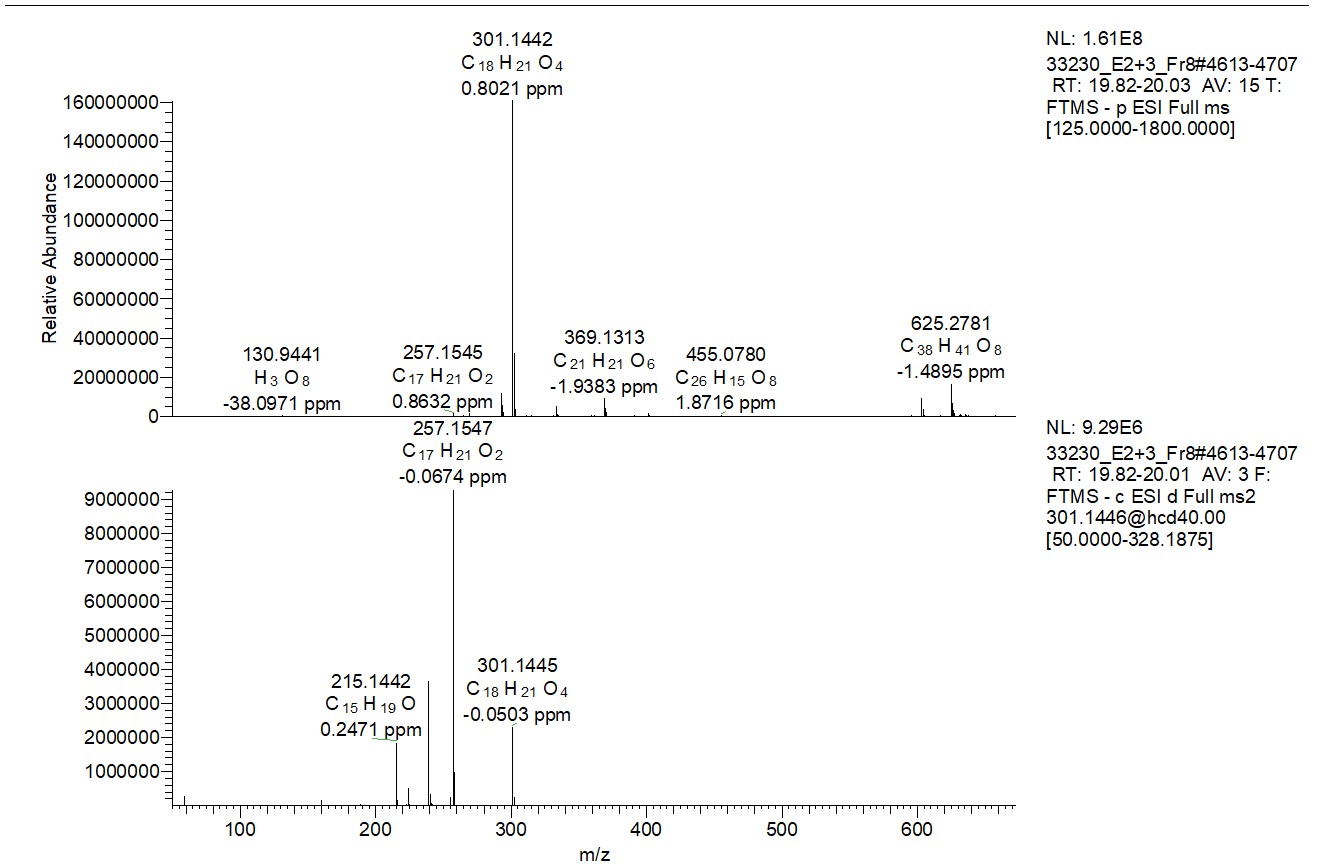


Figure S76. Full (-)-HRESI mass and MS2 spectra of **15**


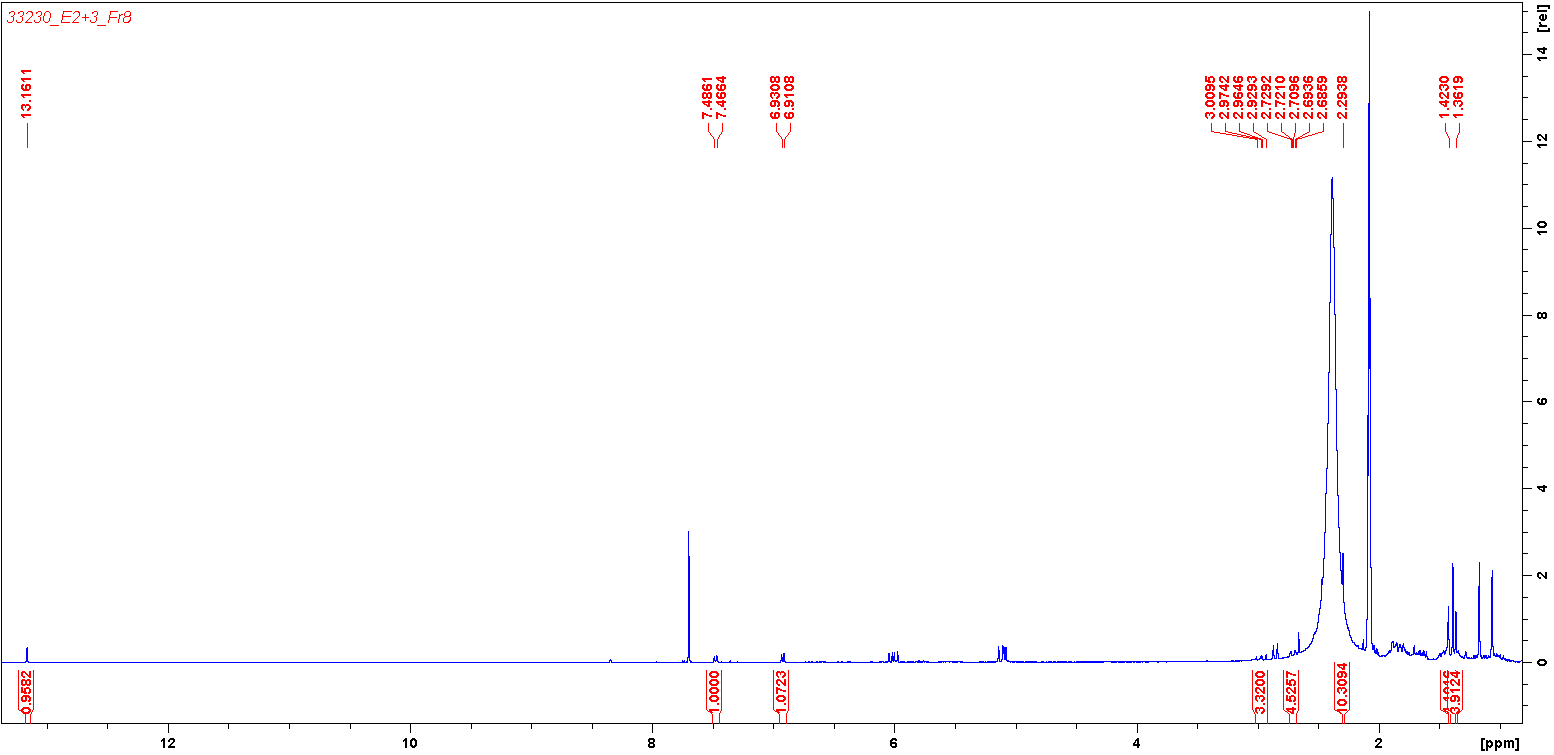


Figure S77. ^1^H NMR spectrum (400 MHz, CDCl_3_) of the mixture of **15**+**16**


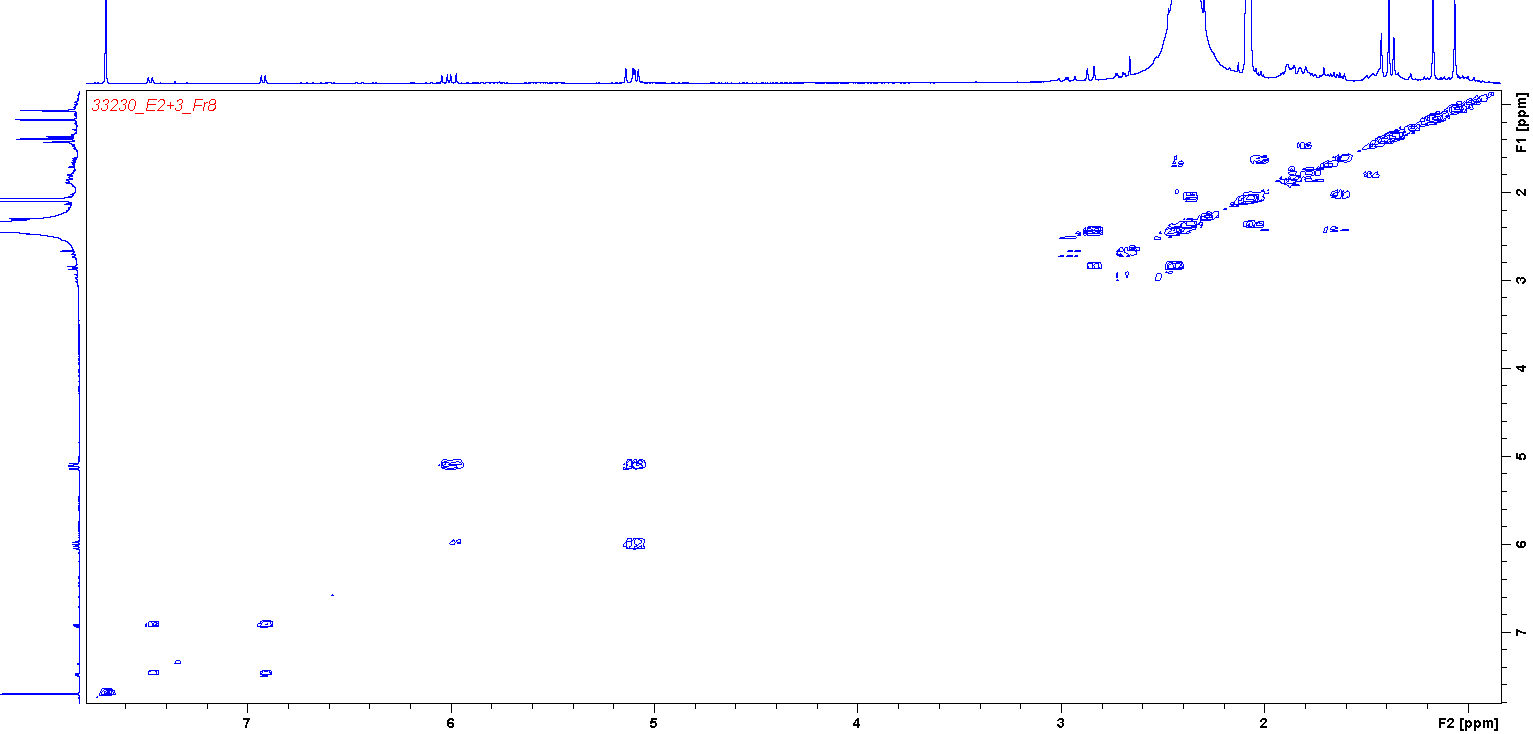


Figure S78. ^1^H,^1^H COSY spectrum of the mixture of **15**+**16**


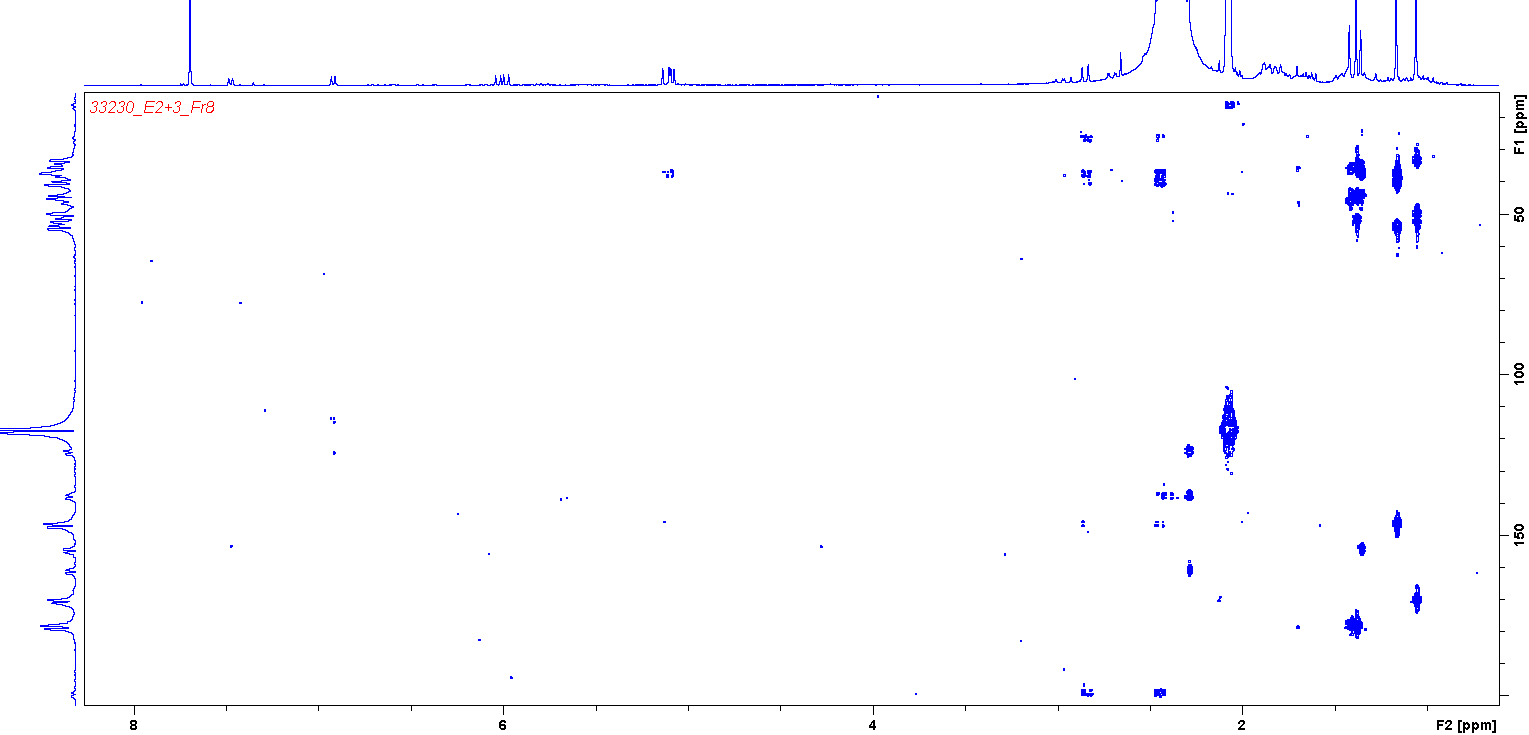


Figure S79. HMBC spectrum of the mixture of **15**+**16**


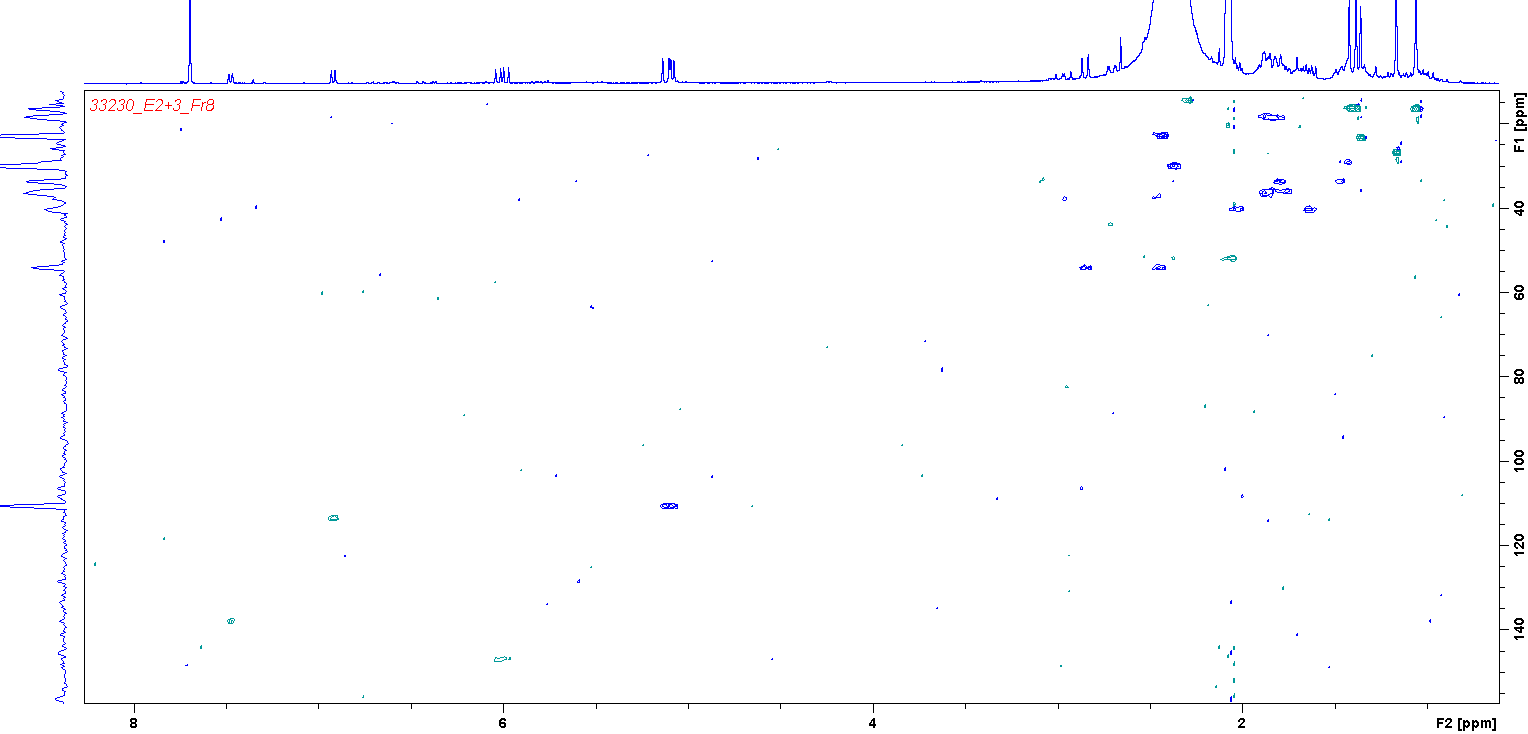


Figure S80. HSQC spectrum of the mixture of **15**+**16**


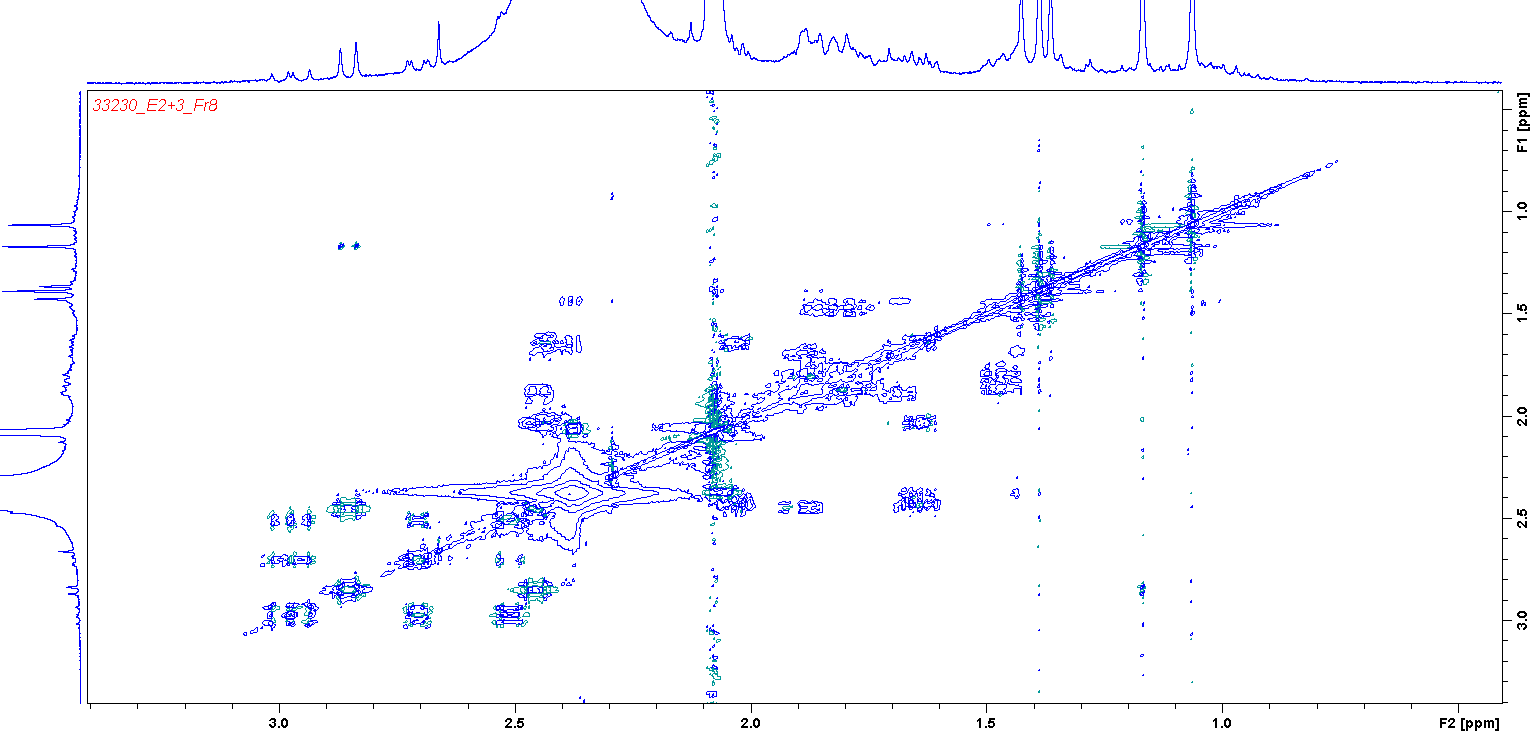


Figure S81. TOCSY spectrum of the mixture of **15**+**16**

**
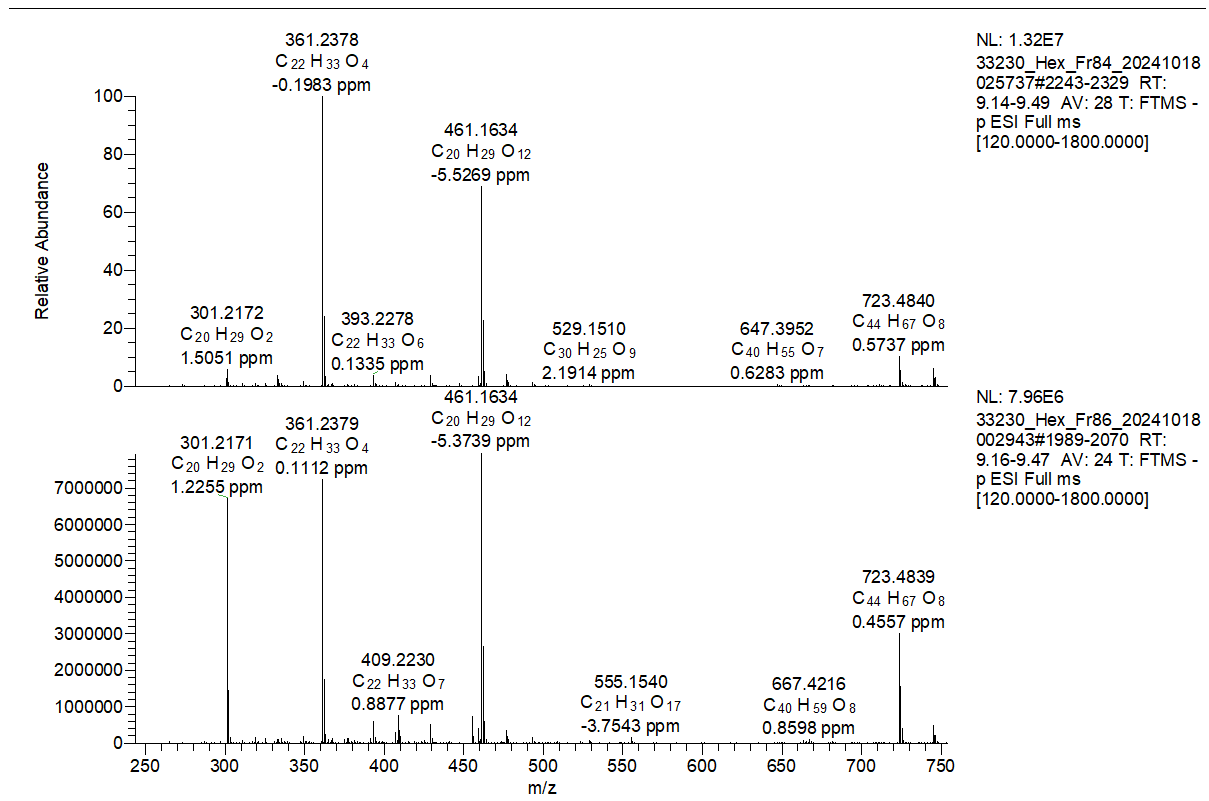
**

**Compound 4**

**Compound 7**

Figure S82. MS1 comparison of compounds **4** and **7**

**
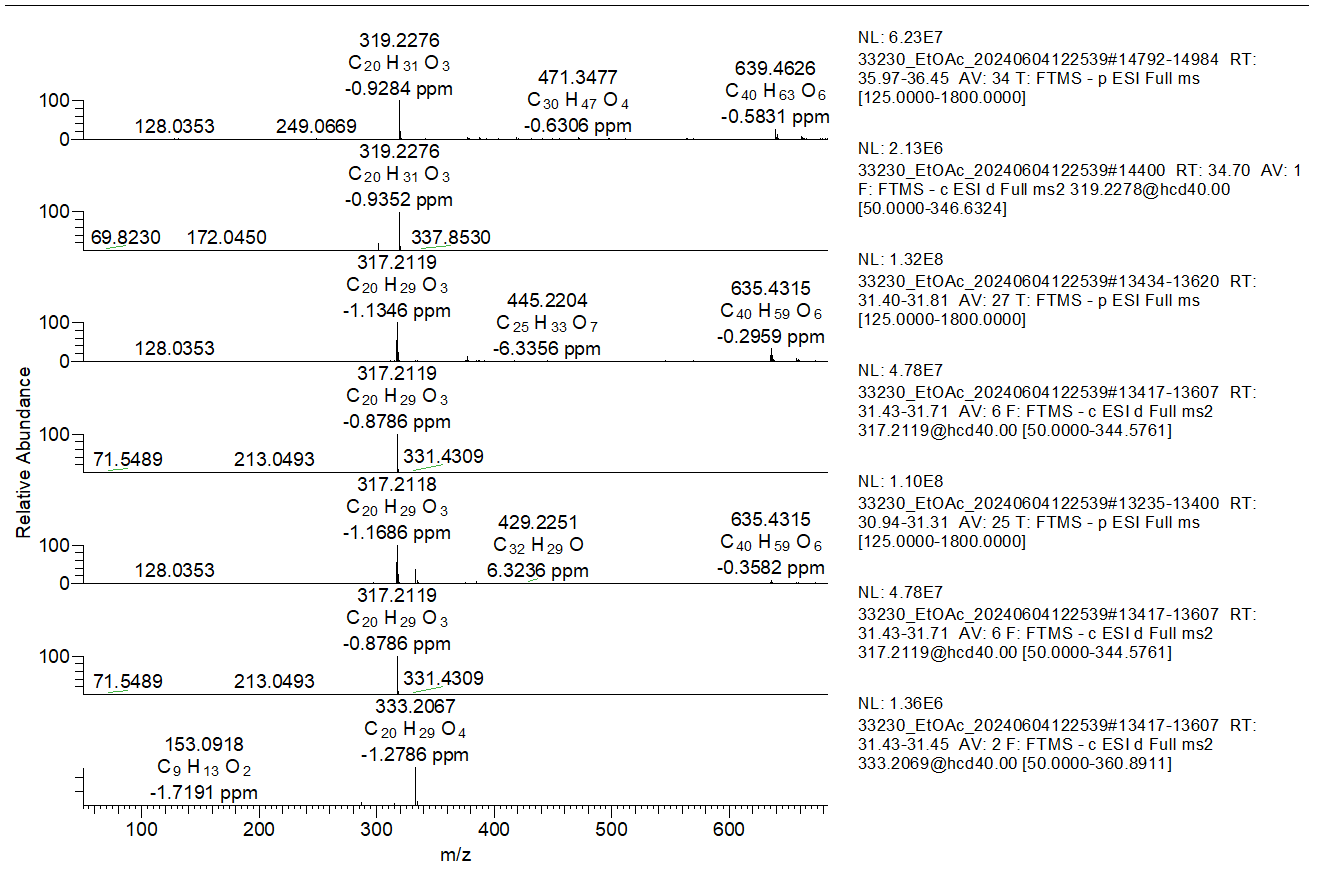
**

**Isopimarane 2**

**Isopimarane 1**

**Abietane 4**

**
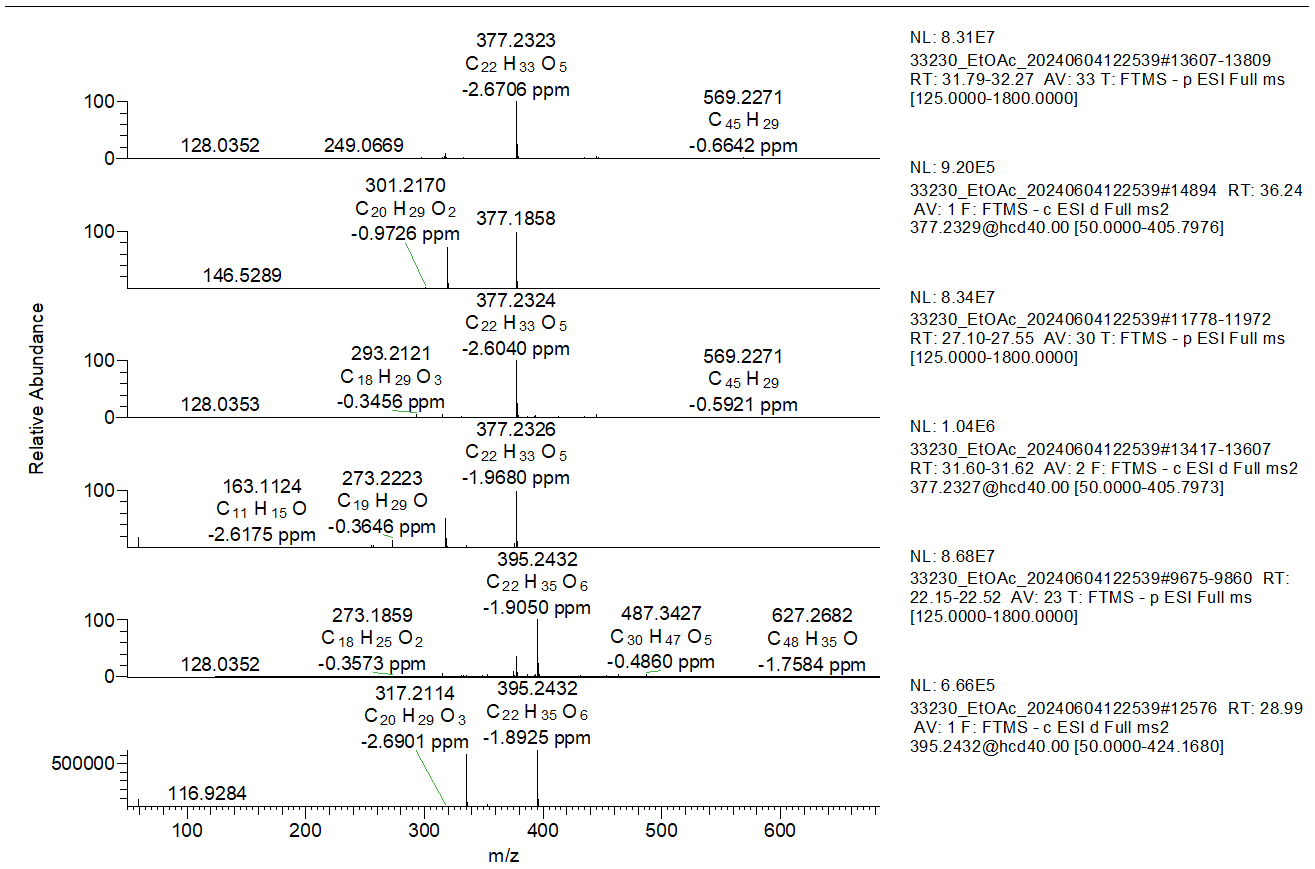
**

**Abietane 1**

**Abietane 2**

**Abietane 3**

Figure S83. MS1 and MS2 of annotated diterpene types of the chromatogram
